# Lattice Stiffening and Delayed Fluorescence in Columnar Liquid‐Crystalline Glasses

**DOI:** 10.1002/cplu.202500738

**Published:** 2026-03-22

**Authors:** Monike da Silva Kutz, Wilson A. de Oliveira, Marília G. Belarmino Cabral, Fabien Durola, Ivan H. Bechtold, Eduard Westphal, Harald Bock

**Affiliations:** ^1^ Centre de Recherche Paul Pascal Université de Bordeaux Pessac France; ^2^ Departamento de Química Universidade Federal de Santa Catarina Florianópolis Santa Catarina Brazil; ^3^ Centre for Organic and Nanohybrid Electronics Silesian University of Technology Gliwice Poland; ^4^ Centre de Recherche Paul Pascal Centre National de la Recherche Scientifique Pessac France; ^5^ Departamento de Física Universidade Federal de Santa Catarina Florianópolis Santa Catarina Brazil

**Keywords:** anisotropic glass, columnar liquid crystal, delayed fluorescence, glass transition, triplet energy

## Abstract

Threefold symmetric ester derivatives of tris(biphenylyl)triazine T2T were made via coupling of tribromo‐triphenyltriazines with boronic acids. With ester substituents only on the outer benzene rings, isotropic glasses were obtained, and ester groups on the inner benzenes were found to be essential for the formation hexagonal columnar mesophases and mesomorphic glasses. Methyl esters form mesomorphic and isotropic glasses with high *T*_g_, whereas ethyl esters have a much lower *T*_g_. At *T*_g_, the column lattice contracts on cooling and becomes fairly resistant to shrinkage on further cooling, whereas the stacking distance within the columns is unaffected by the glass transition. Thus, the glass transition affects the plane of the periodic column lattice of the structure, but not the stacking along the columns. The *T*_1_ energies are close to that of the parent arene T2T, a known host for phosphorescent emitters. Delayed fluorescence is observed at low temperature, that is, without thermal activation, which indicates radiative triplet–triplet annihilation (TTA‐DF). This TTA‐DF is prominent in the derivatives that form mesomorphic glasses and weak in those that do not. Thus, a direct correlation between liquid‐crystalline stacking and fluorescence by TTA is observed.

## Graphical abstract

If the substitution pattern is well‐chosen, semiflexible triaryltriazine esters form glassy columnar liquid crystals whose column lattice distinctively shrinks at the glass transition. The presence of columnar liquid‐crystalline order is found to favor delayed fluorescence by triplet–triplet annihilation.

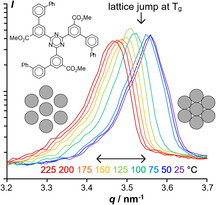

## 1 Introduction

Isotropic organic molecular glasses combine the mechanical robustness of crystalline solids with an absence of grain boundaries and intercrystalline voids and offer the possibility of physical vapor deposition (PVD). They therefore find preferred application in devices such as organic light‐emitting diodes for flat panel displays and in organic electronics in general [1]. The entropy content of glasses produced by cooling the melt diminishes slowly on aging, giving rise to denser and locally more ordered glasses over long time spans. This aging process can be accompanied by a slow improvement of electronic properties such as charge transport and mobility of excited states. PVD, in contrast, allows accessing low‐entropy, high‐density glasses directly (i.e., over the much shorter time span of the evaporation process) via the control of substrate temperature and sublimation speed [2].

Liquid crystals (LCs), especially columnar LCs, made of organic molecules offer much higher charge and exciton mobilities than isotropic liquids and glasses but generally lack the mechanical solidity of isotropic glasses [3]. In contrast to these, LCs are anisotropic, such that depending on their orientation on a substrate, preferred directions of transport as well as of light absorption and emission can be chosen.

A glass transition may not only be observed in supercooled isotropic liquids but also in LCs [4, 5]. Due to the partially liquid nature, a glass transition can occur, freezing in the local positioning of molecules or molecular clusters. If such a glass transition occurs, the LC transitions from a shearable wax to a rigid solid, without nucleation of crystals. In contrast to isotropic glasses, liquid‐crystalline glasses may present grain boundaries, but these are less associated with intergrain voids as in crystals, and, in contrast to grain boundaries in crystals, they may be minimized by annealing with surface alignment above the glass transition temperature *T*_g_. Even during PVD at a substrate temperature below *T*_g_, annealing processes are prominent in the fluid upper layer of the film during deposition, allowing uniform surface orientation throughout the growing film [2, 6]. Whereas the ease with which uniform alignment can be obtained in films of columnar LCs is very much case‐dependent, the domain diameter in uniformly face‐on surface‐oriented thin films of discotic columnar LCs is typically much larger than the film thickness, and thus, domain boundaries occur only rarely on a straight path through the film [7, 8, 9].

A convenient columnar liquid‐crystalline glass should not only have its glass transition at high enough temperature to allow the glass state to persist at the local temperatures of organic devices during operation; it should also be optically rather neutral, that is, be nonabsorbing at visible wavelengths and have high enough energies of the first excited singlet and triplet states (*S*_1_ and *T*_1_) to be noninterfering with potential emissive dopants in thermally activated delayed fluorescence (TADF) organic light‐emitting diodes (OLEDs) and phosphorescent OLEDs (PhOLEDs). For comparison between deposition and alignment by PVD and by cooling from the melt, it is also desirable to have transition temperatures from the LC and the crystal states to the isotropic liquid—clearing point (*T*_cl_) and melting point (*T*_m_)—that are well below the decomposition temperature.

A practical limit molecular weight for PVD is about 1200 g·mol^−1^, a practical decomposition limit for clearing, and melting point is at most 300°C, and a tough, admittedly hard‐to‐reach, target for a comfortably large energy gap between any excited state and the ground state is 3 eV to allow hosting deep‐blue‐emitting dopants [10].

The common steric criteria for a columnar LC (flat with a rigid center and a fluid periphery) and for a high‐temperature glass (configurationally flexible in three dimensions, but no or little fluid parts) are only partially compatible.

Columnar liquid‐crystalline glasses close to room temperature by nonpolymeric mesogens have been previously observed, among others, with dimeric systems such as twinned hexaalkoxy‐triphenylenes [11, 12, 13, 14, 15, 16, 17, 18, 19] and with large monomeric mesogens with voluminous anchor‐like side chains [20]. A donor–acceptor‐type heterocyclic system that has been found to lead to hexagonal columnar glasses when surrounded by three alkoxy‐bearing aryl substituents is tris(1,2,3‐triazolyl)‐1,3,5‐triazine; here, the disks are often formed by two paired molecules in low‐symmetry conformations, which may favor intertwining and vitrification [21, 22].

Aiming for a large bandgap excludes condensed polycyclic arene fragments, which are commonly chosen in the design of columnar LCs. Therefore, only single bonds should be considered as links between monocyclic arene rings. *Meta* links such as in *m*‐terphenyl are to be preferred because they allow larger bandgaps than *para*‐links such as in *p*‐terphenyl [23].

**SCHEME 1:**
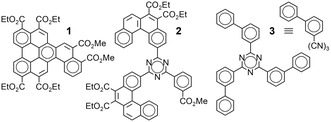
Glassy columnar esters **1** and **2** and high‐triplet‐energy glass **3** (a.k.a. T2T), with short notation of **3** as used hereafter for its derivatives.

To minimize the fluid alkyl periphery R of columnar mesogens that is detrimental to a high glass transition temperature (*T*_g_), we have found in the past that alkoxycarbonyl substituents –CO_2_R are particularly efficient: In contrast to more conventional alkyl‐chain substituents such as alkoxy and alkanoyloxy where at minimum pentyl is generally necessary to induce mesomorphism, alkoxycarbonyl as short as –CO_2_Et and –CO_2_Me are sufficient to induce a columnar mesophases when attached to a fairly circular arene core. This allowed us to obtain an above‐ambient *T*_g_ in the columnar mesophases of several tetra‐ to hexa‐alkoxycarbonyl‐arenes, most notably with phenanthroperylene derivative **1** (*T*_g_ of 120°C, Scheme 1) [24, 25] and triaryltriazines such as **2** (*T*_g_ of 67°C) [26].

Tris(biphenyl‐3‐yl)triazine **3**, commonly abbreviated T2T, with a *T*_g_ of 55°C [27] and a reported *T*_1_ energy of 2.80 eV, has found use as high‐*T*_1_ matrix or comatrix for green and sky‐blue PhOLEDs [28, 29] and as hole‐blocking layer in TADF OLEDs [30]. Thus, **3** is known to have much a higher *T*_1_ energy than condensed polycyclic columnar arenes such as **1** and **2**.

Given the similarity between **3** and **2**, we were hopeful that alkoxycarbonyl derivatives of **3** would show a columnar LC mesophase with a high *T*_g_.

## 2 Results and Discussion

**SCHEME 2:**
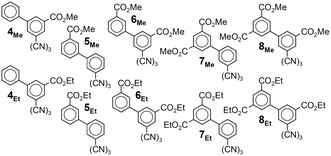
The ten alkoxycarbonyl derivatives of **3** investigated here.

To induce a columnar mesophase, ideally with a high *T*_g_ within the temperature range of the mesophase and without lowering the energy levels of the excited states *S*_1_ and *T*_1_, we introduced methyl or ethyl ester substituents in some or all of the available *meta* positions of **3**. We avoided derivatives with ester substituents in *para* or *ortho* positions, to minimize the effect of the carboxylic substituents on *S*_1_ and *T*_1_. For synthetic simplicity, we only aimed at C3‐symmetric targets in which all three biphenyl arms are identically substituted, that is, the ten derivatives **4**_**Me**_ to **8**_**Et**_ (Scheme 2).

**SCHEME 3:**
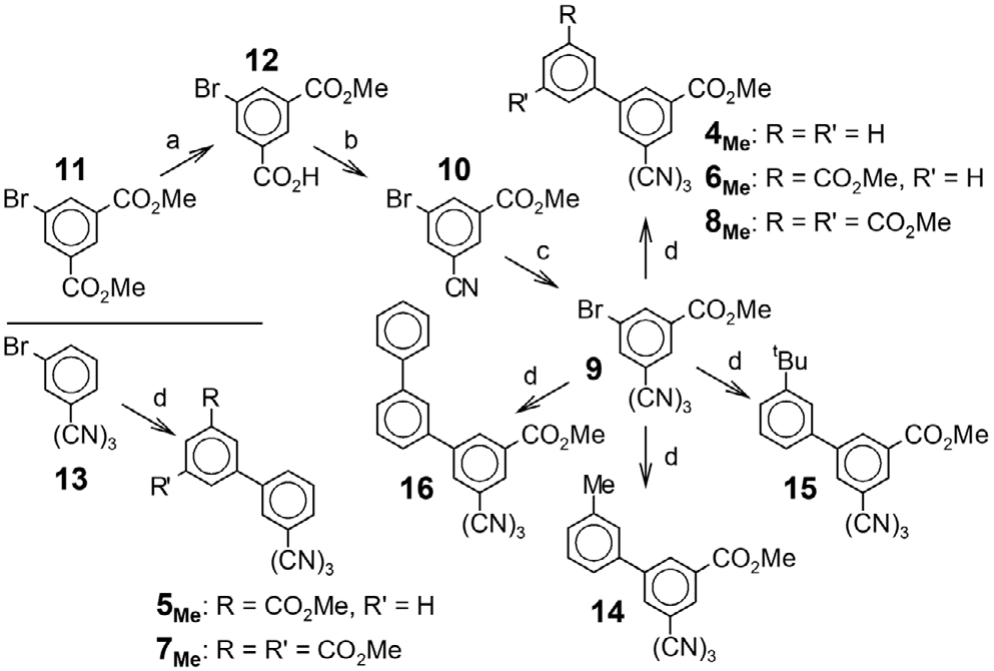
Synthesis of the target compounds **4**_**Me**_ **–8**_**Me**_ and **14** **–16**. a: LiOH (1 eq in MeOH); b: 1. SOCl_2_, 2. NH_3_(aq), 3. POCl_3_; c: TfOH; d: ArB(OH)_2_, Pd(PPh_3_)_4_, K_2_CO_3_.

We synthesized these targets by an approach that allowed us to use a common, already trimerized precursor, tris[3‐bromo‐5‐(methoxycarbonyl)phenyl]triazine **9**, for all derivatives (**4**, **6**, and **8**) with an inner ester substituent. Having observed previously that the triflic‐acid‐catalyzed trimerization of 3‐alkoxycarbonyl‐benzonitriles is efficient even in the presence of methoxycarbonyl substituents, though not with longer alkoxycarbonyl substituents [31], we trimerized the commercially available nitrile **10**, whose reported synthesis relies on the intriguingly selective monosaponification of dimethyl 5‐bromo‐isophthalate **11** to the corresponding monoester **12** [32] (Scheme 3).

Albeit **9** was poorly soluble in the solvent mixture that we used, triple Suzuki coupling with the appropriate boronic acid gave satisfactory yields of the methyl esters **4**_**Me**_, **6**_**Me**_, and **8**_**Me**_. Likewise, we obtained **5**_**Me**_ and **7**_**Me**_ from commercially available *sym*‐tris(3‐bromophenyl)‐triazine **13** and the appropriate methyl‐ester‐bearing boronic acid. From these five methyl esters **4**_**Me**_ **–8**_**Me**_, we accessed the five ethyl homologs **4**_**Et**_ **–8**_**Et**_ by saponification and basic reesterification with bromoethane [33].

**FIGURE 1:**
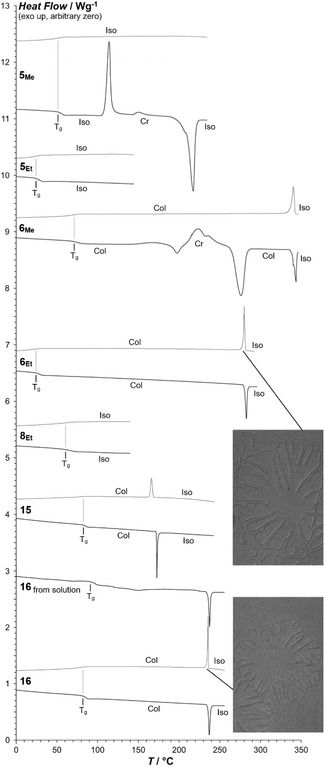
DSC scans (after heating above *T*_m_ except where indicated) at 10°C/min showing the glass transitions of **5**_**Me**_, **5**_**Et**_, **6**_**Me**_, **6**_**Et**_, **8**_**Et**_, **15**, and **16** (cooling scans in gray and heating scans in black). Insets: homeotropic domains of the columnar mesophases of **6**_**Et**_ and **16** growing from the isotropic liquid between glass slides (POM with slightly uncrossed polarizers).

**FIGURE 2:**
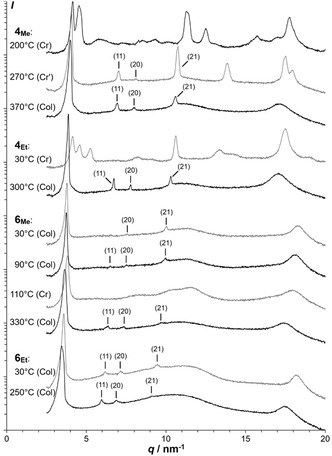
XRD patterns of **4**_**Me**_, **4**_**Et**_, **6**_**Me**_, and **6**_**Et**_ (logarithmic intensity scaling; Miller indices of secondary peaks of the hexagonal lattice in parentheses).

Polarized light optical microscopy (POM) of samples sandwiched between glass slides showed that **4**_**Me**_ and **4**_**Et**_ form an enantiotropic columnar hexagonal mesophase, identifiable by the growth of homeotropic domains when cooling through the isotropic‐to‐LC phase transition. The LC texture remains when cooling to room temperature, but differential scanning calorimetry (DSC) at 10°C/min reveals that the samples crystallize above room temperature, without glass transition. As is usual and expected when increasing the length of the alkyl substituent, *T*_m_ and *T*_cl_ of **4**_**Et**_ are much lower than those of **4**_**Me**_ (Table 1). **4**_**Me**_ displays two crystal modifications at different temperatures (Figure 1), of which the higher‐temperature modification (between the room‐temperature crystal and the high‐temperature mesophase) gives X‐ray diffraction (XRD) peaks at small angles that correspond to distances in the ratio 1:√3:2:√7 as evidence of hexagonal order. Yet, the presence of three supplementary well‐defined peaks at the wide‐angle (right‐hand) part of the diffractogram (Figure 2, second XRD pattern from top, at 14, 17.5, and 18 nm^−1^) corresponding to small distances that are not indexable within a two‐dimensional hexagonal lattice and the absence of a diffuse disk‐to‐disk stacking peak indicate that this phase is not a mesophase but a hexagonal crystal well‐ordered in all three dimensions.

**TABLE 1:** Transitions observed by DSC (10°C/min) on heating and cooling.

Subst., g·mol^−1^	Phase sequence (*T*_onset_ in °C [enthalpy in kJ·mol^−1^])^a^
**4**_**Me**_ (712)	Cr – 272 [7.6] – Cr’ – 303 [23] – Col – 374 [30] – Iso Iso – 374 [28] – Col – 302 [23] – Cr’ – 248 [8.3] – Cr
**4**_**Et**_ (754)	Cr – 229 [29] – Col – 330 [28] – Iso Iso – 328 [26] – Col – 214 [28] – Cr
**5**_**Me**_ (712)	Cr – 209 [48] – Iso *T*_g_ (Iso) = 51 *no phase transition on cooling*
**5**_**Et**_ (754)	Cr – 71 [31] – Iso *T*_g_ (Iso) = 23 *no phase transition on cooling*
**6**_**Me**_ (886)	Cr – 265 [53] – Col – 341 [23] – Iso *T*_g_ (Col) = 69 Iso – 341 [23] – Col
**6**_**Et**_ (970)	Cr – 79 [9.2] – Col – 280 [8.8] – Iso *T*_g_ (Col) = 25 Iso – 280 [9.0] – Col
**7**_**Me**_ (886)	Cr – 355 [32] – Iso Iso – 336 [30] – Cr
**7**_**Et**_ (970)	Cr – 286 [58] – Iso Iso – 277 [58] – Cr
**8**_**Me**_ (1060)	Cr – 343 [77] (Col – 307 [n.d.]) – Iso Iso – 307 [56] – Cr
**8**_**Et**_ (1186)	Cr – 130 [27] – Iso *T*_g_ (Iso) = 59 *no phase transition on cooling*
**14** (754)	Cr – 127 [21, 22] – Col – 328 [24, 25] – Iso Iso – 328 [21, 22] – Col – 119 [9.1] – Cr
**15** (880)	Cr – 244 [40] (Col – 172 [4.9]) – Iso *T*_g_ (Col) = 83 Iso – 169 [4.8] – Col
**16** (940)	Cr – *c.*140 [n.d.] – Col – 236 [7.0] – Iso *T*_g_ (Col) = 82 Iso – 236 [6.9] – Col

a Cr = crystal, Cr’ = hexagonal crystal, Col = hexagonal columnar liquid crystal, Iso = isotropic liquid. Monotropic mesophase in parentheses (**8**_**Me**_: n.d. = enthalpy not determined due to concurrent crystallization; **16**: n.d. = broad, ill‐defined peak at first heating that did not reappear at subsequent scans). In line with convention [34], *T*_g_ was determined as the onset temperature of the calorimetric glass transition step on heating with 10°C/min.

**5**_**Me**_ and **5**_**Et**_, where the methyl ester substituent is in the outer instead of the inner benzene rings, show no mesophase, but after initial melting of the crystalline state, a glass transition in the isotropic liquid phase is observed by DSC on cooling and on reheating. The melting points of **5** are much lower than of **4**. Whereas **5**_**Me**_ crystallizes above the glass transition on reheating at 10°C/min, no such reappearance of the crystalline state on reheating is observed with **5**_**Et**_. Not only *T*_m_ is much lower in **5**_**Et**_ than in **5**_**Me**_, but also *T*_g_ drops considerably, to about room temperature.

**6**_**Me**_ and **6**_**Et**_, where both the inner and the outer benzene rings bear one ester substituent, form a columnar hexagonal mesophase on heating. In contrast to **4** (with only inner ester substituent), a glass transition in the mesophase is observed on cooling and on reheating. **6**_**Me**_ crystallizes on reheating above *T*_g_, but not **6**_**Et**_. Again, *T*_g_ drops to about room temperature from **6**_**Me**_ to **6**_**Et**_.

**7**_**Me**_ and **7**_**Et**_, with two symmetrically placed ester substituents in the outer benzenes and none in the inner, melt at much higher temperatures than **6**_**Me**_ and **6**_**Et**_, show no mesophase, and crystallize easily.

**8**_**Me**_ and **8**_**Et**_, with two symmetrically placed ester substituents in the outer benzenes plus one in the inner, behave very different from each other: **8**_**Me**_, after melting at very high temperature, can be observed by POM to form a monotropic columnar mesophase on cooling, but the transition to the mesophase cannot be identified by DSC due to concurrent crystallization. **8**_**Et**_, on the other hand, melts at much lower temperature, forms no mesophase, and rigidifies to an isotropic glass well above room temperature, with a *T*_g_ that is much higher than in the other glass‐forming ethyl homologs.

Together, the thermal behaviors of **4**_**Me**_ to **8**_**Et**_ taught us the following:

1. The ester substituent on the inner benzene rings is essential for the formation of a columnar mesophase—thus **4**, **6**, and **8**_**Me**_ form a mesophase, but **5** and **7** do not. (The absence of any mesophase in **8**_**Et**_ may mean that two big substituents on the outer benzenes are too voluminous for a flat enough shape). The inner ester substituents induce a better disk shape of the molecule by filling the room between the biphenyl arms and possibly also contribute essential polar disk‐to‐disk interactions close to the molecular centers. These inner ester positions are identical to those that exclusively lead to mesomorphism in triphenyltriazine‐triesters [31].
2. Dissymmetric substitution on the outer benzene rings hinders crystallization enough to allow vitrification—thus **5** and **6** show a glass transition, whereas **4** and **7** do not and crystallize easily. (**8** with its high number of polar ester groups is a limit case, as **8**_**Et**_ forms a glass even though the outer benzenes are symmetrically substituted, whereas the less flexible methyl ester groups in **8**_**Me**_ give easy crystallization).
3. The glass transition temperature drops a lot when replacing methyl by ethyl—whereas **5**_**Et**_ and **6**_**Et**_ have their *T*_g_ close to room temperature, *T*_g_ of **5**_**Me**_ and **6**_**Me**_ is much higher. (Nonmesogenic **8**_**Et**_ has a *T*_g_ well above room temperature, so it is infortunate that mesogenic **8**_**Me**_ crystallizes easily).
4. If **3** is only substituted with equal *meta*‐ester groups and only identical substitution on the three biphenyl arms is considered, a mesogen with a high *T*_g_ can only be obtained at the expense of a very high clearing temperature (i.e., **6**_**Me**_ with *T*_g_ = 69°C and *T*_cl_ = 341°C).

To lower the clearing and melting temperatures while maintaining a high *T*_g_, we then introduced *meta*‐substituents other than ester on the outer benzene rings. The three most straightforward ones, where the required *meta*‐substituted phenylboronic acids are commercially available, are methyl, *tert*‐butyl, and phenyl. Their threefold reaction with **9** gave us the corresponding targets **14**, **15**, and **16** in > 50% yields.

**FIGURE 3:**
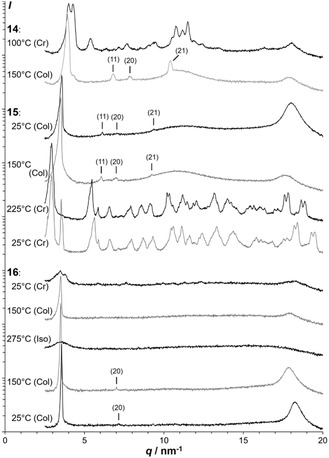
XRD patterns of **14**, **15**, and **16**.

The moderate dissymmetrization from **4**_**Me**_ to **14** by introduction of methyl substituents lowered *T*_m_ by more than 100°C to 127°C but did not affect much *T*_cl_ which remained well above 300°C (Table 1). The much larger *tert*‐butyl substituent in **15** on the other hand gave a much higher (yet still rather moderate) *T*_m_ of 244°C and a much lower *T*_cl_ of 172°C, making the mesophase monotropic (*T*_cl_ < *T*_m_). In contrast to **14** and **4**_**Me**_, the mesophase of **15** could be supercooled (at a moderate cooling speed of −10°C/min) to form a mesomorphic columnar glass with a satisfactorily high *T*_g_ of 83°C. A similar *T*_g_ of 82°C was obtained with **16** with a phenyl substituent, and **16** showed strong suppression of crystallization: A sample obtained by precipitation from solution showed a glass transition step in the DSC scan on first heating, plus a weak and ill‐defined melting peak at *c*.140°C. This indicates that crystallization of **16** from solution is only partial and that the sample precipitates partially as a glass. XRD of an as‐precipitated sample confirms the presence of crystalline domains that disappear at about 140°C (Figure 3). Thereafter, no crystallization could be observed at any temperature.

The column‐to‐column and disk‐to‐disk distances obtained from the XRD patterns of the columnar mesophases allowed to obtain the densities of the mesogenic homologs (Table 2). The densities in the mesophase of about 1.3 g/cm^3^ at room temperature unsurprisingly decreased with increasing temperature, and their values at given temperature were quite similar for all compounds, with one outlier: The density of **15**, with t‐butyl substituents on the outer rings, was found to be notably lower (about 10%) than those of the others. This lower density is not caused by any markedly bigger disk‐to‐disk stacking distance, which is quite uniform across the homologs at given temperature. As **6**_**Me**_ and **15** have near‐identical molecular weight and the same number of nonhydrogen atoms, their column‐to‐column distances can be meaningfully compared: This shows that the decreased density of **15** is caused not by a looser disk‐to‐disk stacking, but by a ca. 5% greater column‐to‐column distance, that is, weaker column interdigitation, hindered by the globular and apolar t‐butyl groups.

**TABLE 2:** Lattice and *π* *–π* stacking reflections obtained by XRD and lattice parameters *a* (i.e., column‐to‐column distances), as well as the resulting density *ρ*, in the hexagonal columnar mesophases.

Subst. (*m* in Da)	*T* in °C	*d*(10) in Å	*d*(11) in Å^a^	*d*(20) in Å^a^	*d*(21) in Å^a^	*a* in Å^b^	*d*_*π* *–π*_ in Å	*ρ* in g·cm^−3^ c
**4**_**Me**_ (712)	370	15.71	9.02 (9.07)	7.79 (7.86)	5.90 (5.94)	18.1	3.65	1.14
**4**_**Et**_ (754)	300	16.24	9.32 (9.38)	8.06 (8.12)	6.09 (6.14)	18.8	3.66	1.12
**6**_**Me**_ (886)	30	16.60		8.29 (8.30)	6.23 (6.27)	19.2	3.47	1.33
	90	16.76	9.66 (9.68)	8.27 (8.38)	6.29 (6.33)	19.4	3.49	1.30
	330	17.20	9.82 (9.93)	8.46 (8.60)	6.45 (6.50)	19.9	3.60	1.20
**6**_**Et**_ (970)	30	17.54	10.16 (10.13)	8.72 (8.77)	6.64 (6.63)	20.3	3.44	1.32
	250	18.20	10.55 (10.51)	9.05 (9.10)	6.92 (6.88)	21.0	3.58	1.18
**14** (754)	150	16.10	9.19 (9.29)	7.95 (8.05)	6.02 (6.08)	18.6	3.49	1.20
**15** (880)	25	17.60	10.22 (10.16)	8.88 (8.80)	6.70 (6.65)	20.3	3.48	1.18
	150	17.89	10.38 (10.33)	8.99 (8.95)	6.79 (6.76)	20.7	3.55	1.12
**16** (940)	25	17.66		8.77 (8.83)		20.4	3.44	1.26
	150	17.96		8.99 (8.98)		20.7	3.51	1.20

a The value in parentheses is calculated, for comparison, from *d*(10)·n^−0.5^ with *n* = 3, 4, or 7.

b Calculated from *d*(10)·sin(60°)^−1^.

c Calculated from *m*·*a* ^−2^·*d*_*π* *–π*_^−1^·sin(60°)^−1^ assuming one molecule per disk. This assumption is a priori reasonable because the estimated diameter of unsubstituted T2T **3** of 19 Å (defined as double the distance from triazine center to outermost hydrogen) is similar to the lattice parameters found, and it is a posteriori reasonable because the obtained densities are as expected slightly above 1 g·cm^−3^. See also the following discussion of interdigitation.

**FIGURE 4:**
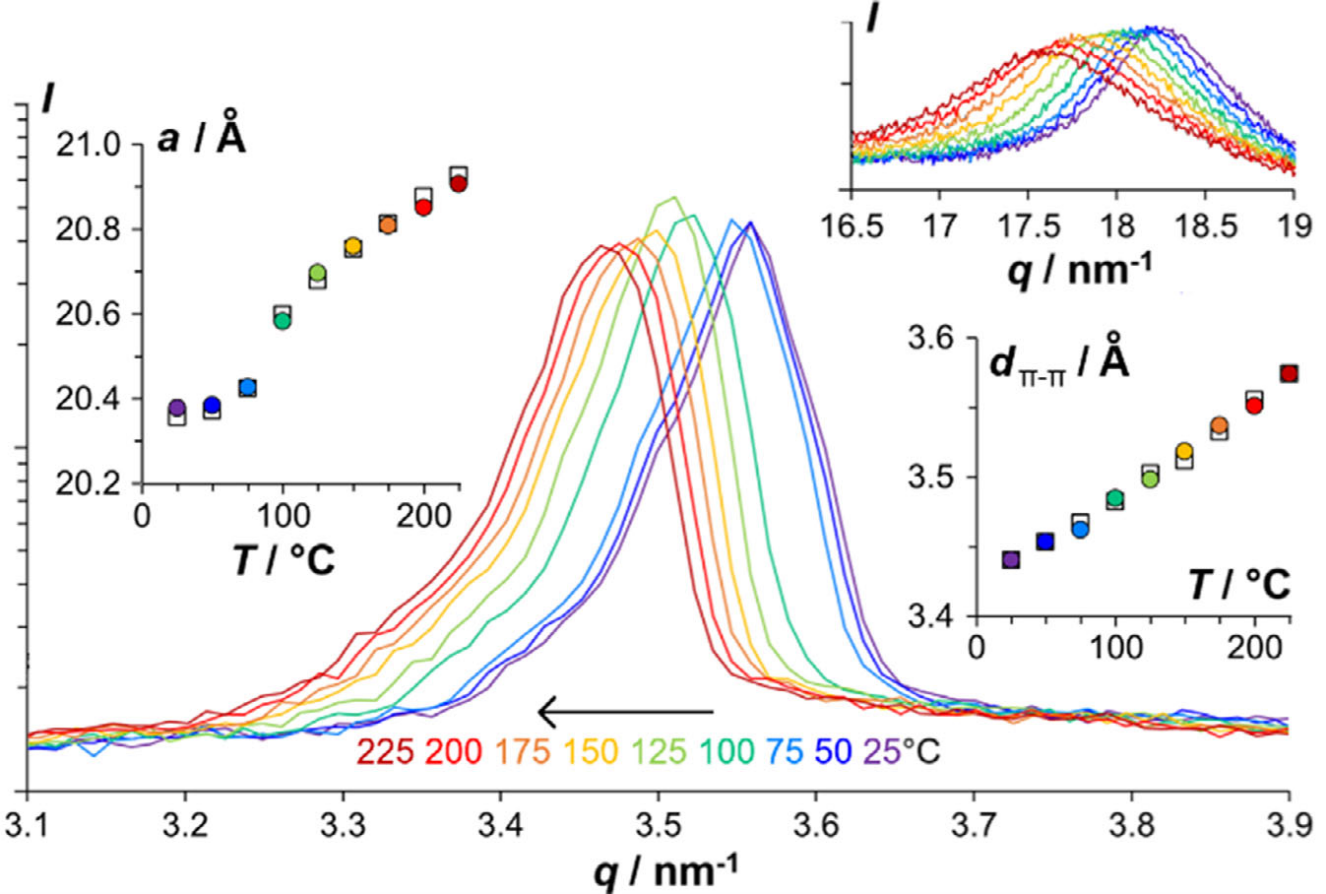
XRD patterns of the main column lattice peak of **16** at different temperatures on heating. Inset left: evolution of the lattice parameter *a* (i.e., column‐to‐column distance) with temperature. Insets right: disk stacking peak at same temperatures as main graph, on heating, and, below, evolution of the disk stacking distance with temperature. Colored circles, on heating; white squares, on cooling.

The resilience of **16** against crystallization allowed the recording of XRD spectra at various temperatures in the mesophase above and below the glass transition, to probe the evolution of the column‐to‐column and disk‐to‐disk distances with temperature on both sides of the glass transition. While the disk‐to‐disk stacking peak, that is, the XRD signal representing the fluid dimension of the system, does not show any discontinuity around *T*_g_ in its slight temperature dependence, the column lattice peak shows a discontinuity at the glass transition: On cooling, the slight shrinking of the column‐to‐column distances with temperature suddenly becomes more prominent at the glass transition and then remains at a near‐constant distance on further cooling. Likewise on heating, the lattice parameter does not expand much well below *T*_g_ and then increases pronouncedly around *T*_g_ and returns to moderate increases per degree at higher temperature (Figure 4). We observed the same intriguing behavior with **6**_**Me**_ and **15**.

Thus, the density change at the glass transition followed by a decrease of the temperature dependence well below *T*_g_ is selectively observed in the dimensions of the column lattice, but not in the a priori less periodic stacking dimension. This may tentatively be explained by a greater interdigitation with the neighboring molecules in the adjacent columns than with the neighboring molecules along the stack. The lattice parameter (column‐to‐column distance) of **16** at *T*_g_ is ca. 20.5 Å, whereas in extended conformation, the distance from the center of the molecule to the end of a terphenyl arm is ca. 12 Å, that is, significantly longer than half the column‐to‐column distance. This column‐to‐column interdigitation is influenced by the conformational flexibility of the molecular arms. Freezing of their neighbor‐repelling conformational flipping at *T*_g_ may allow an initial densification, followed at further cooling by a lock‐in of the so‐obtained distance.

**FIGURE 5:**
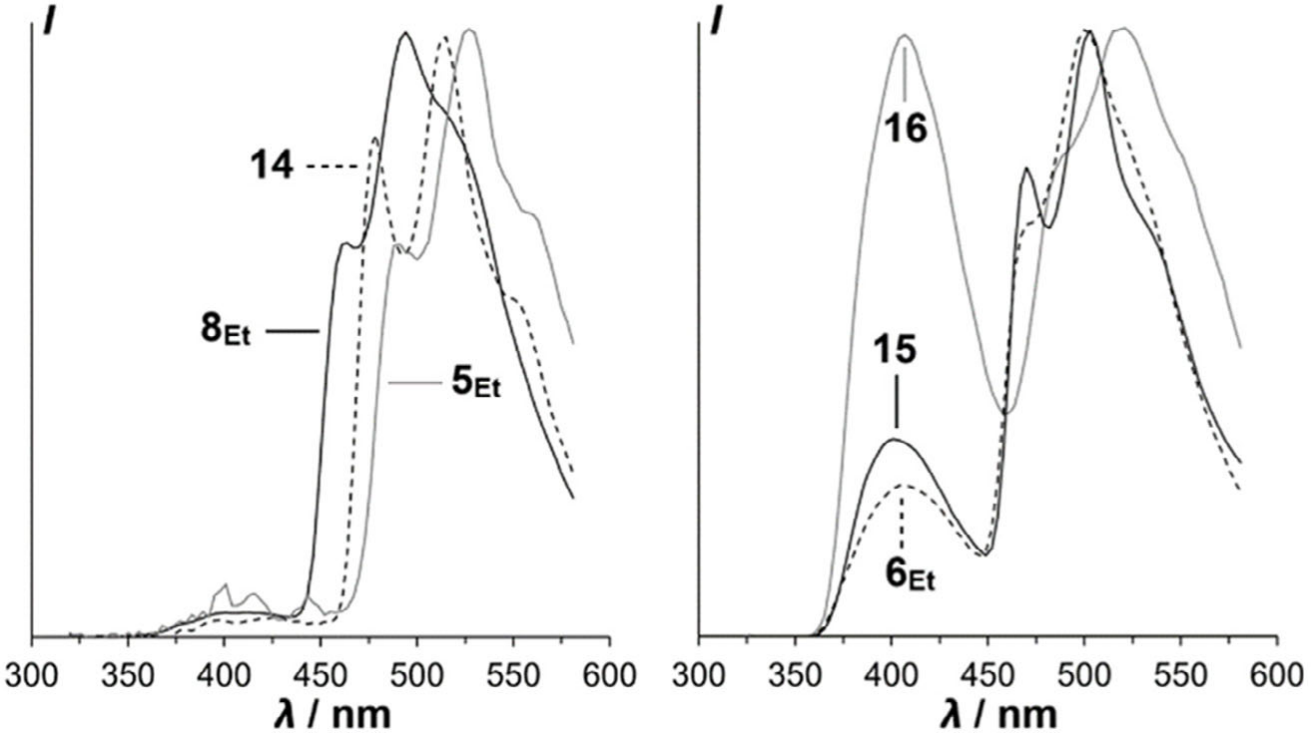
Normalized delayed luminescence spectra under vacuum at 60 K of films spin‐coated from chloroform solution, recorded with a delay of 0.1 ms after illumination at 300 nm. Left: **5**_**Et**_, **8**_**Et**_, and **14** that do not form a columnar glass. Right: **6**_**Et**_, **15**, and **16** that form columnar glasses; vertical lines point to DF peaks. (**4**_**Et**_ and **7**_**Et**_ phosphoresced only negligibly).

To determine the *T*_1_–*S*_0_ energy gap in the solid materials, we measured the delayed luminescence of spin‐coated condensed solid films of **4**_**Et**_, **5**_**Et**_, **6**_**Et**_, **7**_**Et**_, **8**_**Et**_, **14**, **15**, and **16** with a delay of 0.1 ms after illumination at 60 K (Figure 5). While **4**_**Et**_ and **7**_**Et**_ gave no significant phosphorescence signals, the other six materials showed not only phosphorescence, but also DF. The presence of this DF at the low temperature of 60 K, together with a large energy difference of about 0.65 eV between the onsets of fluorescence (ca. 370 nm [3.35 eV], associated with *S*_1_) and phosphorescence (around 460 nm [2.7 eV], associated with *T*_1_), precludes the possibility of TADF which relies on thermal activation and very small *S*_1_–*T*_1_ differences. This implies that the DF stems from triplet–triplet annihilation (TTA), as previously observed in the columnar mesophase of a blend of two columnar mesogens [35].

**FIGURE 6:**
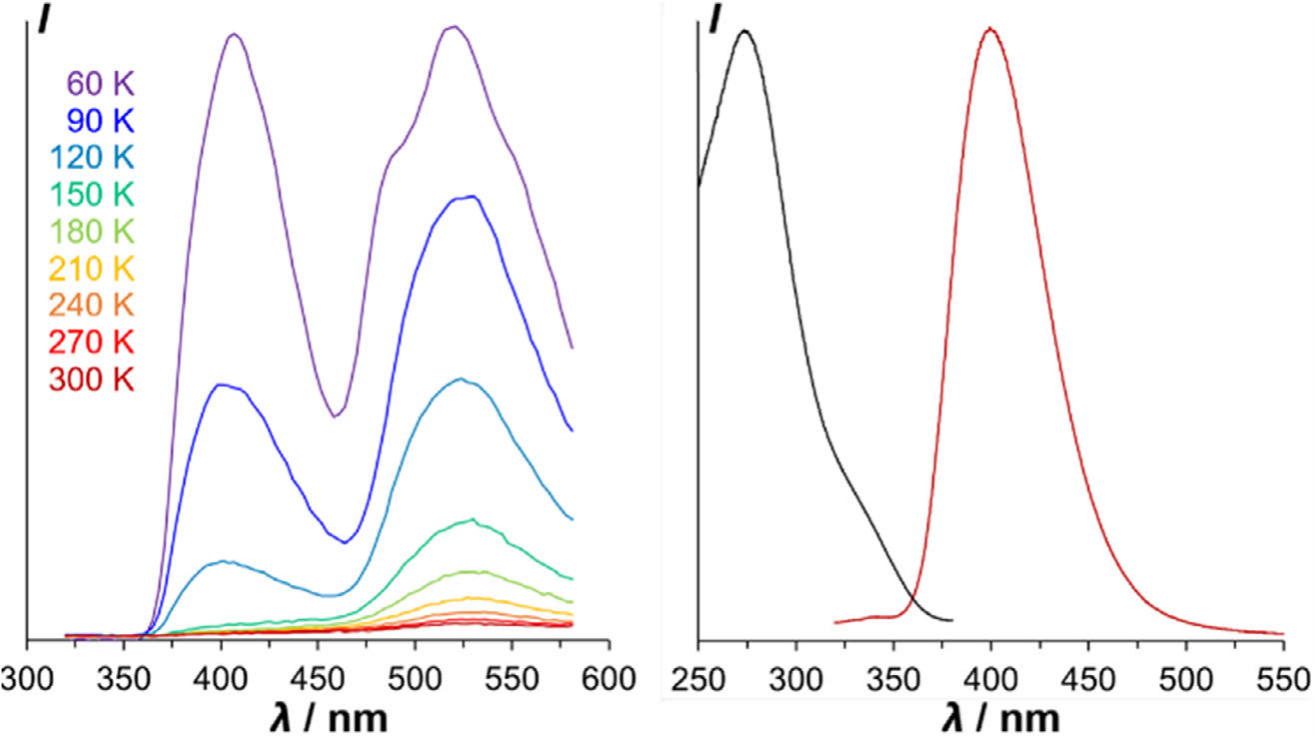
Left: delayed luminescence spectra of **16** at different temperatures under vacuum; spin‐coated on glass from chloroform solution, recorded with a delay of 0.1 ms after illumination at 300 nm. Right: steady‐state emission of spin‐coated film of **16** at room temperature under illumination at 300 nm (red) and excitation spectrum of emission at 400 nm (black).

We measured the relative intensities of delayed emission of **16** at different temperatures in thin film between 60 and 300 K and found that both DF at around 400 nm and phosphorescence at 470–580 nm decrease with increasing temperature and that the DF decreases faster than the phosphorescence (Figure 6, left). Above 150 K, no significant DF remains. Phosphorescence is discernible up to about room temperature, where no significant long‐wavelength emission persists (Figure 6, right). The observed decrease of DF with increasing temperature is not only incompatible with TADF, but it is also in line with previously reported TTA‐DF from organic emitters such as MEH‐PPV [36, 37].

This DF is very weak in the materials that either crystallize easily (**14**) or tend to form isotropic glasses (**5**_**Et**_ and **8**_**Et**_) and much more pronounced in those that tend to form hexagonal columnar mesomorphic glasses (**6**_**Et**_, **15**, and **16**). The low‐temperature DF from **16** is particularly strong.

TTA requires the close proximity of the *π* electron clouds of pairs of adjacent molecules. This proximity is best given in columnar stacks. It is thus not surprising to see much more TTA‐DF from columnar than from other homologs. TTA may be most efficient in tris(terphenylyl)triazine **16** because it has a larger aromatic system than the tris(biphenylyl)triazines **6**_**Et**_ and **15**. Thus adjacent molecules in stacked **16** may have more *π* contact than is feasible in stacks of tris(biphenylyl)triazines. Measuring the relative intensities of the DF in different noncrystalline states of matter in a same compound at different temperatures, from room temperature upward to the high temperatures of the isotropic phase, to illustrate the evolution of DF through the glass and clearing transitions (i.e., in the columnar glass, in the fluid columnar LC, and in the liquid) is currently beyond our experimental possibilities but remains a fascinating challenge for the future.

From the short‐wavelength onset of the solid‐film phosphorescence, we estimated the energy of the first excited triplet state *T*_1_ with respect to the singlet ground state *S*_0_. The obtained *T*_1_ energies for the six measured materials varied from 2.64 to 2.79 eV (445 to 470 nm) (Table 3). For parent arene **3** (T2T), a *T*_1_ value of 2.8 eV has been reported from the shortest‐wavelength phosphorescence maximum *in solution* in solid ethanol at 77 K [28]. The slightly smaller *T*_1_ values found in the condensed solid state of the esters are likely to be attributed in part to intermolecular interactions in the undiluted solid rather than to the substitution pattern of the isolated molecule: The material with the highest number of ester substituents (**8**_**Et**_) has the highest *T*_1_ energy, whereas the only material that has no ester substituent on the inner benzene rings (**5**_**Et**_) has the lowest *T*_1_ energy, even lower than the material with the larger arene system (**16**). Thus, the inner ester substituents that appear to be an essential feature for mesophase formation have no discernible detrimental effect on *T*_1_. A columnar mesophase structure in the film has no discernible negative influence on *T*_1_: Between the two electronically most similar materials **14** and **15**, the latter, which forms a columnar glass, has a higher *T*_1_ energy than the former, which easily crystallizes. Yet caution dictates to note that the small overall variation of the *T*_1_ values by only about 0.15 eV makes the identification of clear structure‐ or packing‐related trends haphazardous. Overall, the obtained solid‐state *T*_1_ values are at the low end of what is required for a host material for blue triplet emitters, and further optimization to get the solid‐state *T*_1_ energy closer to 3 eV remains valuable.

**TABLE 3:** Short‐wavelength onset of phosphorescence, with corresponding *T*_1_ *–S*_0_ gap energy, under vacuum at 60 K in films spin‐coated from chloroform solution. **4_Et_** and **7_Et_** are not listed as they did not show significant delayed emission.

Substance	*λ*_onset_ of phosphorescence, nm	*T*_1_ energy, eV
**5_Et_**	470	2.64
**6_Et_**	450	2.76
**8_Et_**	445	2.79
**14**	464	2.67
**15**	454	2.73
**16**	457	2.71

## 3 Conclusion

Conformationally flexible ester‐substituted derivatives of *sym*‐tris(biphenyl‐3‐yl)triazine **3** are simply accessible by combinations of Suzuki couplings and nitrile trimerizations.

Ester substituents in the *meta* positions of the inner benzene moieties enable columnar mesomorphism with a variety of substituents on the outer benzenes.

Methyl esters form mesomorphic or isotropic glasses at elevated temperatures, with much higher *T*_g_ than ethyl esters. The sensitivity of the column lattice spacing to the glass transition is much greater than the sensitivity of the disk‐to‐disk spacing.

The combination of *meta*‐linked benzene and triazine moieties decorated with *meta*‐ester substituents gives rather high *T*_1_ energies. Mesomorphic columnar stacking favors the TTA that leads to delayed fluorescence: The close and rather uniform intermolecular proximity of the arene systems within the columnar stacks facilitates triplet movement and exchange over increased distances compared to isotropic glasses, whose molecular packing is globally random and ordered only very locally, if at all.

## 4 Experimental Section

### 4.1 Synthesis of sym‐Tris[3‐bromo‐5‐(methoxycarbonyl)phenyl]triazine 9

Trifluoromethanesulfonic acid (10 mL) was added to 3‐bromo‐5‐(methoxycarbonyl)benzonitrile **10** [32] (9.6 g, 40 mmol). The mixture, which quickly became a homogeneous viscous solution, was stirred for 3 days and then poured into vigorously stirred water (200 mL), whereupon a precipitate formed. This was filtered off on a glass frit and washed with water, then methanol, and let dry to yield **9** (8.1 g, 84%) as a white solid, which was used in the next step without further purification.

### 4.2 General Procedure for the Synthesis of Methyl Esters 4_Me_–8_Me_ and 14–16

Either **9** or *sym*‐tris(3‐bromo‐phenyl)triazine **13** (1.67 mmol), the appropriate substituted phenylboronic acid (7.0 mmol), potassium carbonate (1.10 g, 8.0 mmol), and tetrakis(triphenylphosphine)‐palladium (0.125 g, 0.11 mmol) were stirred at reflux in a mixture of toluene (60 mL) and methanol (30 mL) under argon for 5 h.

### 4.3 Isolation of Triesters 4_Me_, 5_Me_, 14, 15, and 16

To the reaction mixture was added aqueous 1 M hydrochloric acid (200 mL) and hot chloroform (500 mL), and the biphasic mixture was refluxed until nearly all insolubles had dissolved. The organic phase was filtered hot and concentrated to dryness. The residue was dissolved by refluxing in chloroform and the hot solution was passed through a column of silica, and elution was completed with chloroform. The solution was concentrated to 100 mL and the product was precipitated by adding methanol.

### 4.4 Isolation of Hexaesters 6_Me_ and 7_Me_ and Nonaester 8_Me_, Which Are Poorly Soluble in Organic Solvents Except in Near‐Boiling 1,1,2,2‐Tetrachloroethane

To the reaction mixture was added aqueous 1 M hydrochloric acid (200 mL), hot chloroform (500 mL) was added with stirring, and the biphasic mixture was filtered over a glass frit to separate remaining insolubles (which are largely made up of product). The organic phase was concentrated and the residue together with the filtered‐off insolubles was dissolved in hot tetrachloroethane (50 mL) (caution, toxic!). This hot solution was passed through a short column of silica, and elution was completed with either hot chloroform containing 5% of acetone (for **6**_**Me**_ or **7**_**Me**_) or hot tetrachloroethane containing 5% of acetone (for **8**_**Me**_, which was the least soluble of the three). The solution was concentrated to 100 mL, and precipitation of the product was completed by adding methanol.

Yields: **4**_**Me**_, 87%; **5**_**Me**_, 54%; **6**_**Me**_, 69%; **7**_**Me**_, 88%; **8**_**Me**_, 61%; **14**, 88%; **15**, 75%; **16**, 60%.

### 4.5 General Procedure for the Synthesis of Ethyl Esters 4_Et_–8_Et_

The corresponding methyl ester (1.2 mmol) and potassium hydroxide (4.5 g, 80 mmol) were stirred at reflux for 2 days in a mixture of water (150 mL) and ethanol (50 mL). The mixture was concentrated to 100 mL to eliminate most of the ethanol, topped up with water (150 mL), and filtered hot to remove a small amount of insoluble impurities. An excess of concentrated hydrochloric acid was added to precipitate the tri‐ or hexacarboxylic acid, which was filtered off and air‐dried. The obtained solid was stirred at reflux in ethanol (30 mL) with bromoethane (30 mL, 0.4 mol) and 1,8‐diazabicyclo[5.4.0]undec‐7‐ene (7.5 mL, 50 mmol) for 20 h. The mixture was added to aqueous 1 M hydrochloric acid (200 mL), extracted with chloroform, and concentrated. The residue was purified by chromatography in chloroform:acetone 19:1 on silica and recrystallization from butanol.

Yields: **4**_**Et**_, 49%; **5**_**Et**_, 96%; **6**_**Et**_, 84%; **7**_**Et**_, 77%; **8**_**Et**_, 53%.

## Supporting Information

Additional supporting information can be found online in the Supporting Information section.

## Conflicts of Interest

The authors declare no conflicts of interest.

## Supporting information

Supplementary Material

## Data Availability

The data that support the findings of this study are available from the corresponding author upon reasonable request.
