# Supplementary material for: Lattice Stiffening and Delayed Fluorescence in Columnar Liquid‐Crystalline Glasses
Source: Chempluschem. 2026 Mar 22;91(3):e202500738. doi: 10.1002/cplu.202500738 (PMC13006150; doi:10.1002/cplu.202500738)
Supplement: Supplementary file 1 — Supplementary Material [file CPLU-91-e202500738-s001.pdf]

Ester-substituted tris(biphenylyl)triazines: Columnar liquid-crystalline glasses with delayed fluorescence

Monike da Silva Kutz, Wilson Aparecido de Oliveira, Marilia Gabriela Belarmino Cabral, Fabien Durola, Ivan H. Bechtold, Eduard Westphal, and Harald Bock

Supporting Information

Luminescence measurements

The luminescence spectra of the thin films were recorded using a Horiba FluoroMax Plus spectrophotometer. The films were analyzed under vacuum at different temperatures using a Janis CCS- 400/202 closed-cycle refrigerator system, coupled to a LakeShore 331 temperature controller and a Sumitomo cryogenics HC-4e indoor water-cooled compressor. Spin-coated films were fabricated by depositing the solution (10 mg/mL in chloroform) onto quartz substrates and subsequently spin coating at 2000 rpm during 30s.

X-ray diffraction data acquisition

The X-Ray diffraction experiments were realized with the X`Pert-PRO (PANalytical) diffractometer system using the linear monochromatic CuKα1 beam (λ = 1.5405 Å), with an applied power of 1.2kVA. The scans were performed in continuous mode from 2° to 30° (2θ angle) and the diffracted radiation collected with the X'Celerator detector. Compounds were placed on the TCU2000 - Temperature Control Unit (Anton Paar), which allows a precise control of the sample temperature during the measurement.

Compound characterization data

2,4,6-tris-(5-(methoxycarbonyl)-biphenyl-3-yl)-1,3,5-triazine 4_{Me}:
^{1}H-NMR ($CDCl_2CDCl_2$, 400 MHz): δ = 9.23 (t, 2Hz, 3H), 9.11 (t, 2Hz, 3H), 8.44 (t, 2Hz, 3H), 7.75 (d, 8Hz, 6H), 7.52 (t, 8Hz, 6H), 7.45 (t, 8Hz, 3H), 3.97 (s, 9H) ppm.
^{13}C-NMR ($CDCl_2CDCl_2$, 100 MHz): δ = 171.3, 166.6, 142.0, 139.4, 136.8, 132.3, 131.8, 131.5, 129.3, 129.0, 128.5, 127.4, 52.8 ppm.
ESI-HRMS: *m/z* calcd for $C_{45}H_{34}N_3O_6$ [*MH*]+: 712.2442; found: 712.2460.

2,4,6-tris(5-(ethoxycarbonyl)-biphenyl-3-yl)-1,3,5-triazine 4_{Et}:
^{1}H-NMR ($CDCl_3$, 400 MHz): δ = 9.39 (t, 2Hz, 3H), 9.21 (t, 2Hz, 3H), 8.54 (t, 2Hz, 3H), 7.78 (d, 8Hz, 6H), 7.53 (t, 8Hz, 6H), 7.45 (t, 8Hz, 3H), 4.49 (q, 7Hz, 6H), 1.50 (t, 7Hz, 9H) ppm.
^{13}C-NMR ($CDCl_3$, 100 MHz): δ = 171.1, 166.0, 141.9, 139.5, 136.6, 132.3, 131.8, 131.5, 129.1, 128.8, 128.2, 127.2, 61.4, 14.5 ppm.
ESI-HRMS: *m/z* calcd for $C_{48}H_{39}N_3O_6Na$ [*MNa*]+: 776.2731; found: 776.2745.

2,4,6-tris(3‘-(methoxycarbonyl)-biphenyl-3-yl)-1,3,5-triazine 5$_{Me}$:
^{1}H-NMR ($CDCl_3$, 400 MHz): δ = 8.99 (t, 2Hz, 3H), 8.77 (d, 8Hz, 3H), 8.39 (t, 2Hz, 3H), 8.07 (d, 8Hz, 3H), 7.92 (d, 8Hz, 3H), 7.85 (d, 8Hz, 3H), 7.66 (t, 8Hz, 3H), 7.57 (t, 8Hz, 3H), 3.94 (s, 9H) ppm.
^{13}C-NMR ($CDCl_3$, 100 MHz): δ = 171.7, 167.1, 141.1, 140.7, 136.8, 131.8, 131.5, 130.9, 129.4, 129.1, 128.8, 128.5, 127.7, 52.4 ppm.
Low solubility precluded meaningful HRMS, whereas transformation into the ethyl homologue lead to sufficient solubility for HRMS (see following compound).

2,4,6-tris(3‘-(ethoxycarbonyl)-biphenyl-3-yl)-1,3,5-triazine 5$_{Et}$:
^{1}H-NMR ($CDCl_3$, 400 MHz): δ = 9.00 (t, 2Hz, 3H), 8.78 (d, 8Hz, 3H), 8.40(t, 2Hz, 3H), 8.08 (d, 8Hz, 3H), 7.92 (d, 8Hz, 3H), 7.86 (d, 8Hz, 3H), 7.66 (t, 8Hz, 3H), 7.58 (t, 8Hz, 3H), 4.41 (q, 7Hz, 6H), 1.41 (t, 7Hz, 9H) ppm.
^{13}C-NMR ($CDCl_3$, 100 MHz): δ = 171.7, 166.6, 141.0, 140.8, 136.8, 131.7, 131.5, 131.3, 129.4, 129.1, 128.8, 128.5, 128.4, 127.7, 61.2, 14.5 ppm.
ESI-HRMS: *m/z* calcd for $C_{48}H_{39}N_3O_6Na$ [*MNa*]+: 776.2731; found: 776.2750.

2,4,6-tris(3‘,5-bis(methoxycarbonyl)-biphenyl-3-yl)-1,3,5-triazine 6$_{Me}$:
^{1}H-NMR ($CDCl_2CDCl_2$, 400 MHz): δ = 9.18 (s, 3H), 9.02 (s, 3H), 8.37(s, 3H), 8.29 (s, 3H), 8.02 (d, 8Hz, 3H), 7.87 (d, 8Hz, 3H), 7.53 (t, 8Hz, 3H), 3.96 (s, 9H), 3.89 (s, 9H) ppm.
^{13}C-NMR ($CDCl_2CDCl_2$, 100 MHz): δ = 171.1, 166.9, 166.4, 140.8, 139.4, 136.6, 132.2, 131.7, 131.5, 130.9, 129.4, 129.3, 128.3, 52.9, 52.6 ppm.
Low solubility precluded meaningful HRMS, whereas transformation into the ethyl homologue lead to sufficient solubility for HRMS (see following compound).

2,4,6-tris(3‘,5-bis(ethoxycarbonyl)-biphenyl-3-yl)-1,3,5-triazine 6$_{Et}$:
^{1}H-NMR ($CDCl_3$, 400 MHz): δ = 9.38 (t, 2Hz, 3H), 9.17 (t, 2Hz, 3H), 8.53 (t, 2Hz, 3H), 8.41 (t, 2Hz, 3H), 8.09 (d, 8Hz, 3H), 7.95 (d, 8Hz, 3H), 7.58 (t, 8Hz, 3H), 4.48 (q, 7Hz, 6H), 4.40 (q, 7Hz, 6H), 1.47 (t, 7Hz, 9H), 1.40 (t, 7Hz, 9H) ppm.
^{13}C-NMR ($CDCl_3$, 100 MHz): δ = 171.4, 166.4, 166.0, 141.3, 140.0, 136.9, 132.4, 132.1, 131.73, 131.66, 131.4, 129.4, 129.2, 128.5, 61.6, 61.3, 14.4 ppm.
ESI-HRMS: *m/z* calcd for $C_{57}H_{51}N_3O_{12}Na$ [*MNa*]+: 992.3365; found: 992.3393.

2,4,6-tris(3‘,5‘-bis(methoxycarbonyl)-biphenyl-3-yl)-1,3,5-triazine 7$_{Me}$:
^{1}H-NMR ($CDCl_2CDCl_2$, 400 MHz): δ = 8.84 (s, 3H), 8.70 (d, 8Hz, 3H), 8.49 (s, 3H), 8.40 (s, 6H), 7.75 (d, 8Hz, 3H), 7.61 (t, 8Hz, 3H), 3.83 (s, 18H) ppm.
^{13}C-NMR ($CDCl_2CDCl_2$, 100 MHz): δ = 171.4, 166.1, 141.5, 139.6, 136.6, 132.5, 131.6, 131.1, 129.6, 129.5, 129.0, 127.7, 52.7 ppm.
Low solubility precluded meaningful HRMS, whereas transformation into the ethyl homologue lead to sufficient solubility for HRMS (see following compound).

2,4,6-tris(3‘,5‘-bis(ethoxycarbonyl)-biphenyl-3-yl)-1,3,5-triazine 7$_{Et}$:
^{1}H-NMR ($CDCl_3$, 400 MHz): δ = 8.91 (s, 3H), 8.72 (d, 8Hz, 3H), 8.56 (s, 3H), 8.47 (d, 2Hz, 6H), 7.80 (d, 8Hz, 3H), 7.65 (t, 8Hz, 3H), 4.32 (q, 7Hz, 12H), 1.35 (t, 7Hz, 18H) ppm.
^{13}C-NMR ($CDCl_3$, 100 MHz): δ = 171.5, 165.7, 141.5, 139.9, 136.8, 132.5, 131.5, 131.4, 129.5, 129.3, 128.9, 127.9, 61.5, 14.4 ppm.
ESI-HRMS: *m/z* calcd for $C_{57}H_{51}N_3O_{12}Na$ [*MNa*]+: 992.3365; found: 992.3395.

2,4,6-tris(3‘,5,5‘-tris(methoxycarbonyl)-biphenyl-3-yl)-1,3,5-triazine 8$_{Me}$:
^{1}H-NMR ($CDCl_2CDCl_2$, 125 °C, 400 MHz): δ = 9.41 (s, 3H), 9.20 (s, 3H), 8.69 (s, 3H), 8.58 (d, 2Hz, 6H), 8.55 (s, 3H), 4.08 (s, 9H), 3.98 (s, 18H) ppm.
^{13}C-NMR ($CDCl_2CDCl_2$, 125 °C, 100 MHz): δ = 171.7, 166.2, 165.9, 140.6, 140.5, 137.4, 132.5, 132.4, 132.2, 132.1, 131.7, 130.2, 130.1, 52.44, 52.37 ppm.
Low solubility precluded meaningful HRMS, whereas transformation into the ethyl homologue lead to sufficient solubility for HRMS (see following compound).

2,4,6-tris(3‘,5,5‘-tris(ethoxycarbonyl)-biphenyl-3-yl)-1,3,5-triazine 8_{Et}:
^{1}H-NMR ($CDCl_3$, 400 MHz): δ = 9.38 (s, 3H), 9.07 (s, 3H), 8.60 (s, 3H), 8.51 (s, 6H), 8.48 (s, 3H), 4.49 (q, 7Hz, 6H), 4.34 (q, 7Hz, 12H), 1.46 (t, 7Hz, 9H), 1.36 (t, 7Hz, 18H) ppm.
^{13}C-NMR ($CDCl_3$, 100 MHz): δ = 171.4, 165.8, 165.5, 140.60, 140.55, 137.0, 132.5, 132.2, 131.9, 131.8, 129.9, 129.8, 61.64, 61.60, 14.43, 14.36 ppm.
ESI-HRMS: *m/z* calcd for $C_{66}H_{64}N_3O_{18}$ [*MH*]+: 1186.4179; found: 1186.4162.

2,4,6-tris(3-bromo-5-(methoxycarbonyl)-phenyl)-1,3,5-triazine 9:
^{1}H-NMR ($CDCl_2CDCl_2$, 400 MHz): δ = 8.93 (s, 3H), 8.72 (s, 3H), 8.26 (s, 3H), 4.00 (s, 9H) ppm.
^{13}C-NMR ($CDCl_2CDCl_2$, 100 MHz): δ = 170.1, 165.2, 137.2, 136.8, 135.7, 132.5, 128.6, 123.3, 53.2 ppm.
Low solubility precluded meaningful HRMS of this intermediate.
2,4,6-tris-(3‘-methyl-5-(methoxycarbonyl)-biphenyl-3-yl)-1,3,5-triazine 14:
^{1}H-NMR ($CDCl_3$, 400 MHz): δ = 9.22(s, 3H), 9.07 (s, 3H), 8.42 (s, 3H), 7.52 (br, 6H), 7.37(t, 8Hz, 3H), 7.23 (d, 8Hz, 3H), 3.98 (s, 9H), 2.46 (s, 9H) ppm.
^{13}C-NMR ($CDCl_3$, 100 MHz): δ = 171.1, 166.5, 141.9, 139.3, 138.7, 136.5, 132.1, 131.5, 131.3, 129.0, 128.9, 128.7, 128.0, 124.3, 52.4, 21.7 ppm.
ESI-HRMS: *m/z* calcd for $C_{48}H_{39}N_3O_6Na$ [*MNa*]+: 776.2731; found: 776.2747.

2,4,6-tris-(3‘-(*tert*-butyl)-5-(methoxycarbonyl)-biphenyl-3-yl)-1,3,5-triazine 15:
^{1}H-NMR ($CDCl_3$, 400 MHz): δ = 9.36 (t, 2Hz, 3H), 9.18 (t, 2Hz, 3H), 8.52 (t, 2Hz, 3H), 7.75 (s, 3H), 7.58 (dt, 8Hz, 2Hz, 3H), 7.49 (dt, 8Hz, 2Hz, 3H), 7.46 (t, 8Hz, 3H), 4.03 (s, 9H), 1.42 (s, 27H) ppm.
^{13}C-NMR ($CDCl_3$, 100 MHz): δ = 171.5, 166.8, 152.1, 143.0, 139.5, 136.9, 132.6, 132.1, 131.5, 128.9, 128.8, 125.3, 124.7, 124.5, 52.6, 35.0, 31.6 ppm.
ESI-HRMS: *m/z* calcd for $C_{57}H_{58}N_3O_6$ [*MH*]+: 880.4320; found: 880.4340.

2,4,6-tris-(3‘-phenyl-5-(methoxycarbonyl)-biphenyl-3-yl)-1,3,5-triazine 16:
^{1}H-NMR ($CDCl_3$, 400 MHz): δ = 9.39 (s, 3H), 9.26 (s, 3H), 8.57 (s, 3H), 7.97 (s, 3H), 7.74 (d, 8Hz, 3H), 7.69 (d, 8Hz, 6H), 7.64 (d, 8Hz, 3H), 7.53 (t, 8Hz, 3H), 7.46 (t, 8Hz, 6H), 7.37 (t, 8Hz, 3H), 3.94 (s, 9H) ppm.
^{13}C-NMR ($CDCl_3$, 100 MHz): δ = 171.2, 166.5, 142.2, 142.0, 140.9, 140.1, 136.8, 132.4, 131.8, 131.5, 129.6, 129.1, 129.0, 127.7, 127.4, 127.1, 126.2, 126.1, 52.5 ppm.
ESI-HRMS: *m/z* calcd for $C_{63}H_{45}N_3O_6Na$ [*MNa*]+: 962.3201; found: 962.3231.

NMR spectra

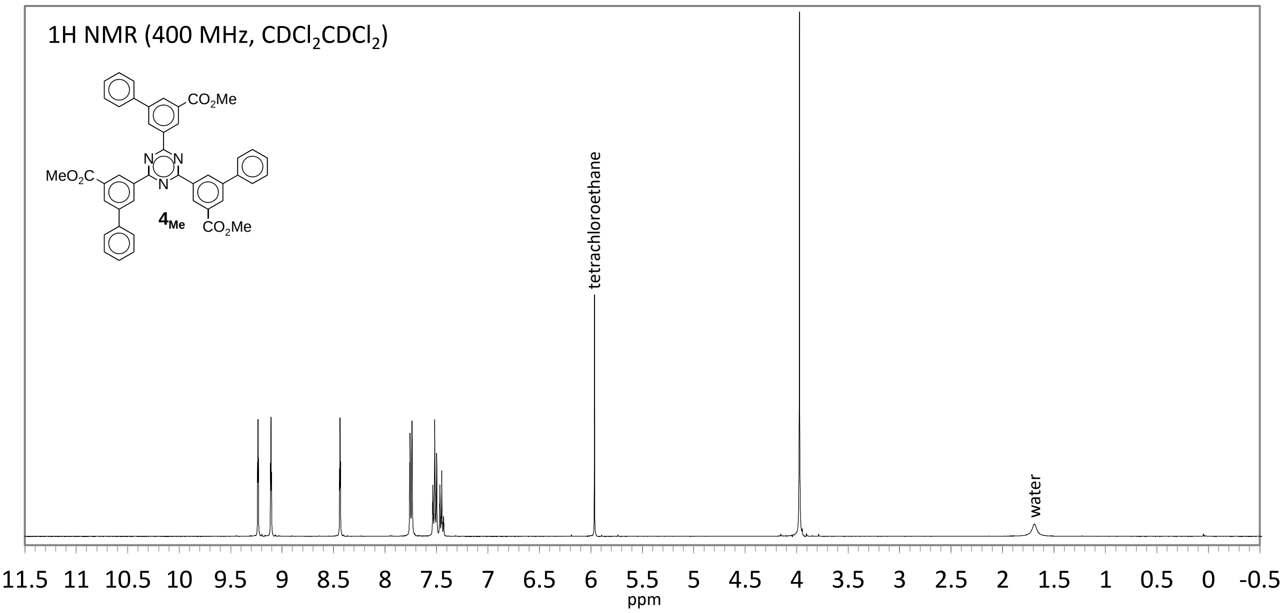

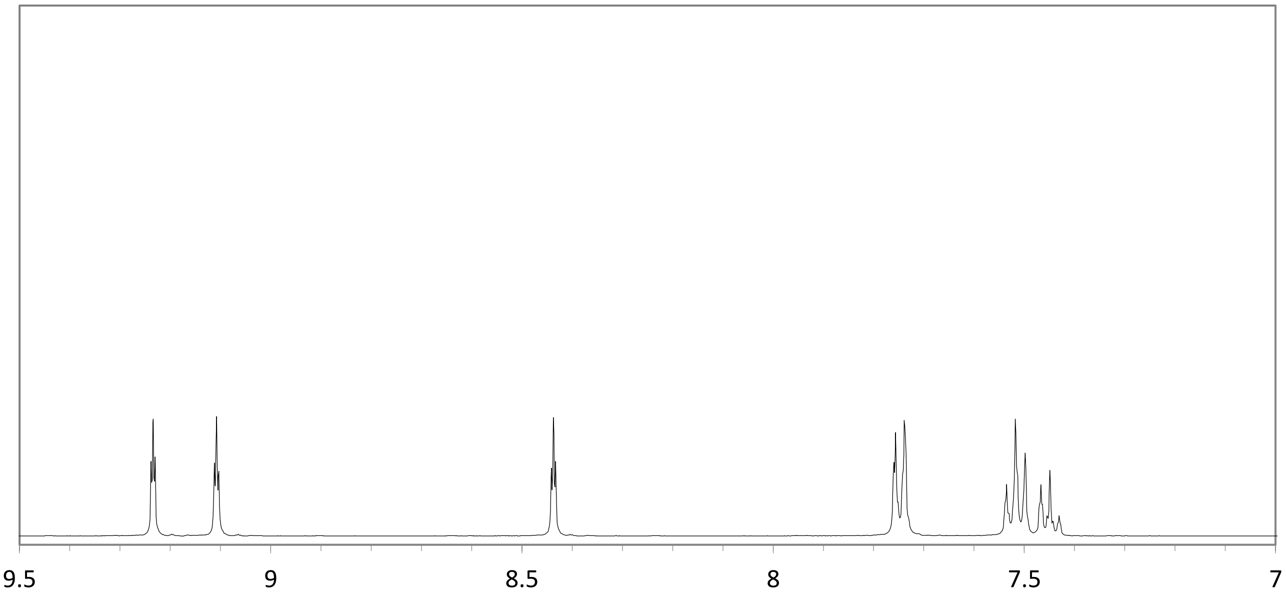

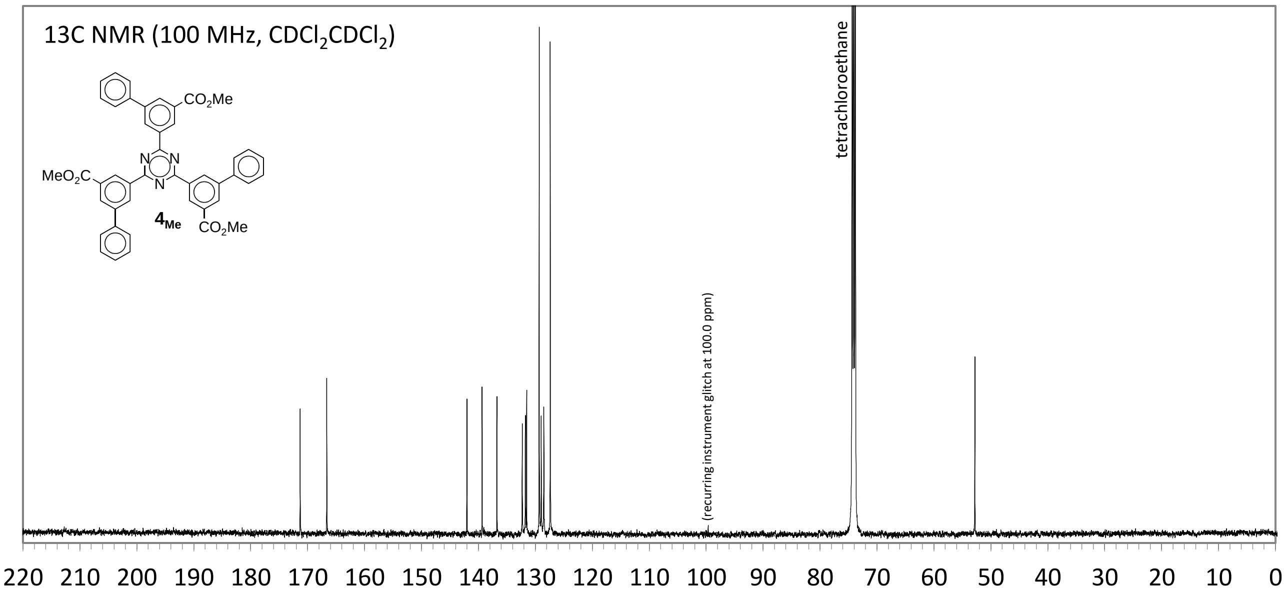
13C NMR (100 MHz, CDCl2CDCl2)
CO2Me
MeO2C
N
4Me
CO2Me
tetrachloroethane
(recurring instrument glitch at 100.0 ppm)

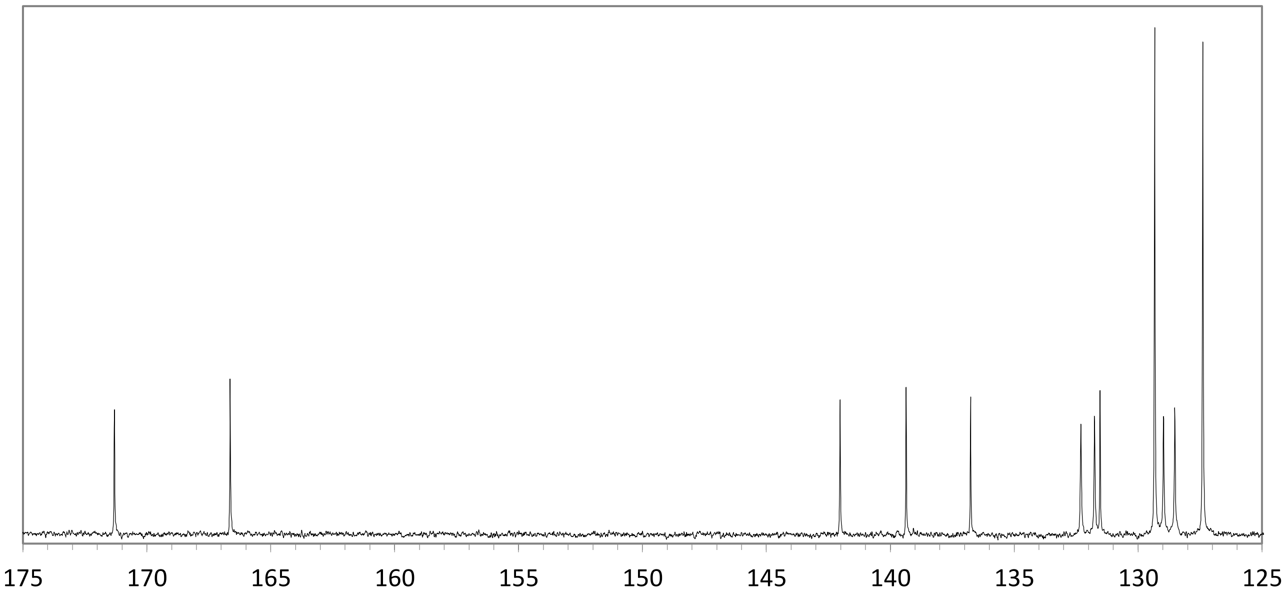

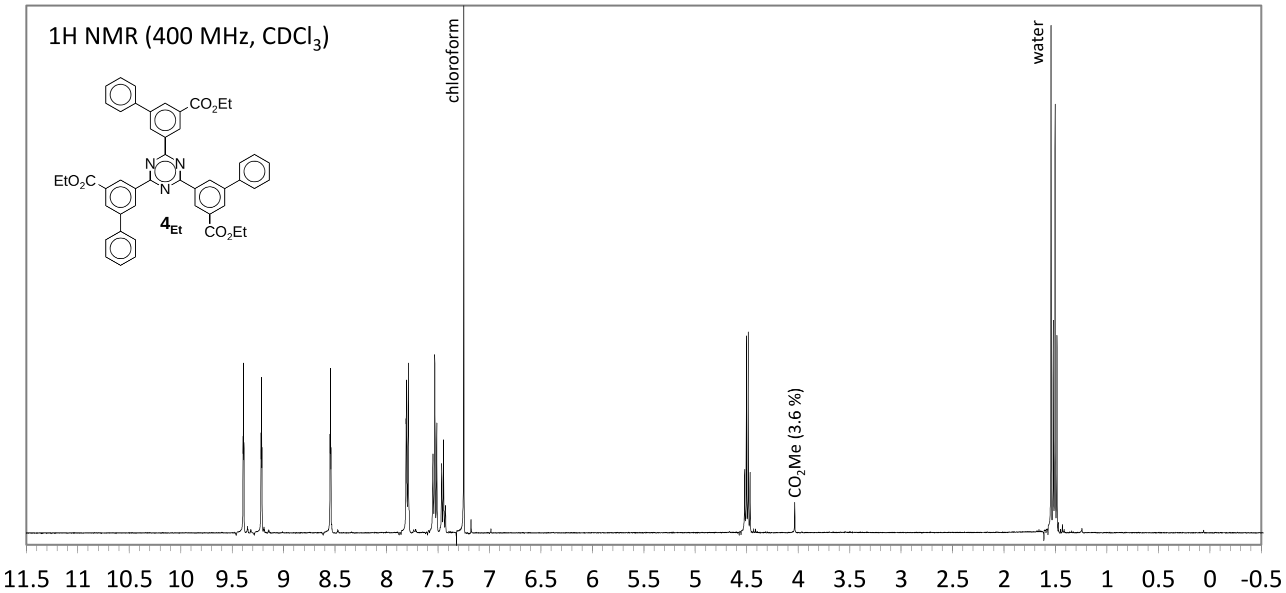
1H NMR (400 MHz, CDCl3)
chloroform
water
CO2Me (3.6 %)
CO2Et
EtO2C
CO2Et
4Et
11.5 11 10.5 10 9.5 9 8.5 8 7.5 7 6.5 6 5.5 5 4.5 4 3.5 3 2.5 2 1.5 1 0.5 0 -0.5

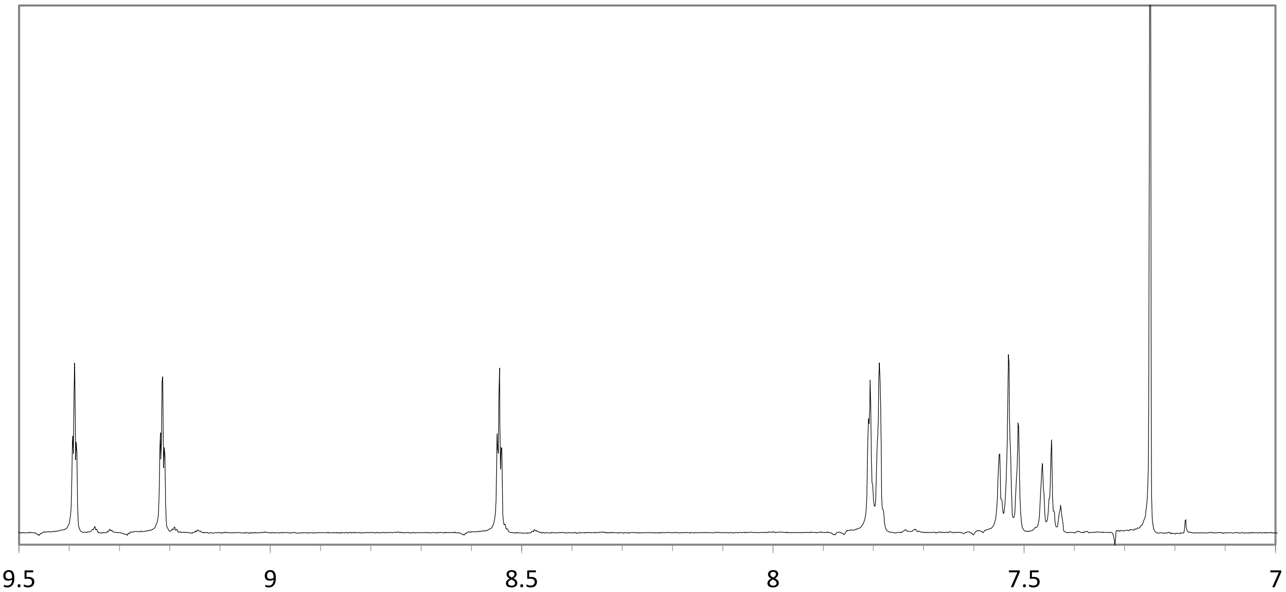
9.5 9 8.5 8 7.5 7

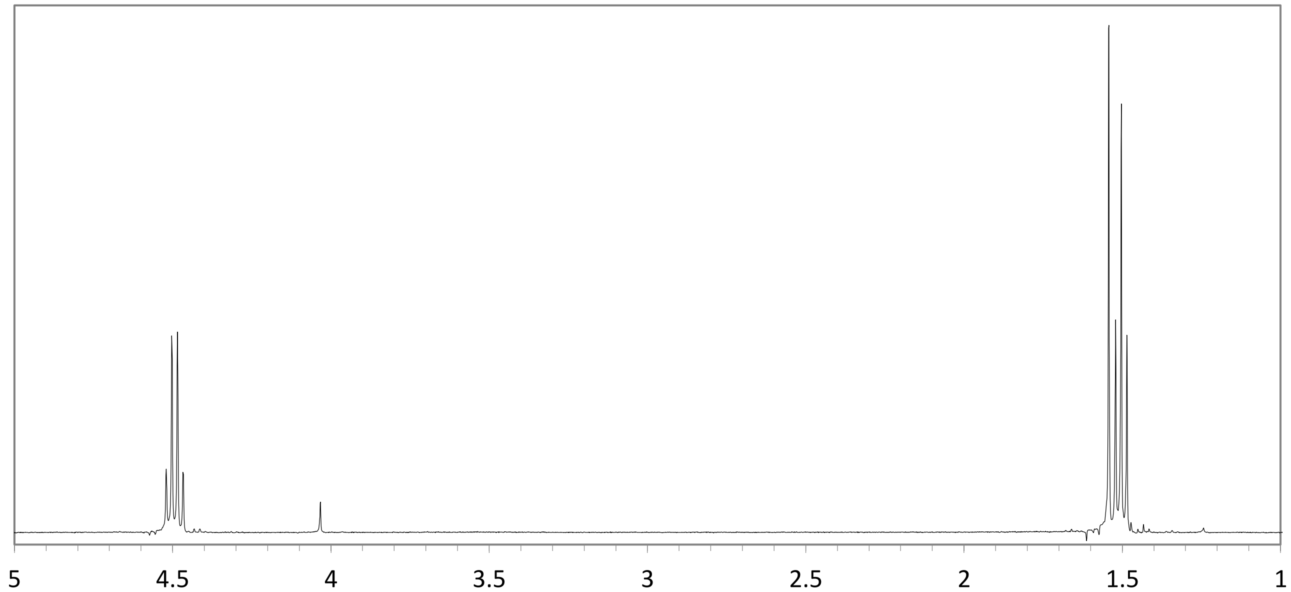
5 4.5 4 3.5 3 2.5 2 1.5 1

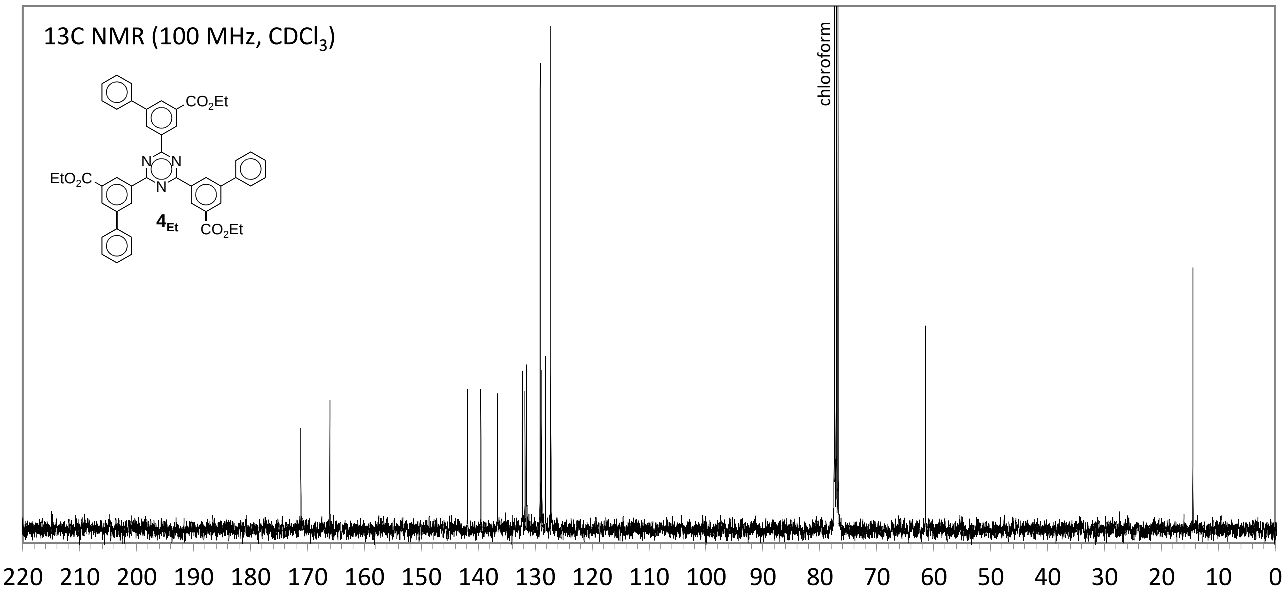
13C NMR (100 MHz, CDCl3)
CO2Et
EtO2C
N
N
N
4Et
CO2Et
chloroform
220 210 200 190 180 170 160 150 140 130 120 110 100 90 80 70 60 50 40 30 20 10 0

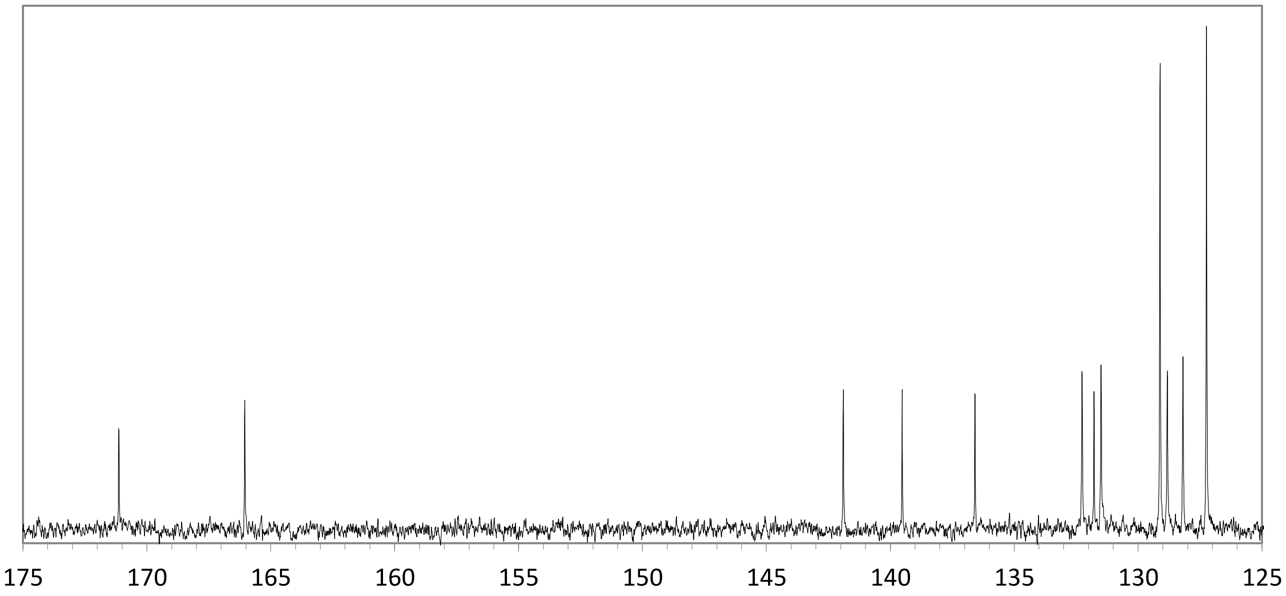
175 170 165 160 155 150 145 140 135 130 125

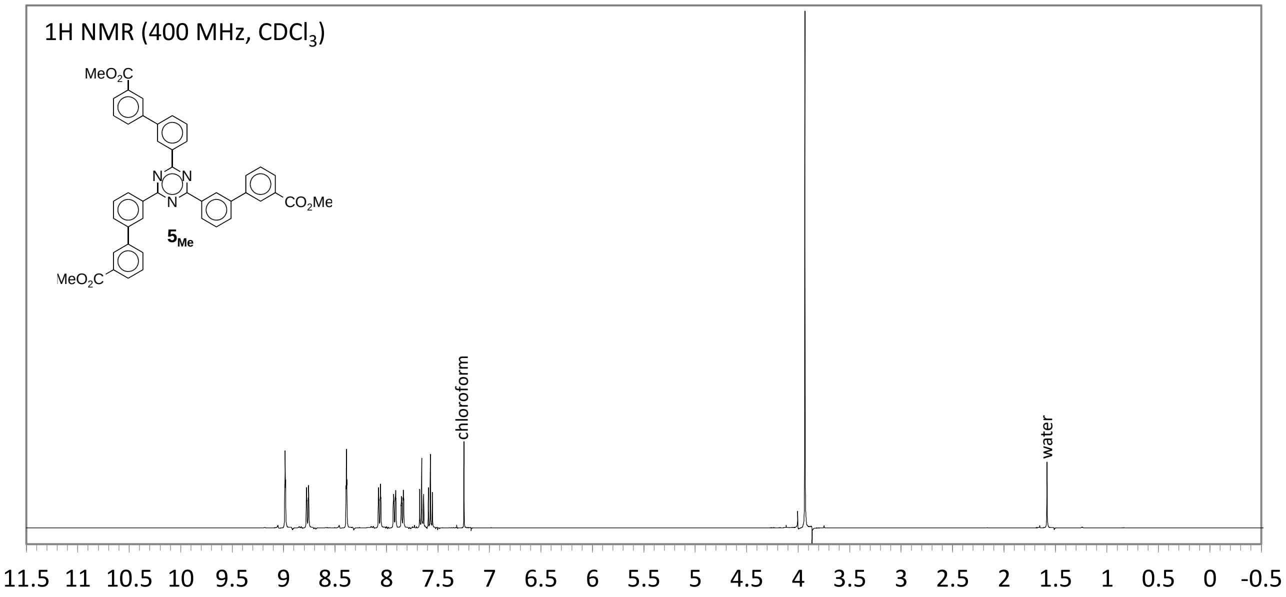
1H NMR (400 MHz, CDCl3)
MeO2C
N
N
N
CO2Me
5Me
MeO2C
chloroform
water
11.5 11 10.5 10 9.5 9 8.5 8 7.5 7 6.5 6 5.5 5 4.5 4 3.5 3 2.5 2 1.5 1 0.5 0 -0.5

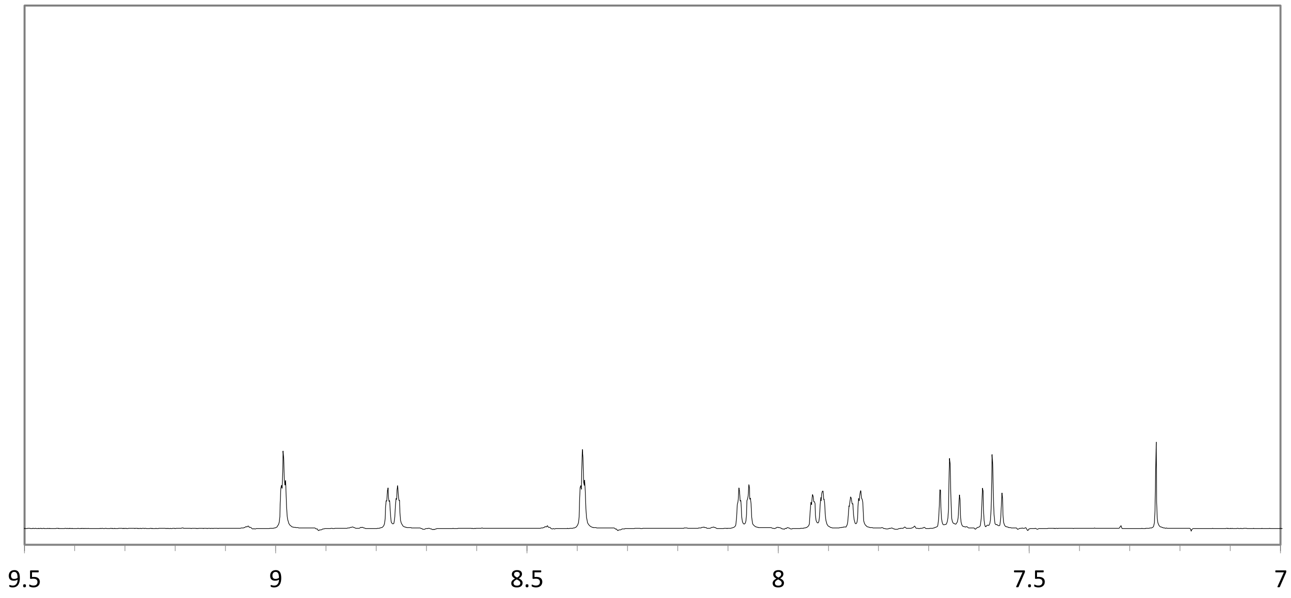
9.5
9
8.5
8
7.5
7

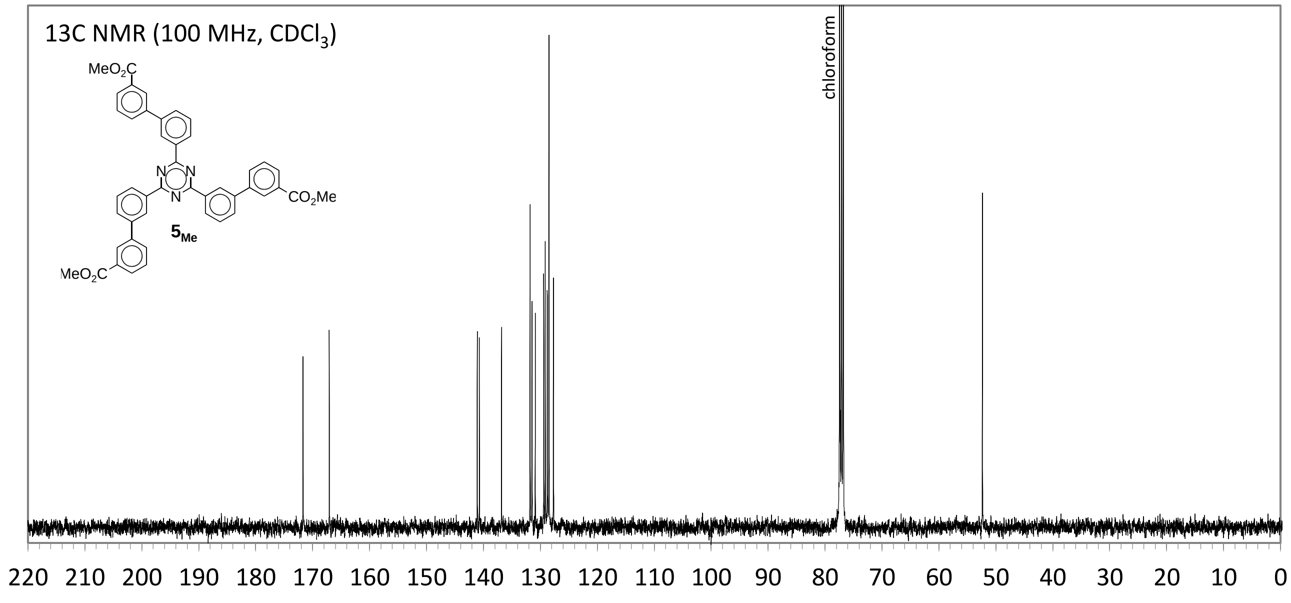
13C NMR (100 MHz, $CDCl_3$)
chloroform
MeO_2C
CO_2Me
MeO_2C
N
N
N
5_{Me}
220 210 200 190 180 170 160 150 140 130 120 110 100 90 80 70 60 50 40 30 20 10 0

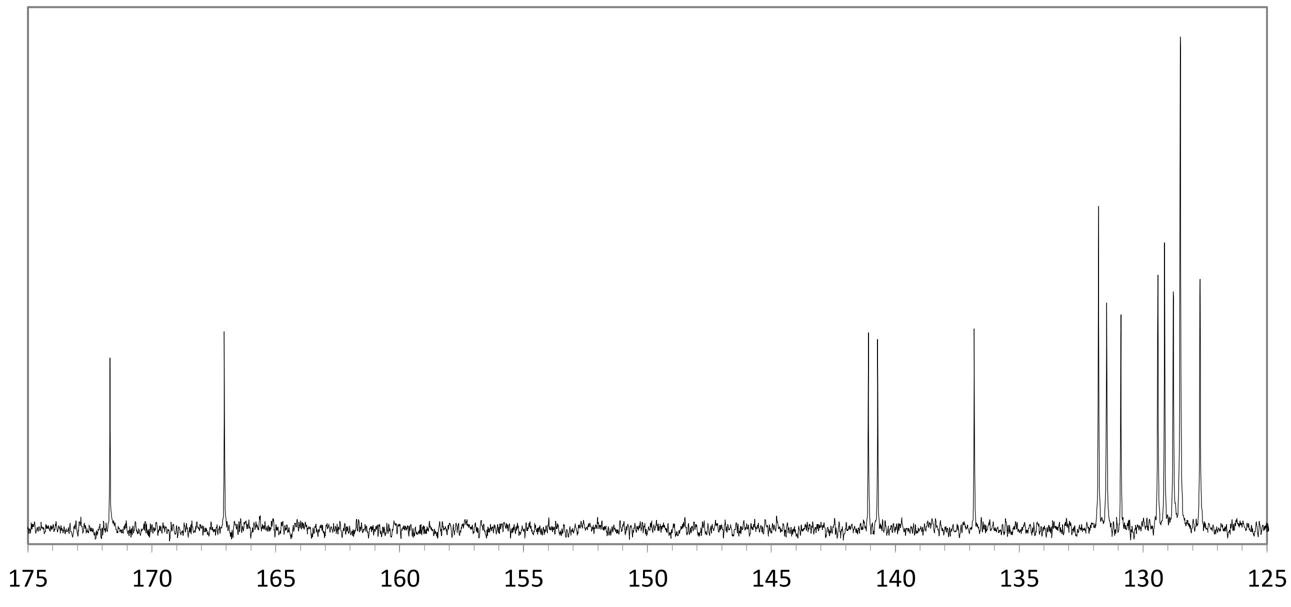
175 170 165 160 155 150 145 140 135 130 125

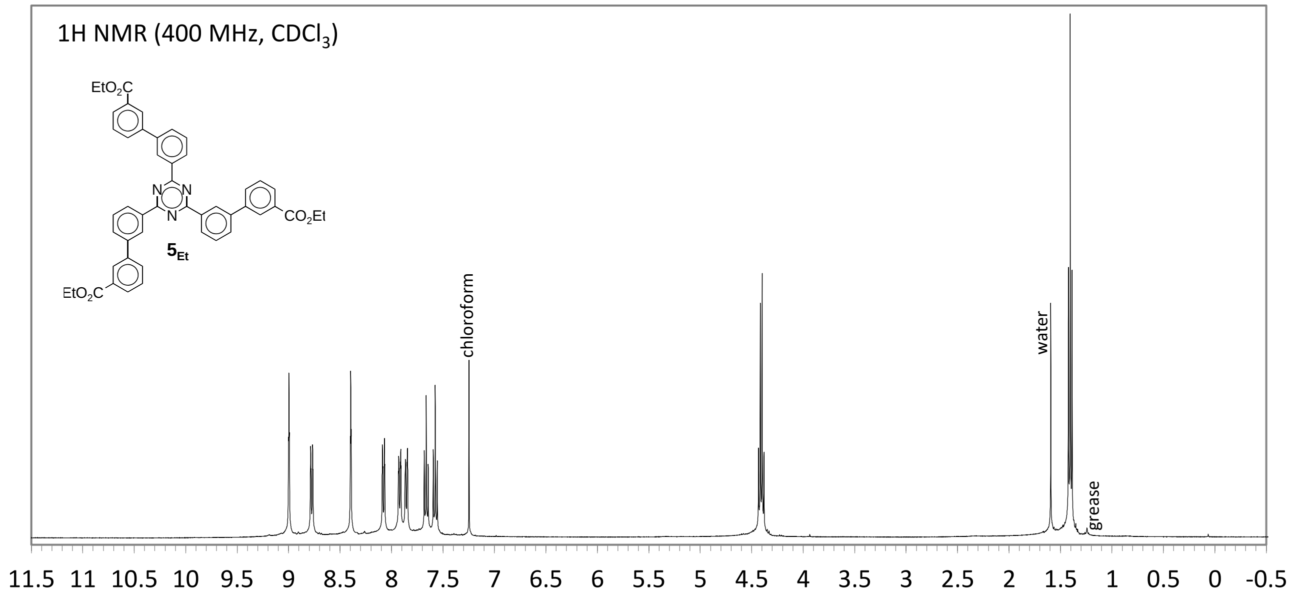

1H NMR (400 MHz, CDCl$_3$)
EtO$_2$C
CO$_2$Et
EtO$_2$C
5_{Et}
chloroform
water
grease
11.5 11 10.5 10 9.5 9 8.5 8 7.5 7 6.5 6 5.5 5 4.5 4 3.5 3 2.5 2 1.5 1 0.5 0 -0.5

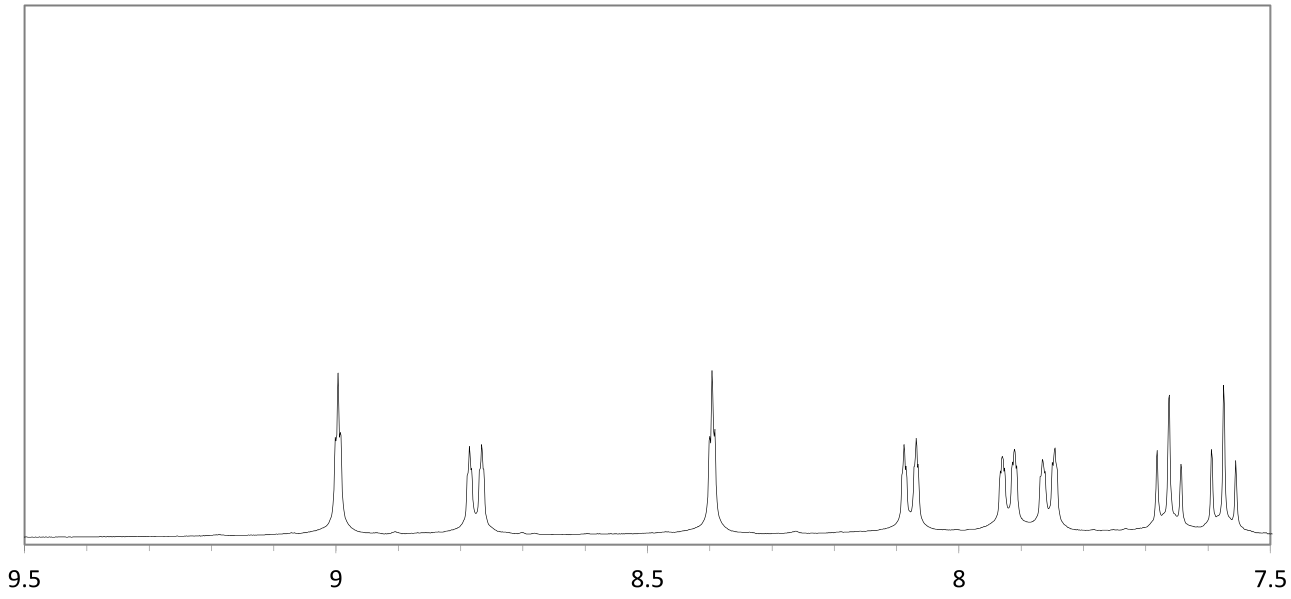

9.5 9 8.5 8 7.5

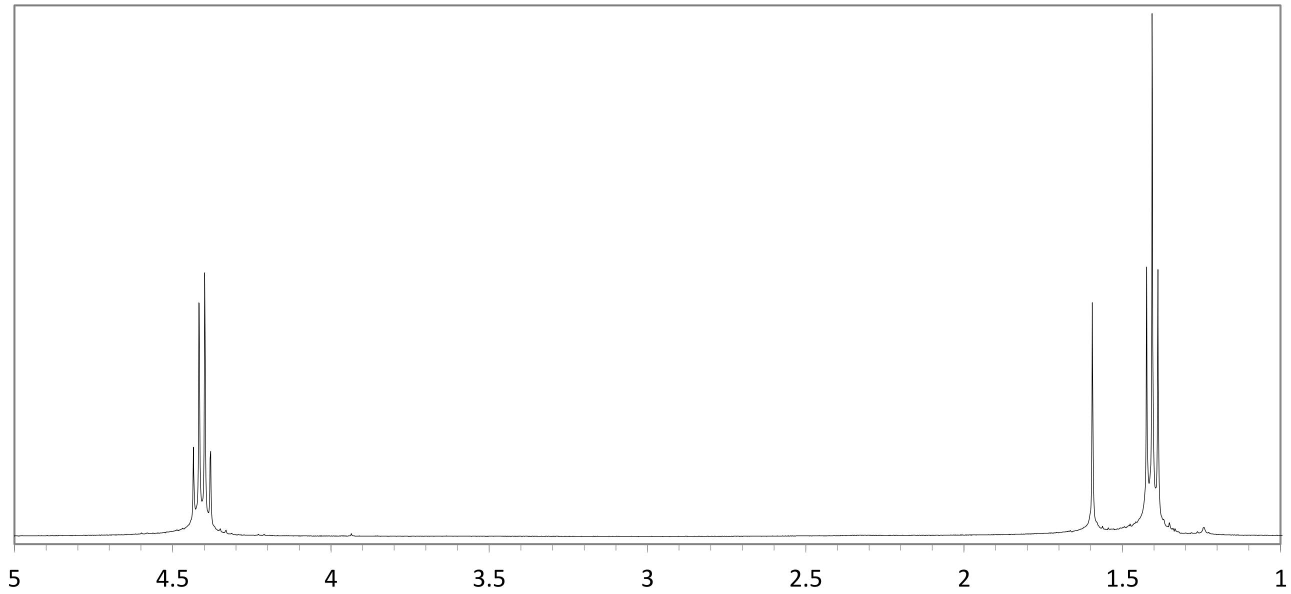

5 4.5 4 3.5 3 2.5 2 1.5 1

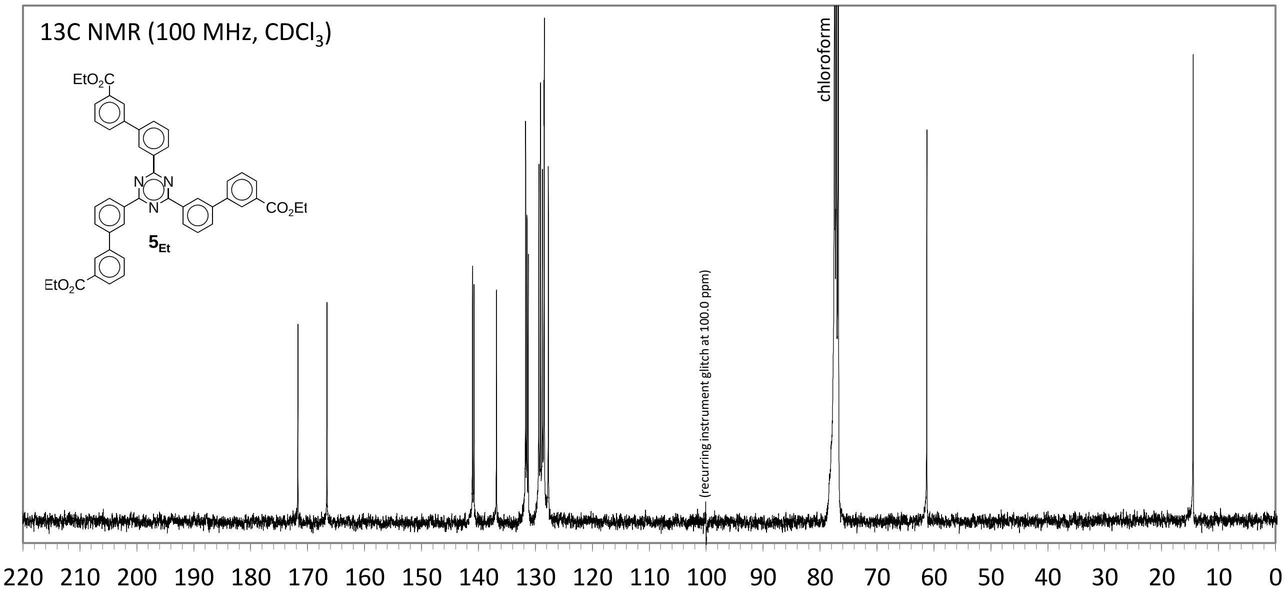
13C NMR (100 MHz, $CDCl_3$)
EtO_2C
CO_2Et
5_{Et}
chloroform
(recurring instrument glitch at 100.0 ppm)
220 210 200 190 180 170 160 150 140 130 120 110 100 90 80 70 60 50 40 30 20 10 0

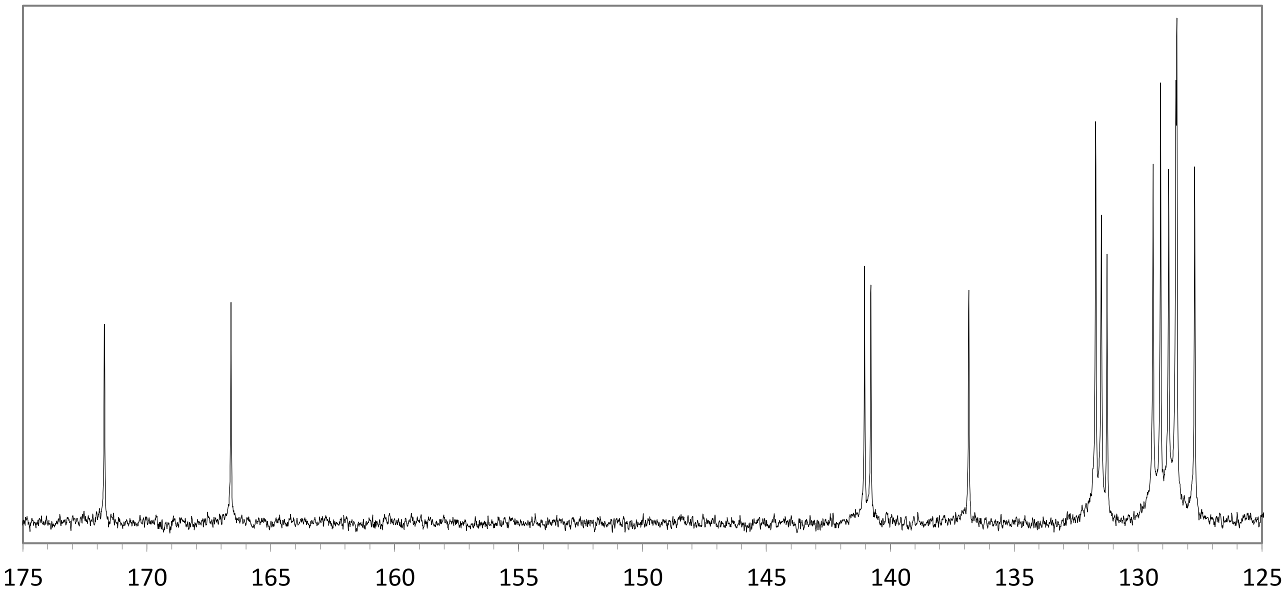
175 170 165 160 155 150 145 140 135 130 125

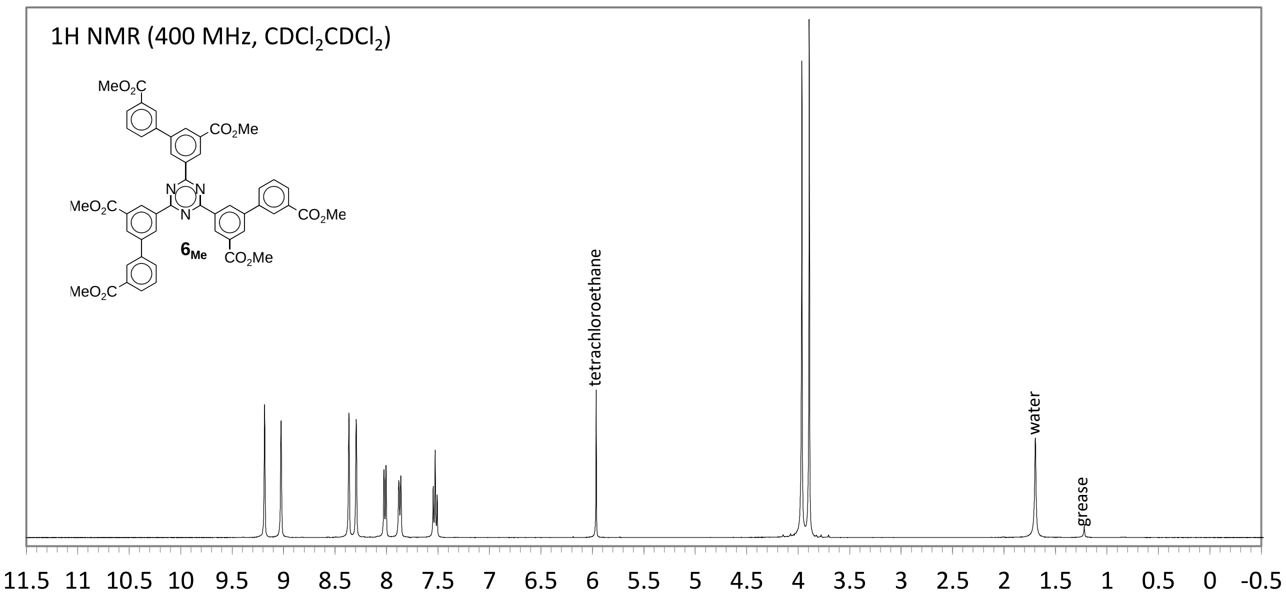
1H NMR (400 MHz, $CDCl_2CDCl_2$)
MeO_2C
CO_2Me
6_{Me}
tetrachloroethane
water
grease
11.5 11 10.5 10 9.5 9 8.5 8 7.5 7 6.5 6 5.5 5 4.5 4 3.5 3 2.5 2 1.5 1 0.5 0 -0.5

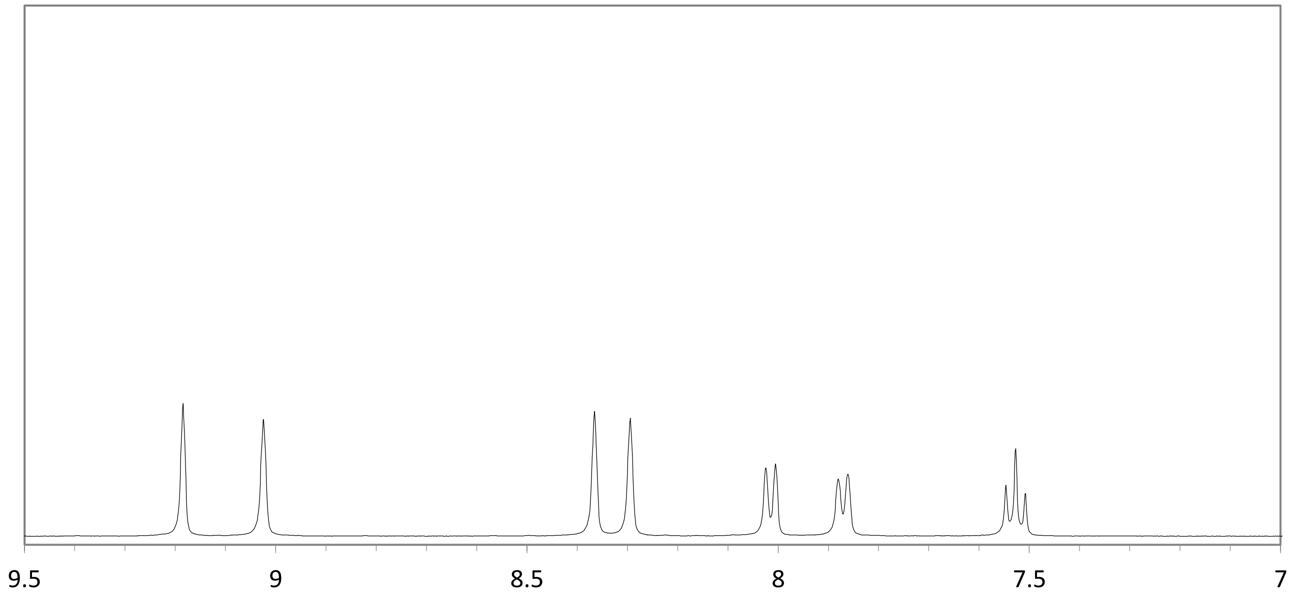
9.5
9
8.5
8
7.5
7

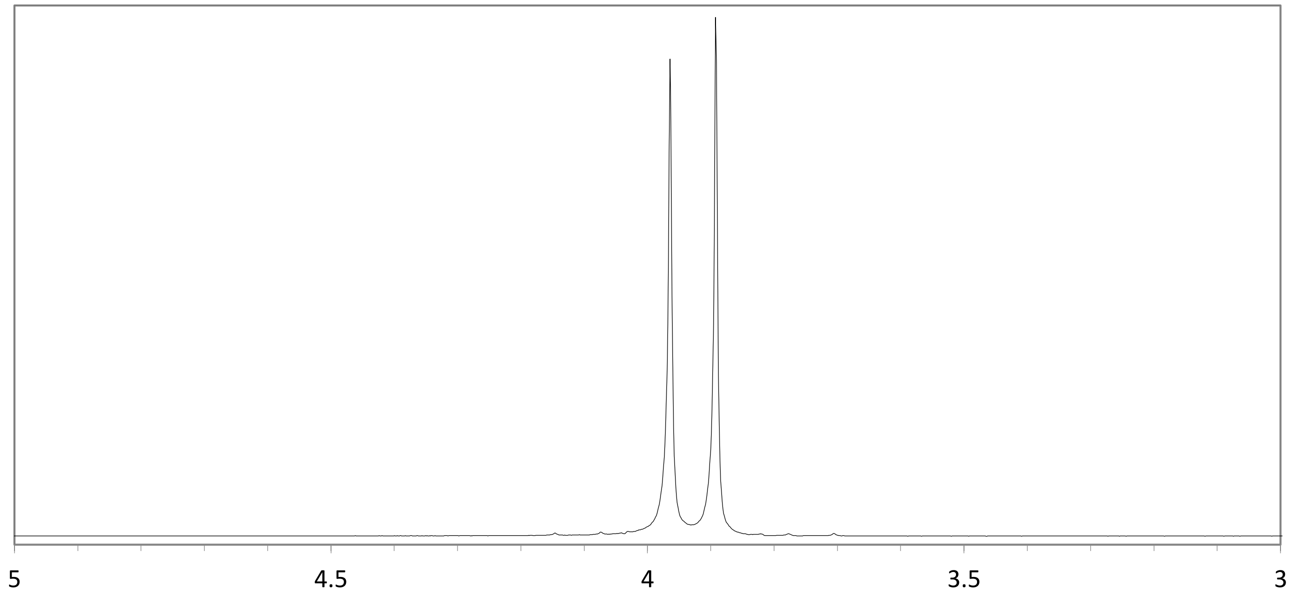
5
4.5
4
3.5
3

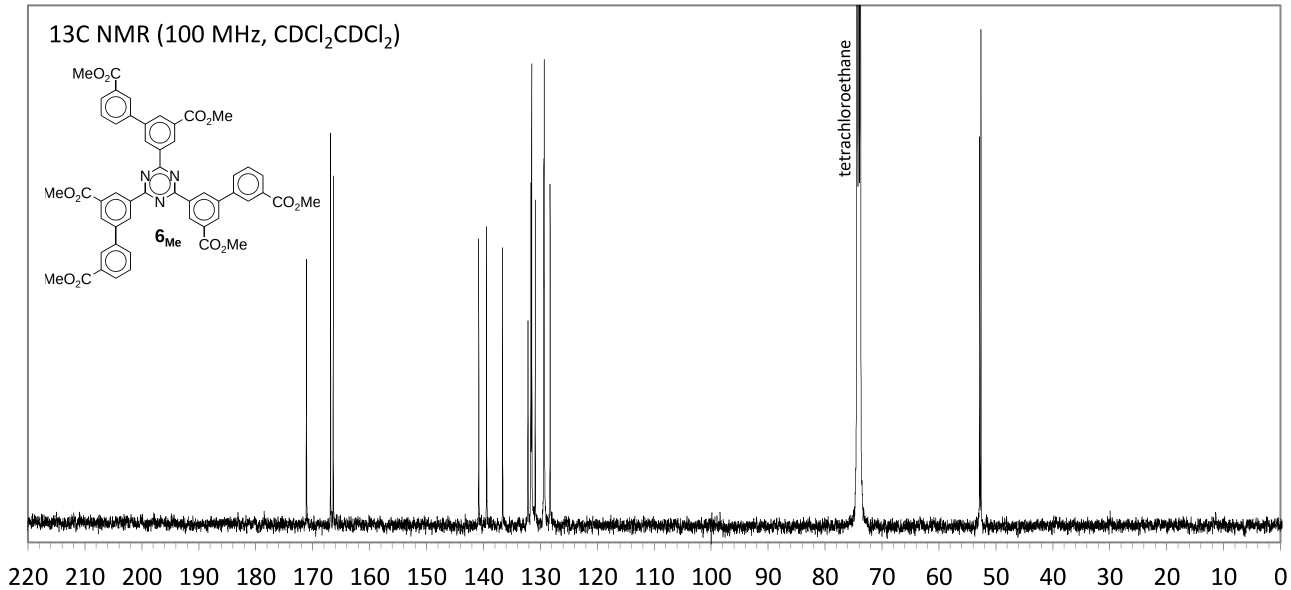
13C NMR (100 MHz, $CDCl_2CDCl_2$)
MeO_2C
CO_2Me
MeO_2C
CO_2Me
$\mathbf{6}_{Me}$
CO_2Me
MeO_2C
tetrachloroethane
220 210 200 190 180 170 160 150 140 130 120 110 100 90 80 70 60 50 40 30 20 10 0

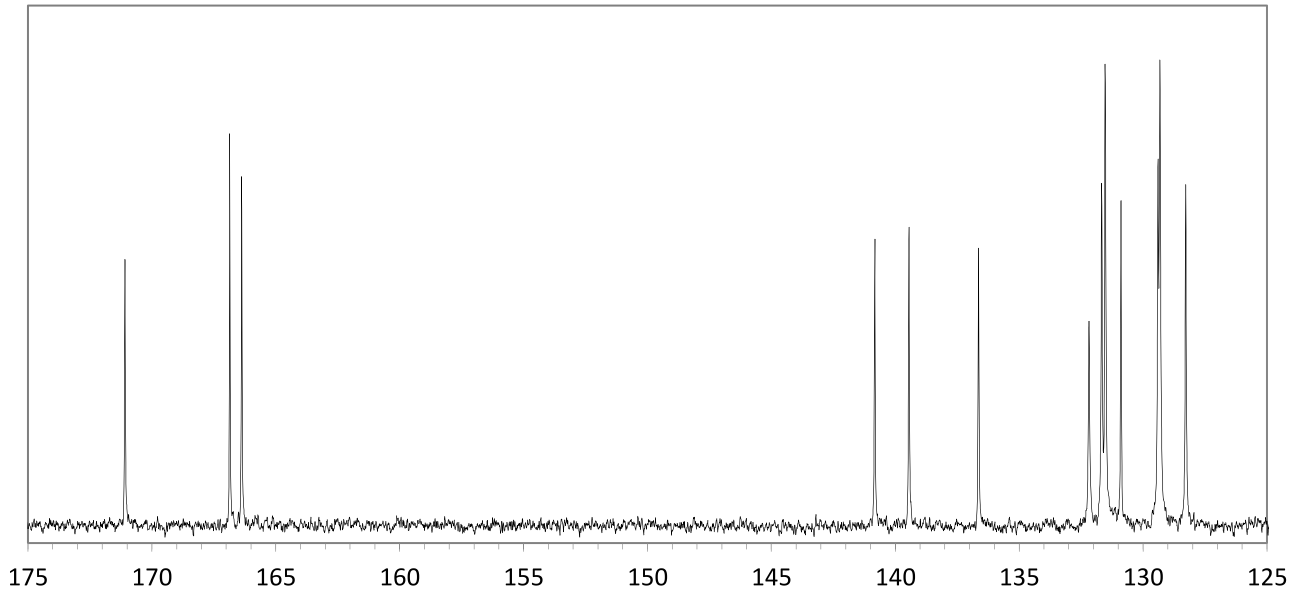
175 170 165 160 155 150 145 140 135 130 125

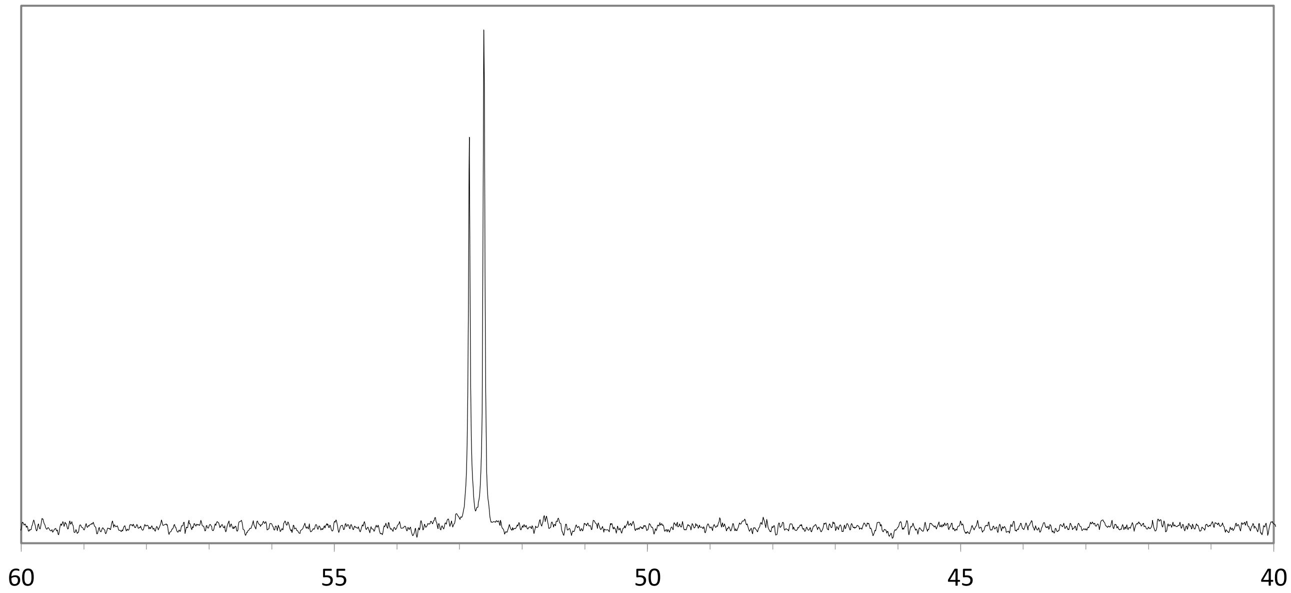
60 55 50 45 40

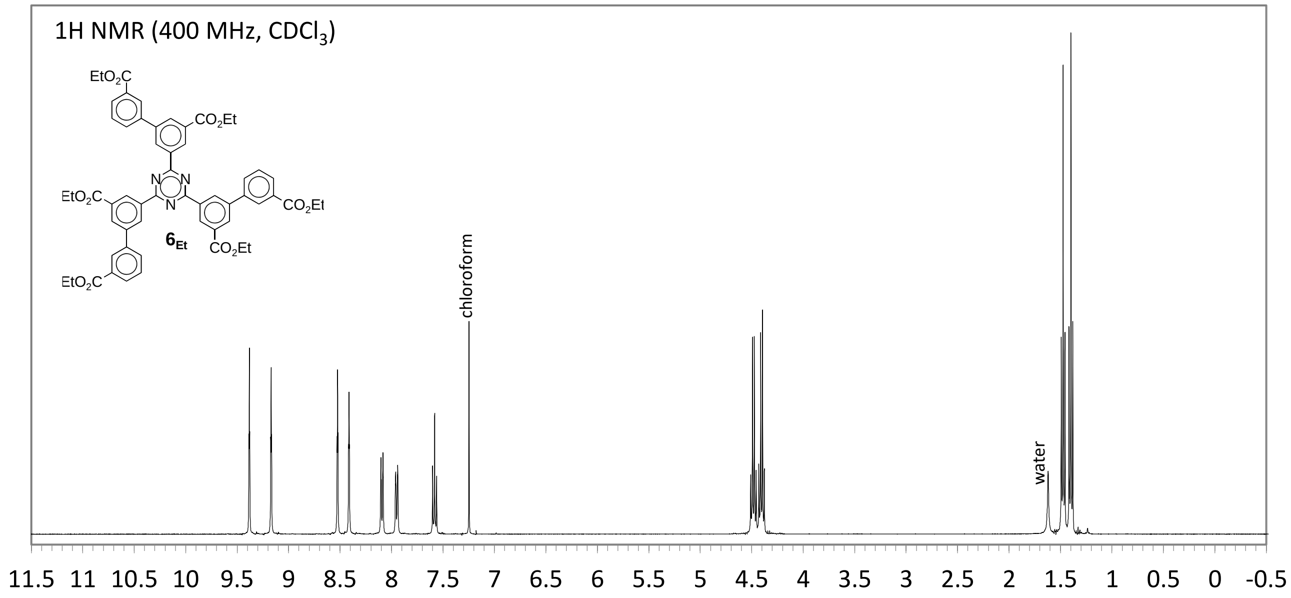
1H NMR (400 MHz, CDCl$_3$)
EtO$_2$C
CO$_2$Et
N
EtO$_2$C
CO$_2$Et
6$_{Et}$
CO$_2$Et
EtO$_2$C
chloroform
water
11.5 11 10.5 10 9.5 9 8.5 8 7.5 7 6.5 6 5.5 5 4.5 4 3.5 3 2.5 2 1.5 1 0.5 0 -0.5

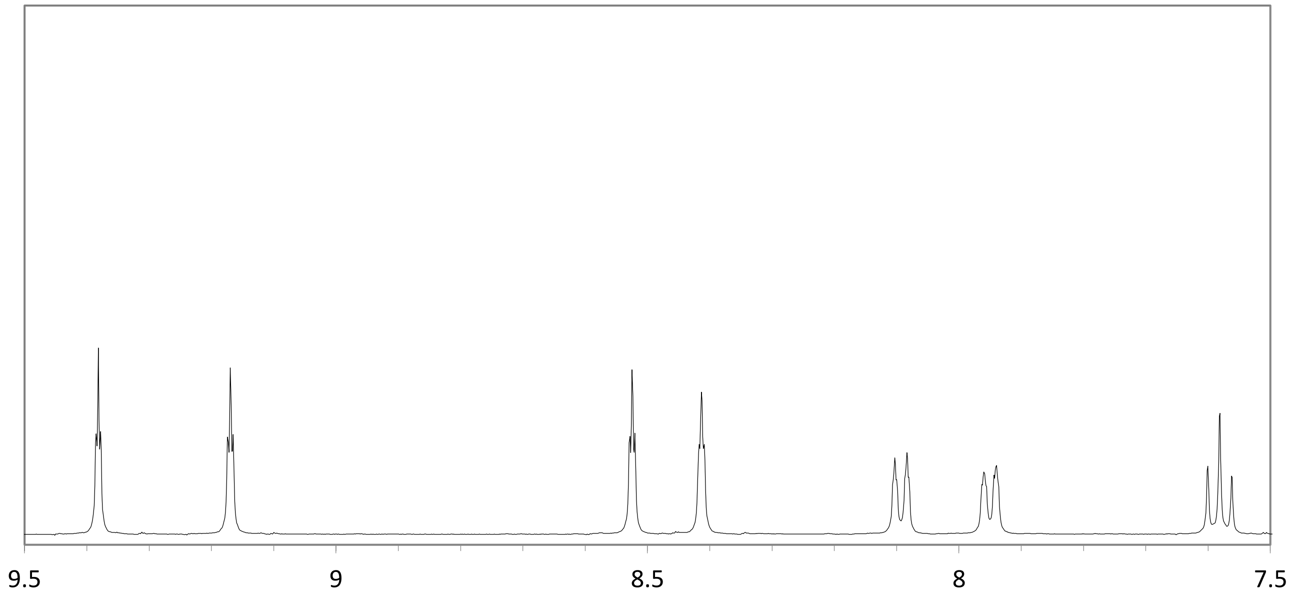
9.5
9
8.5
8
7.5

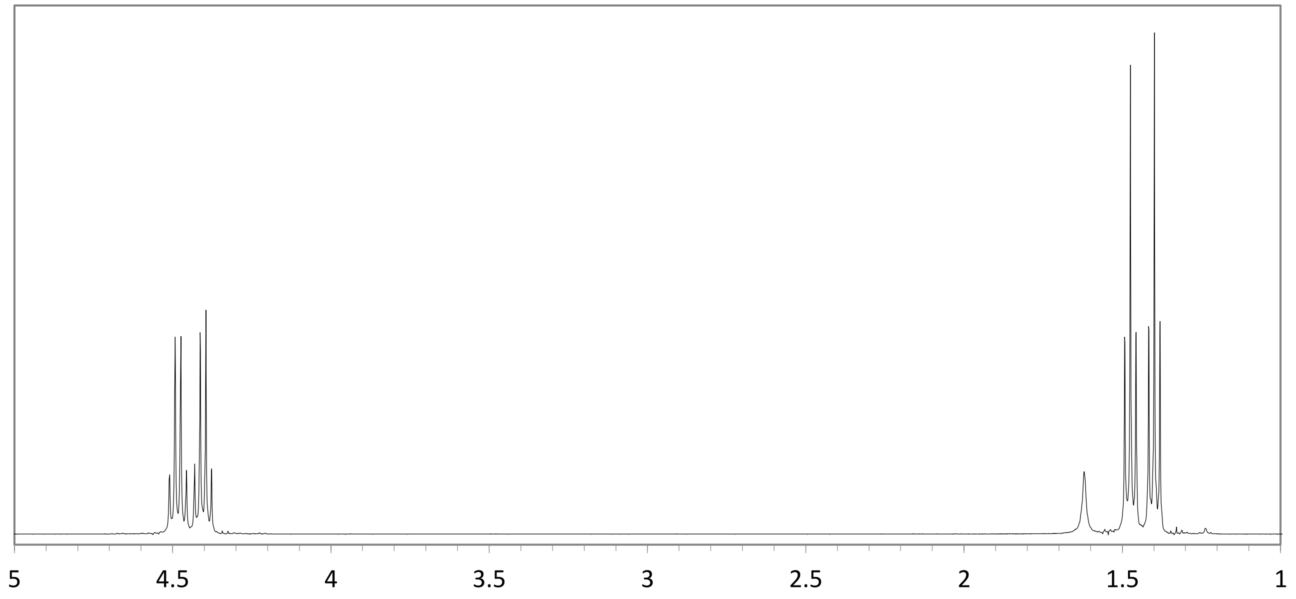
5
4.5
4
3.5
3
2.5
2
1.5
1

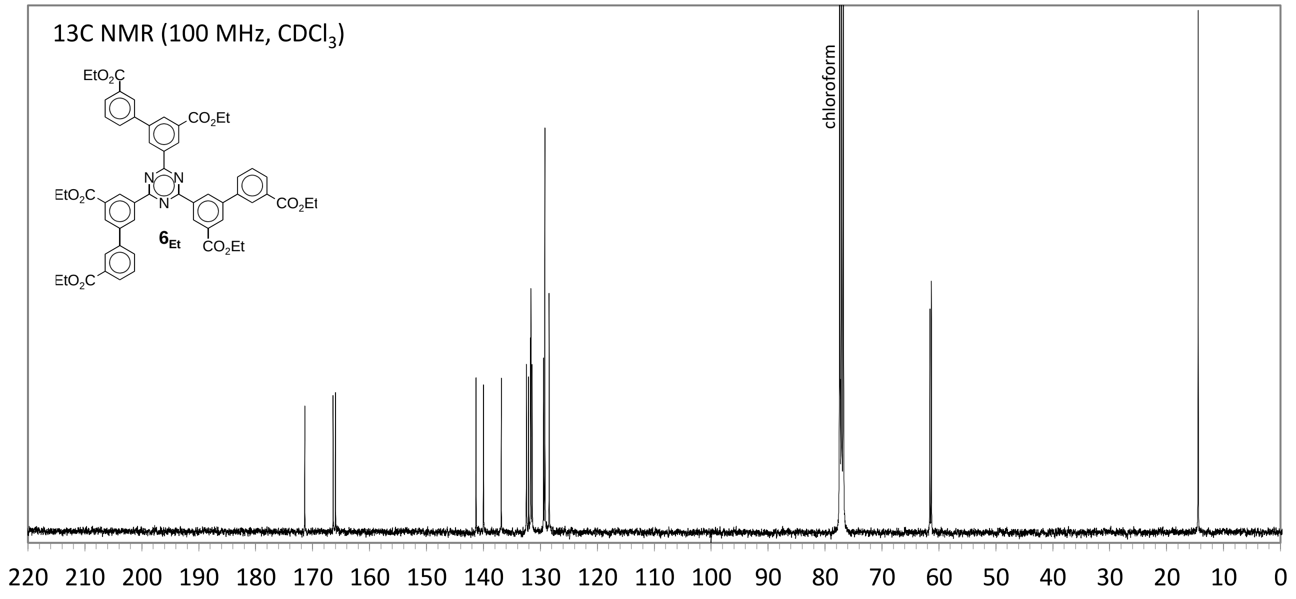
13C NMR (100 MHz, CDCl3)
EtO2C
CO2Et
EtO2C
CO2Et
6Et
CO2Et
EtO2C
chloroform
220 210 200 190 180 170 160 150 140 130 120 110 100 90 80 70 60 50 40 30 20 10 0

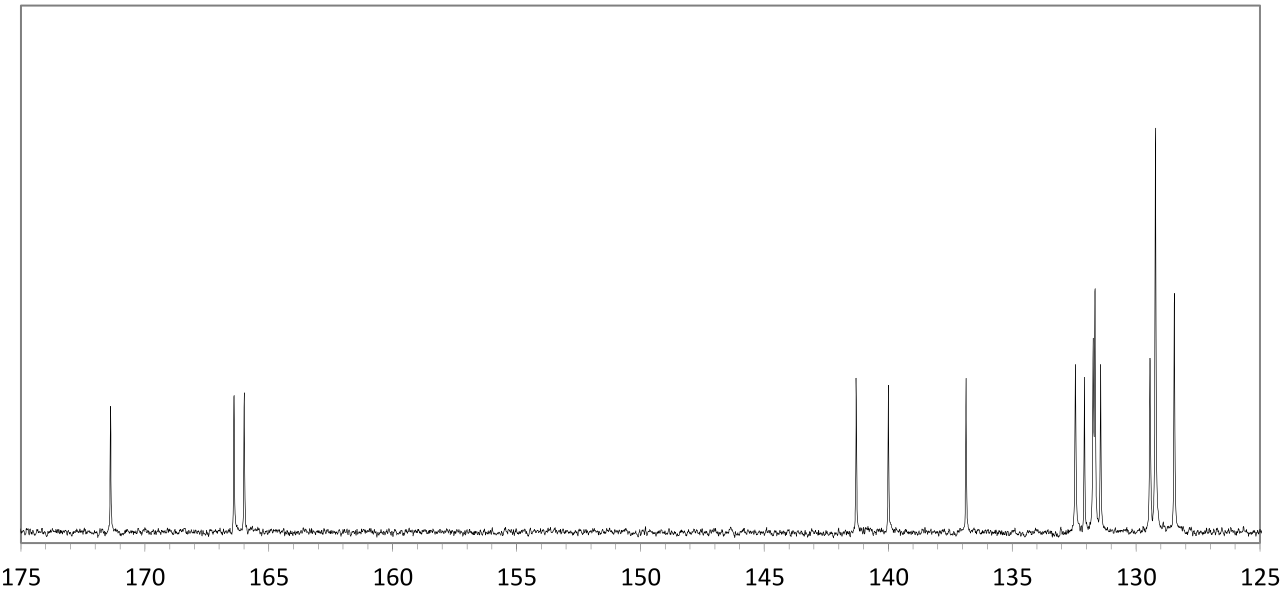
175
170
165
160
155
150
145
140
135
130
125

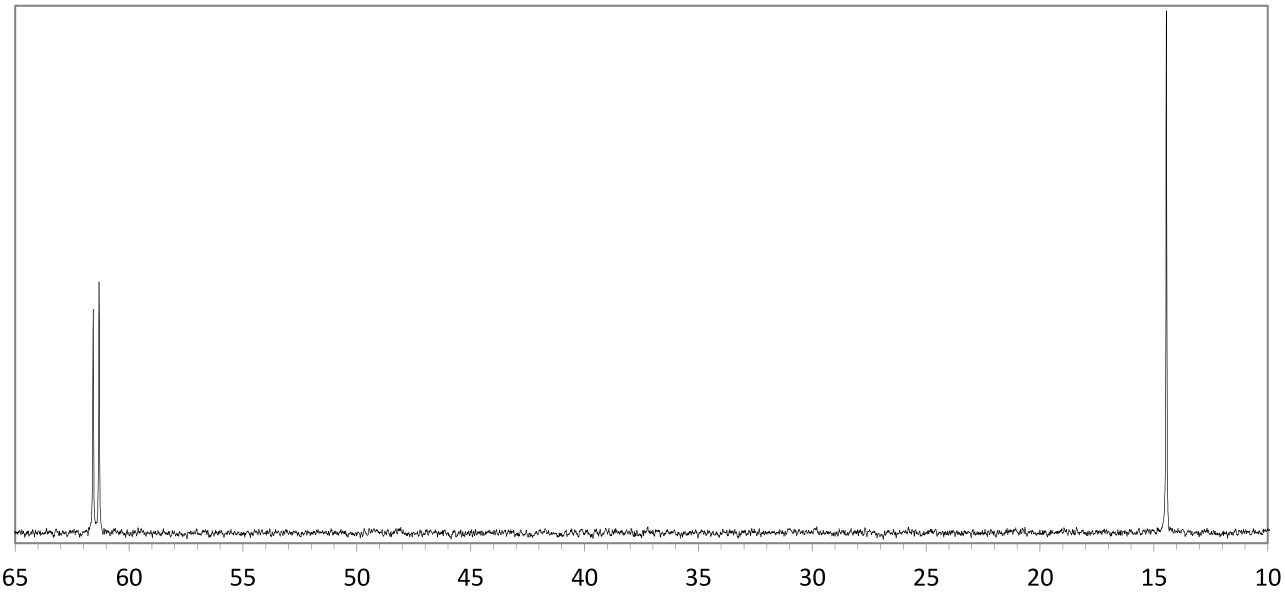
65
60
55
50
45
40
35
30
25
20
15
10

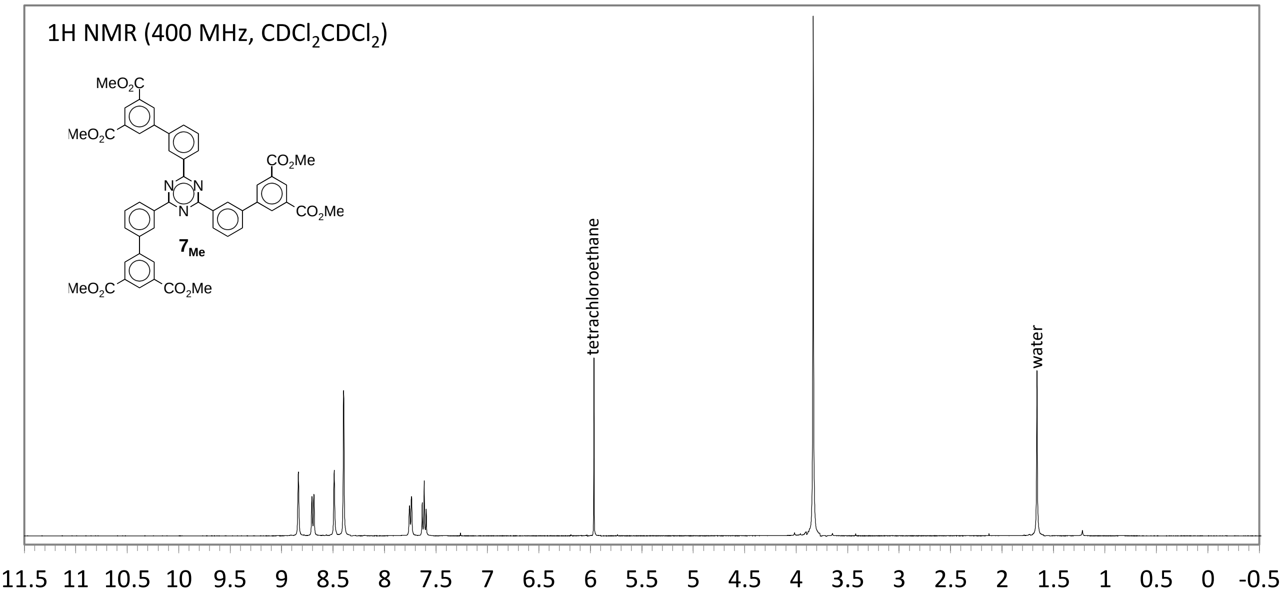
1H NMR (400 MHz, CDCl2CDCl2)
MeO2C
MeO2C
CO2Me
CO2Me
N
N
N
7Me
MeO2C
CO2Me
tetrachloroethane
water
11.5 11 10.5 10 9.5 9 8.5 8 7.5 7 6.5 6 5.5 5 4.5 4 3.5 3 2.5 2 1.5 1 0.5 0 -0.5

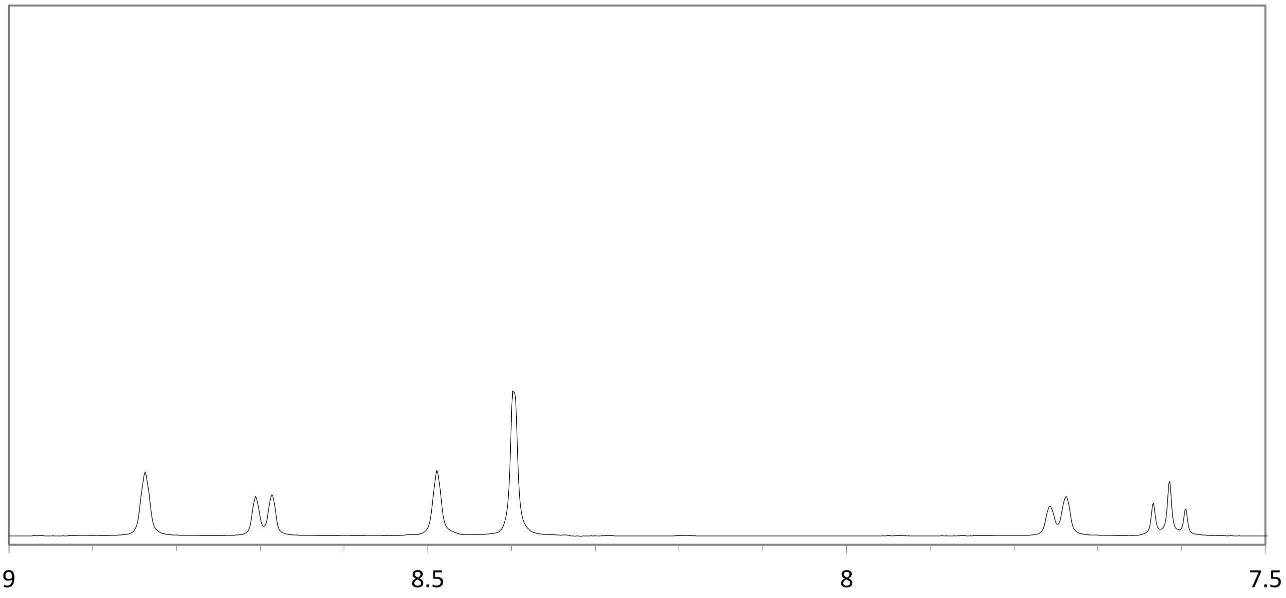
9
8.5
8
7.5

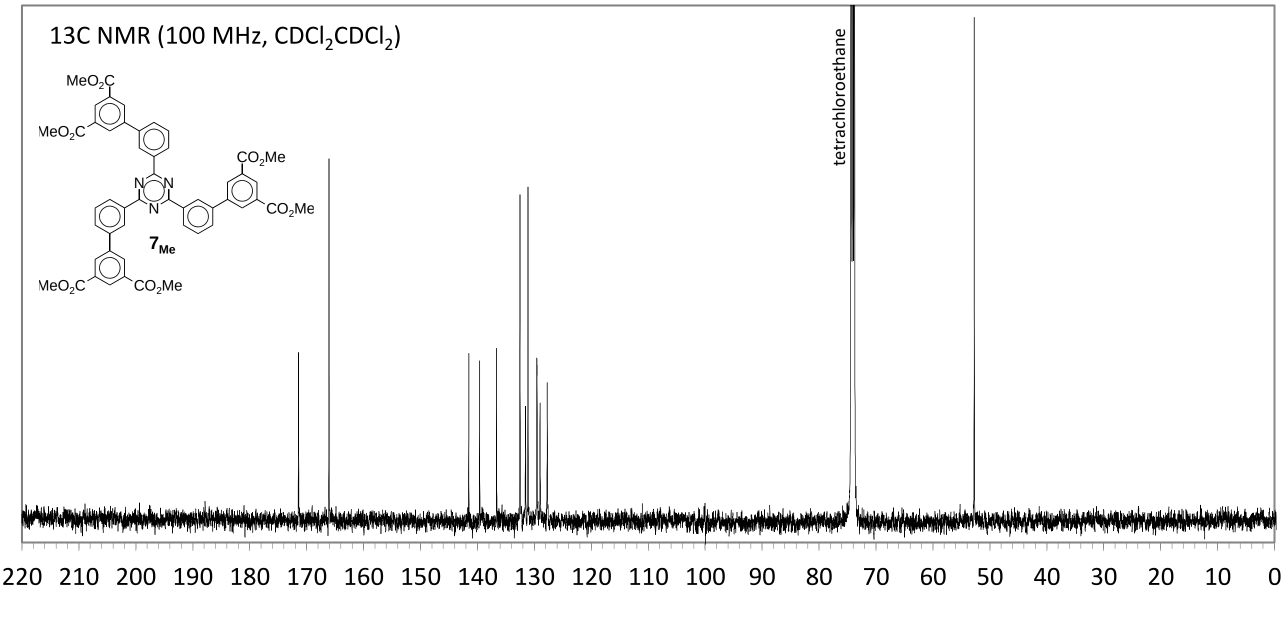
13C NMR (100 MHz, CDCl2CDCl2)
tetrachloroethane
7Me
220 210 200 190 180 170 160 150 140 130 120 110 100 90 80 70 60 50 40 30 20 10 0

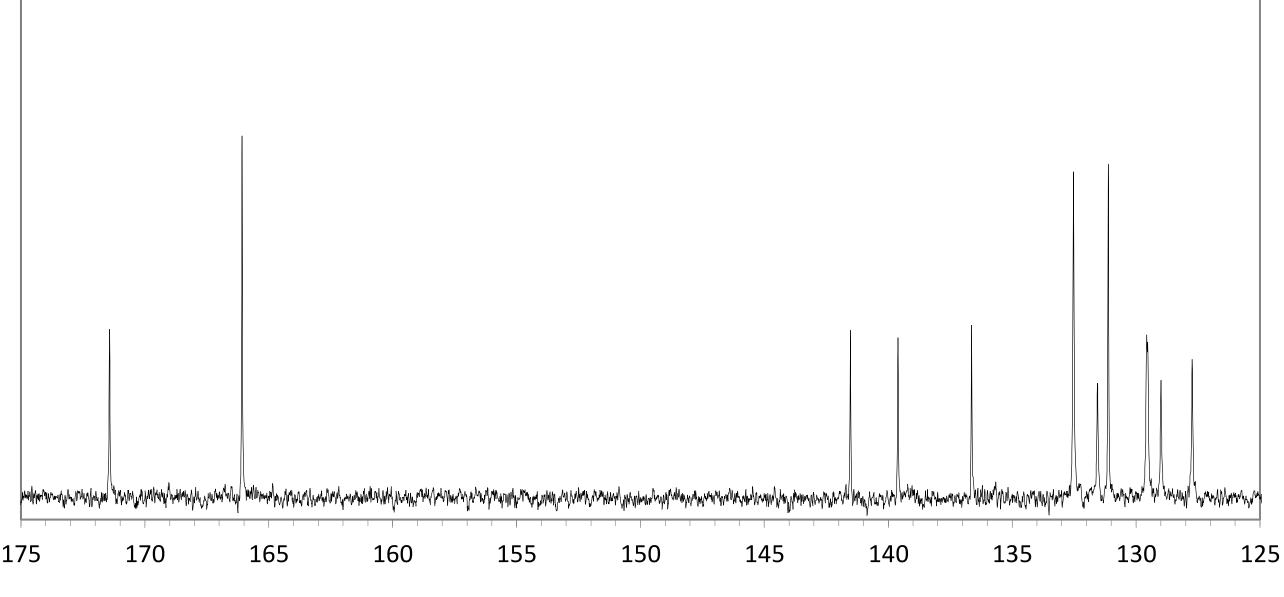
175 170 165 160 155 150 145 140 135 130 125

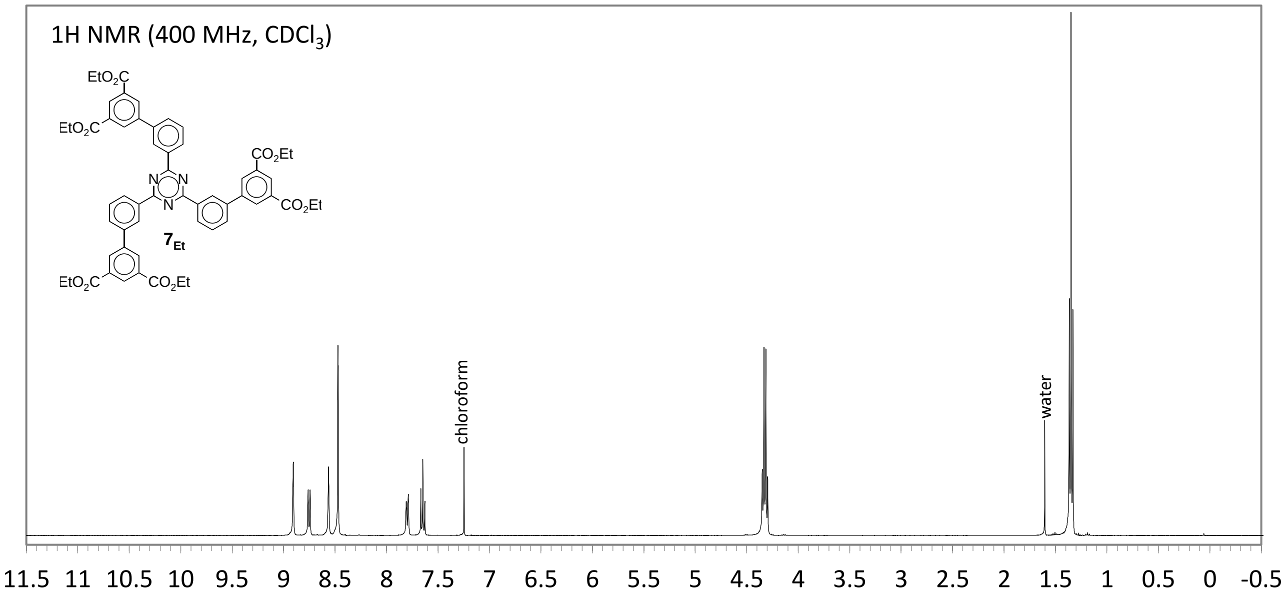
1H NMR (400 MHz, CDCl$_3$)
EtO$_2$C
EtO$_2$C
CO$_2$Et
CO$_2$Et
N
N
N
7$_{Et}$
EtO$_2$C
CO$_2$Et
chloroform
water
11.5 11 10.5 10 9.5 9 8.5 8 7.5 7 6.5 6 5.5 5 4.5 4 3.5 3 2.5 2 1.5 1 0.5 0 -0.5

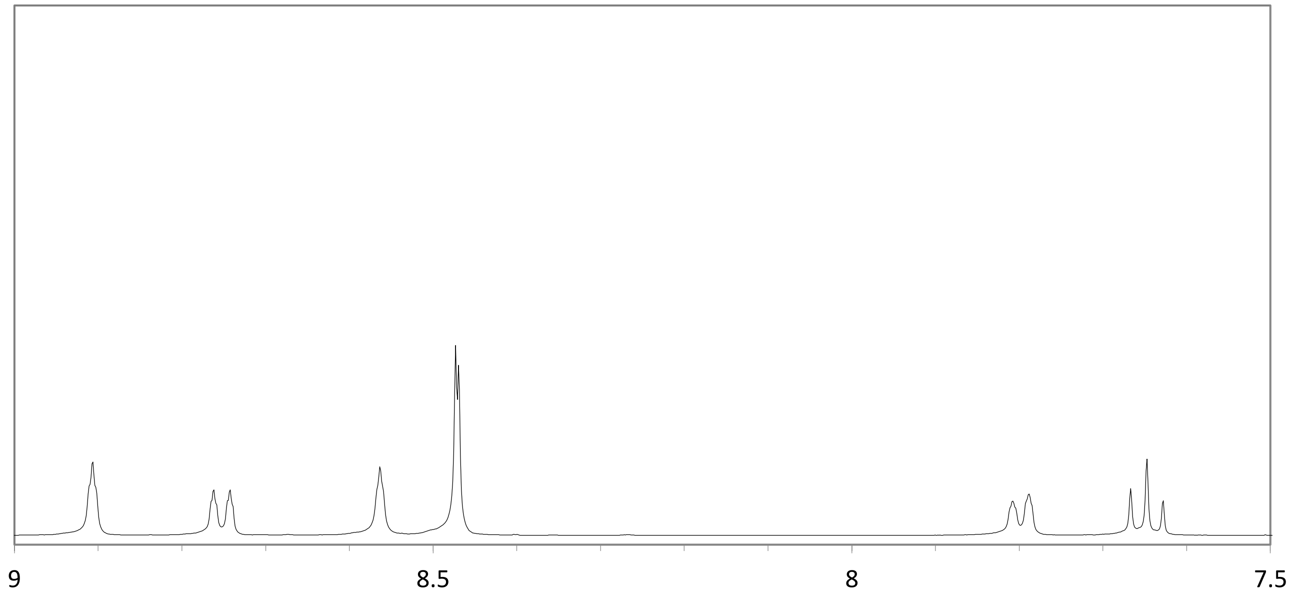
9
8.5
8
7.5

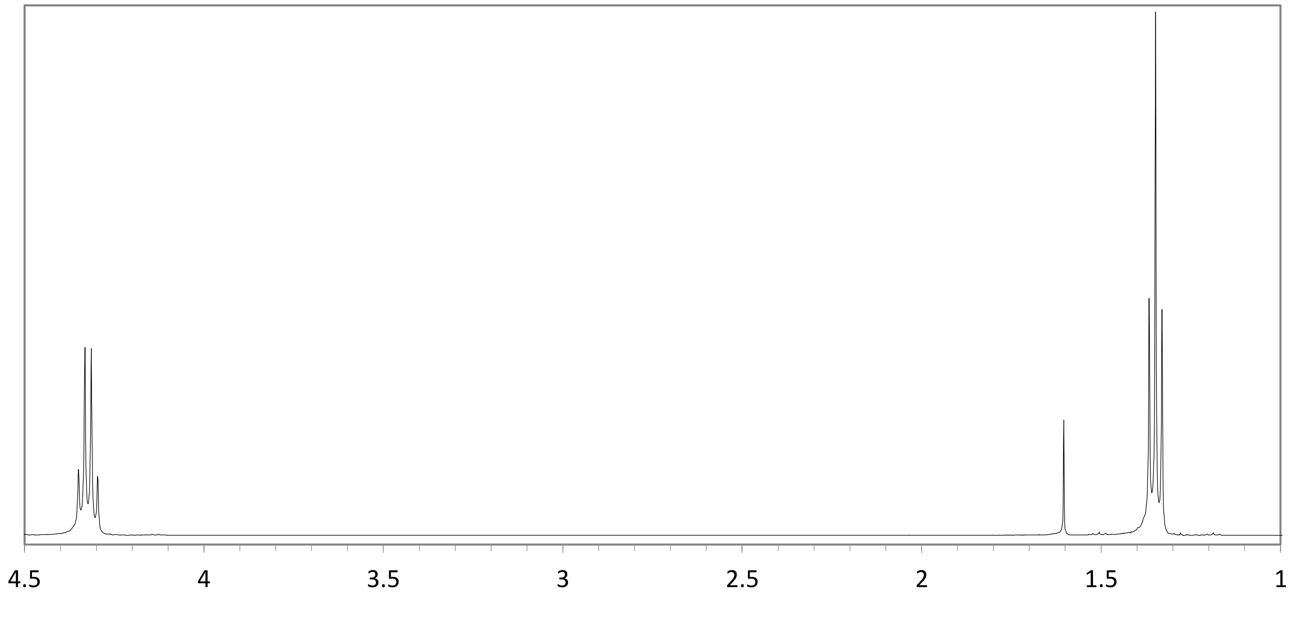
4.5
4
3.5
3
2.5
2
1.5
1

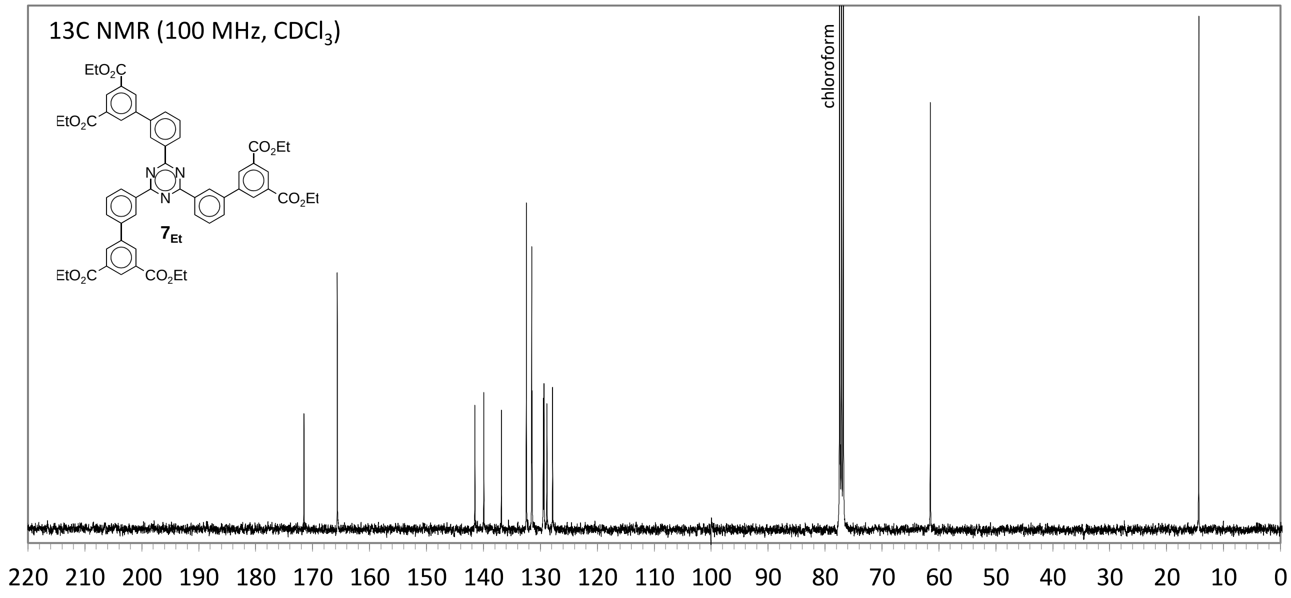
13C NMR (100 MHz, $CDCl_3$)
EtO_2C
CO_2Et
N
7_{Et}
chloroform
220 210 200 190 180 170 160 150 140 130 120 110 100 90 80 70 60 50 40 30 20 10 0

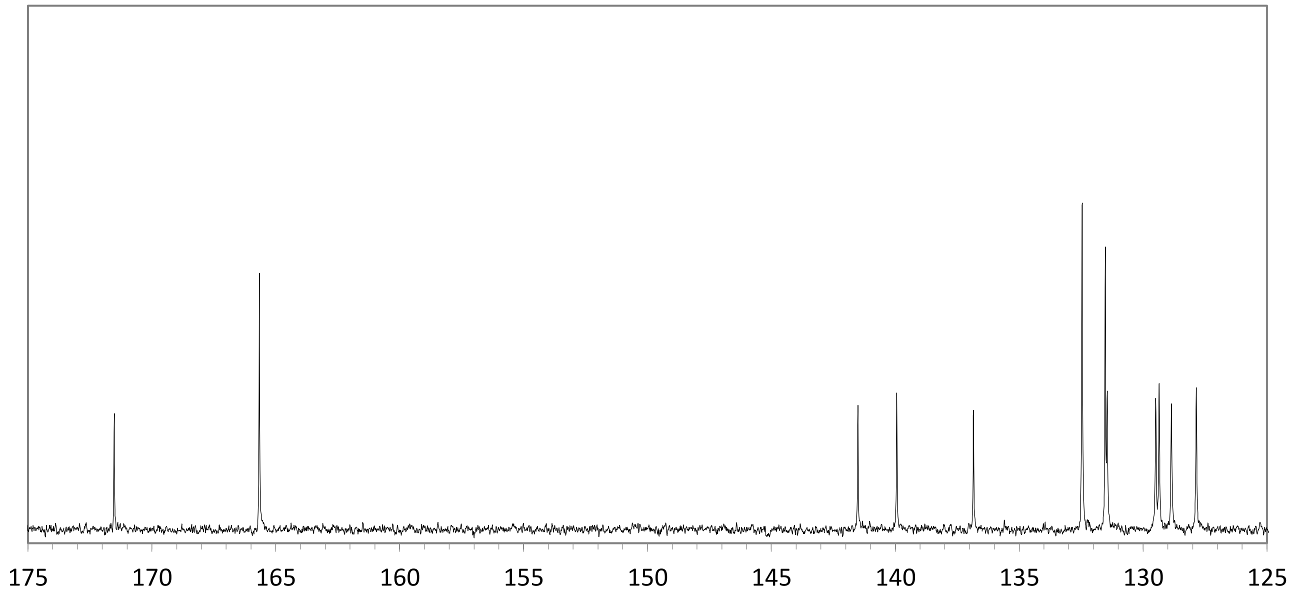
175 170 165 160 155 150 145 140 135 130 125

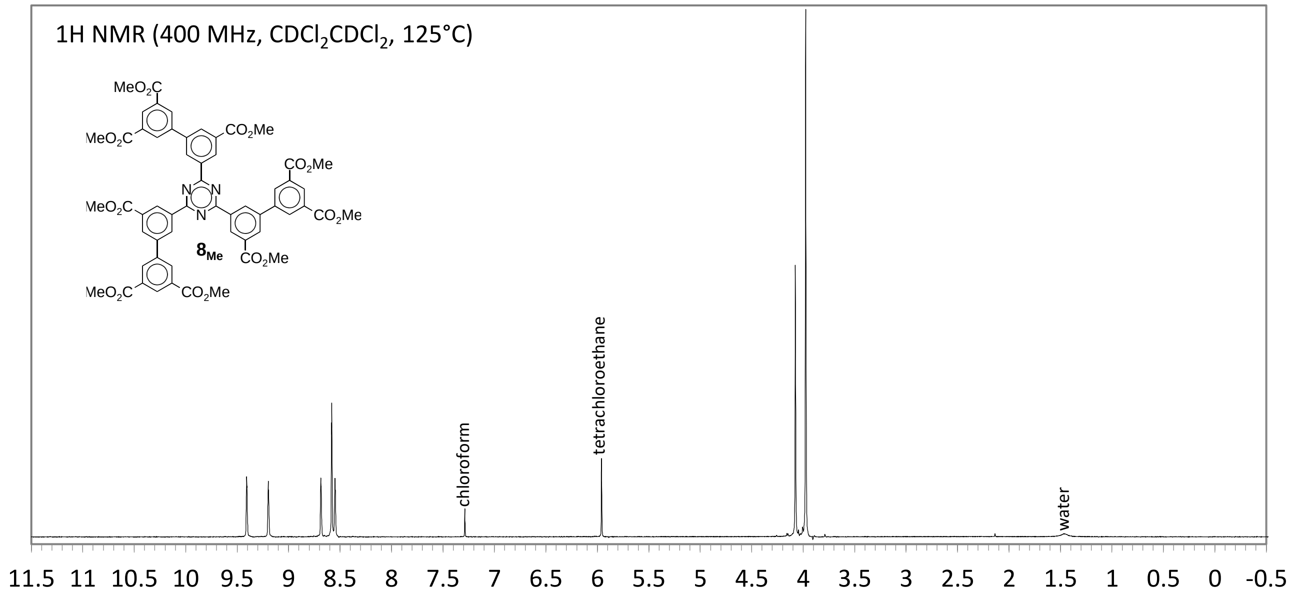
1H NMR (400 MHz, $CDCl_2CDCl_2$, 125°C)
MeO_2C
CO_2Me
N
8_{Me}
chloroform
tetrachloroethane
water
11.5 11 10.5 10 9.5 9 8.5 8 7.5 7 6.5 6 5.5 5 4.5 4 3.5 3 2.5 2 1.5 1 0.5 0 -0.5

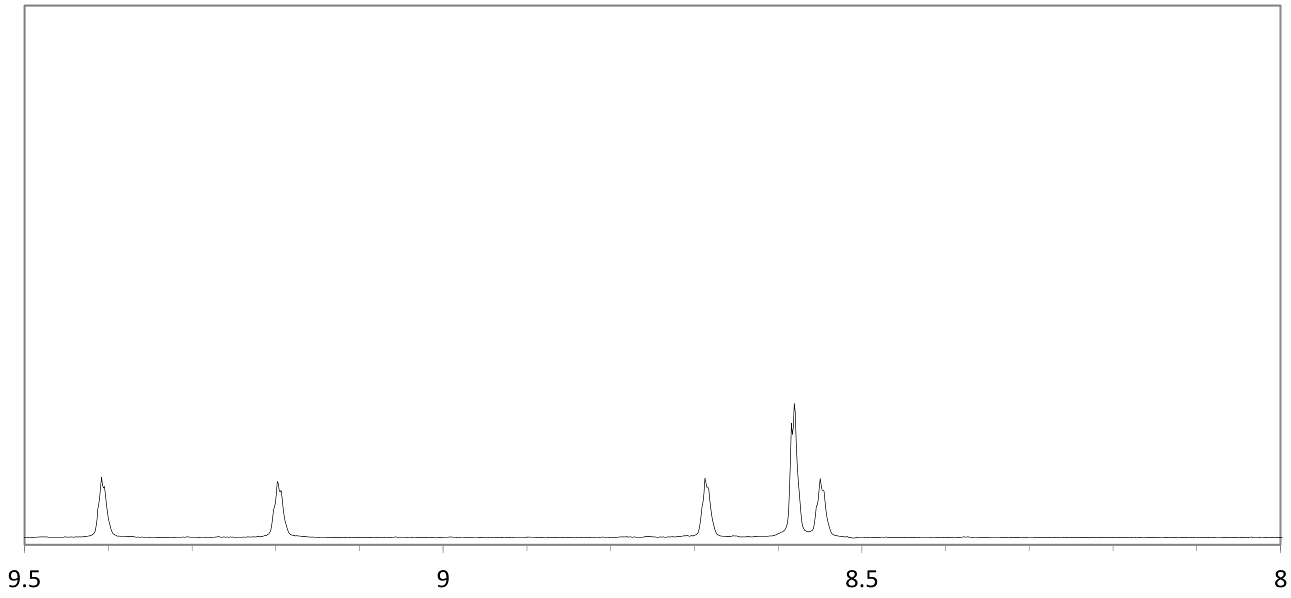
9.5
9
8.5
8

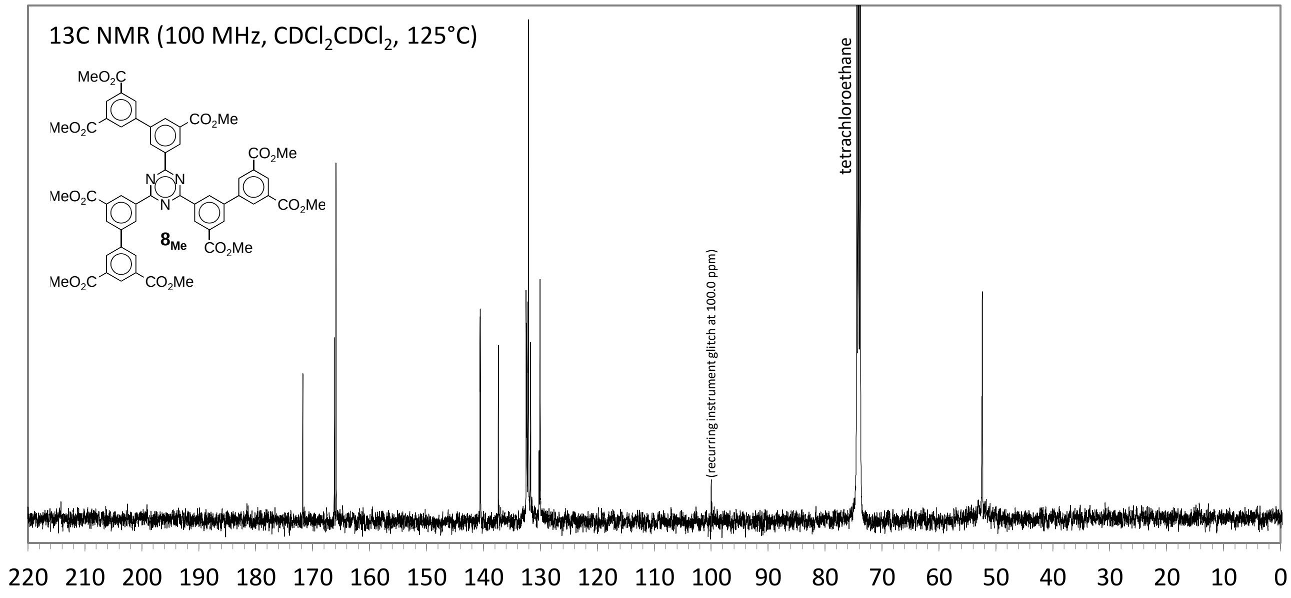
13C NMR (100 MHz, $CDCl_2CDCl_2$, 125°C)
MeO_2C
CO_2Me
N
$\mathbf{8}_{Me}$
tetrachloroethane
(recurring instrument glitch at 100.0 ppm)
220 210 200 190 180 170 160 150 140 130 120 110 100 90 80 70 60 50 40 30 20 10 0

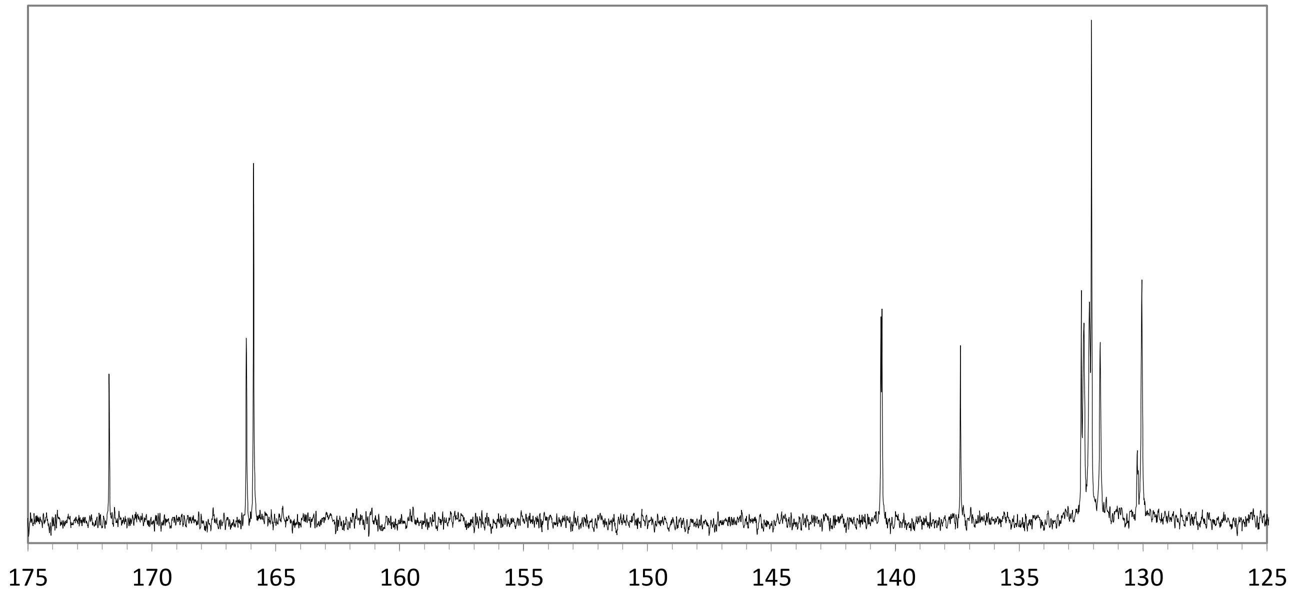
175 170 165 160 155 150 145 140 135 130 125

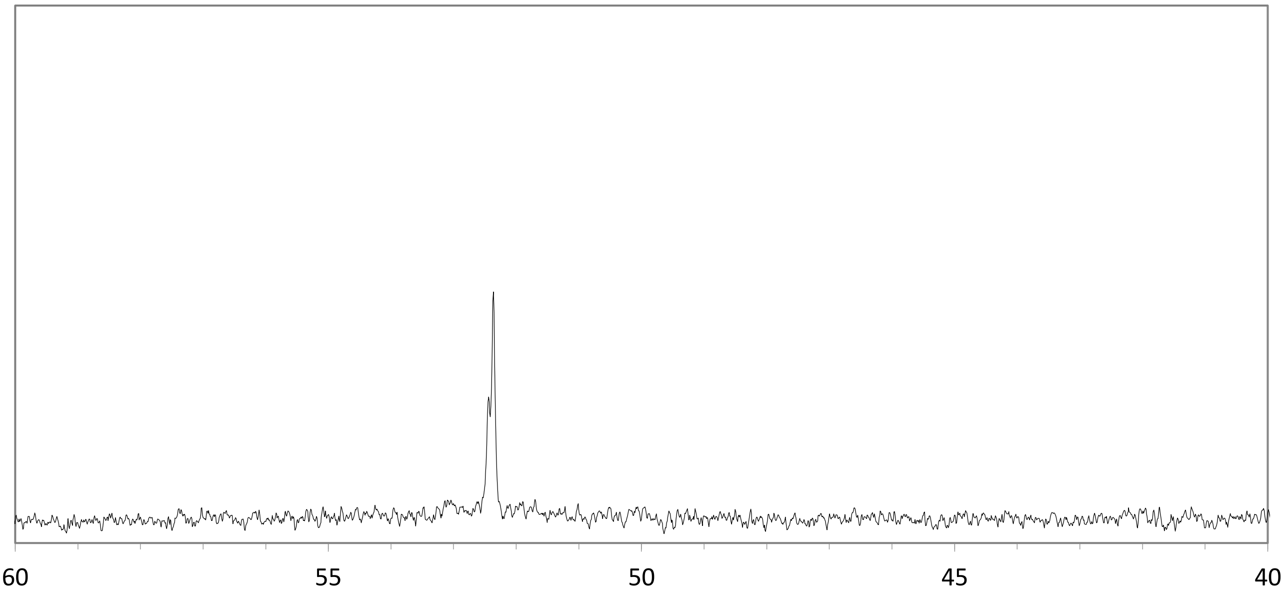
60
55
50
45
40

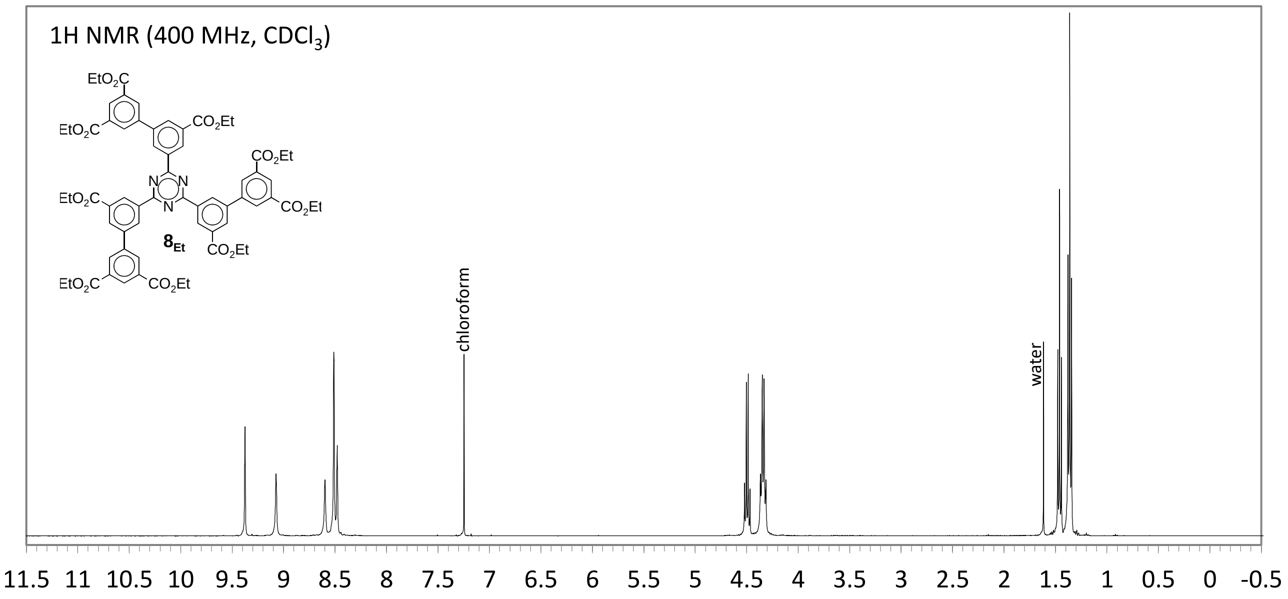
1H NMR (400 MHz, CDCl$_3$)
EtO$_2$C
CO$_2$Et
N
8$_{Et}$
chloroform
water
11.5 11 10.5 10 9.5 9 8.5 8 7.5 7 6.5 6 5.5 5 4.5 4 3.5 3 2.5 2 1.5 1 0.5 0 -0.5

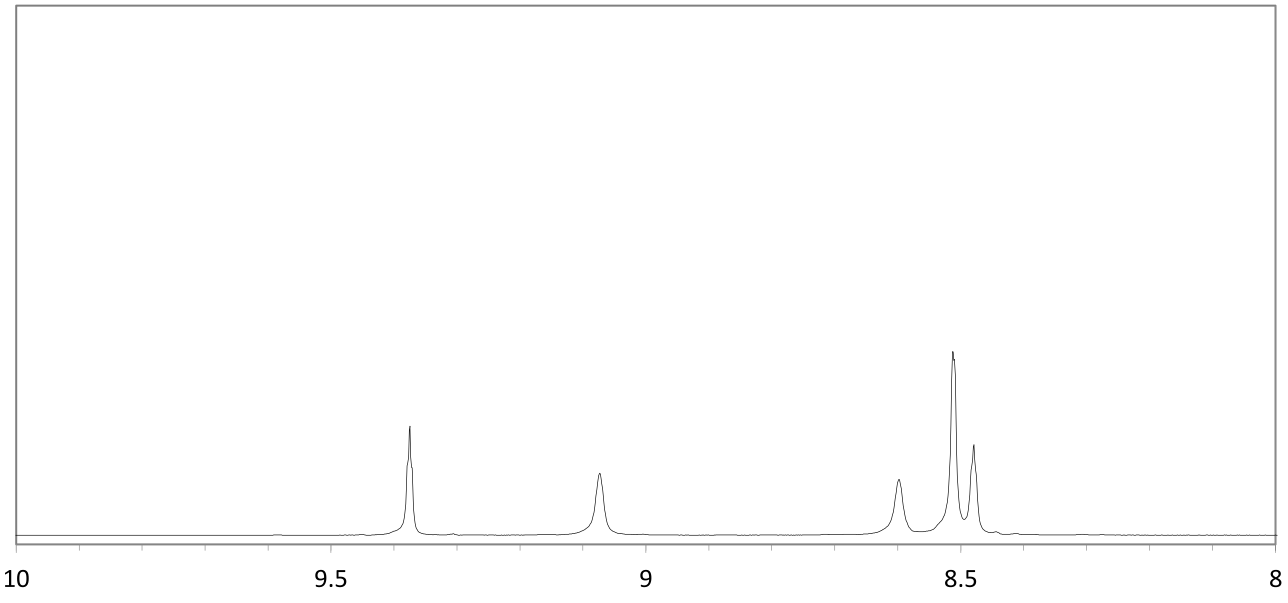
10
9.5
9
8.5
8

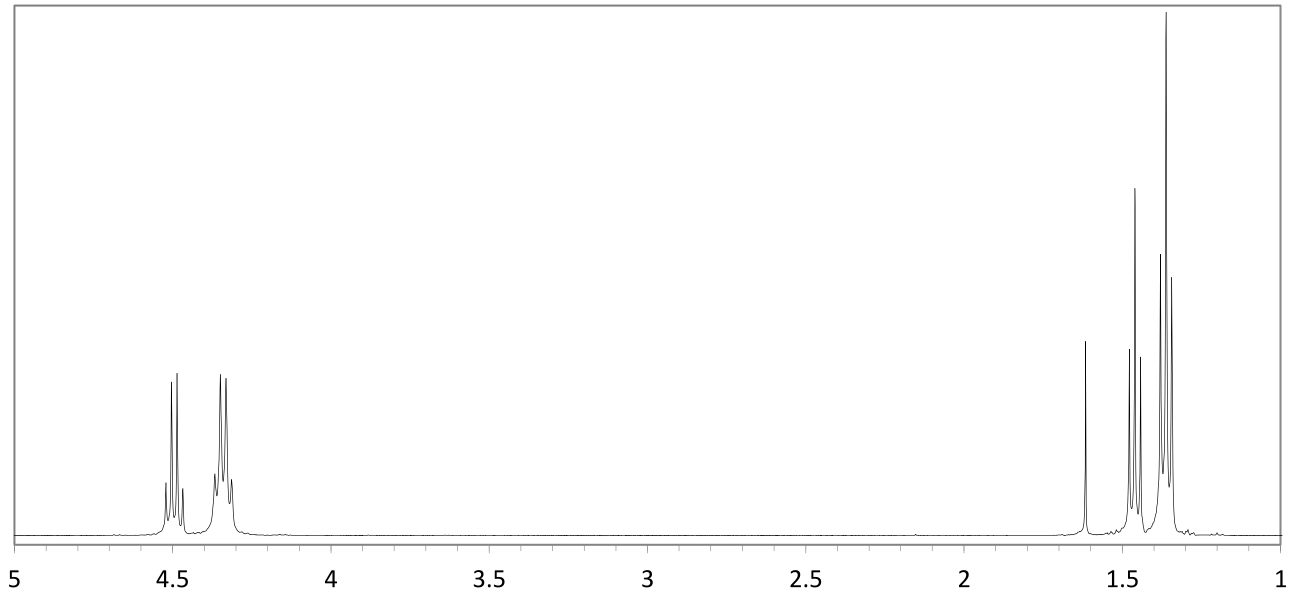
5
4.5
4
3.5
3
2.5
2
1.5
1

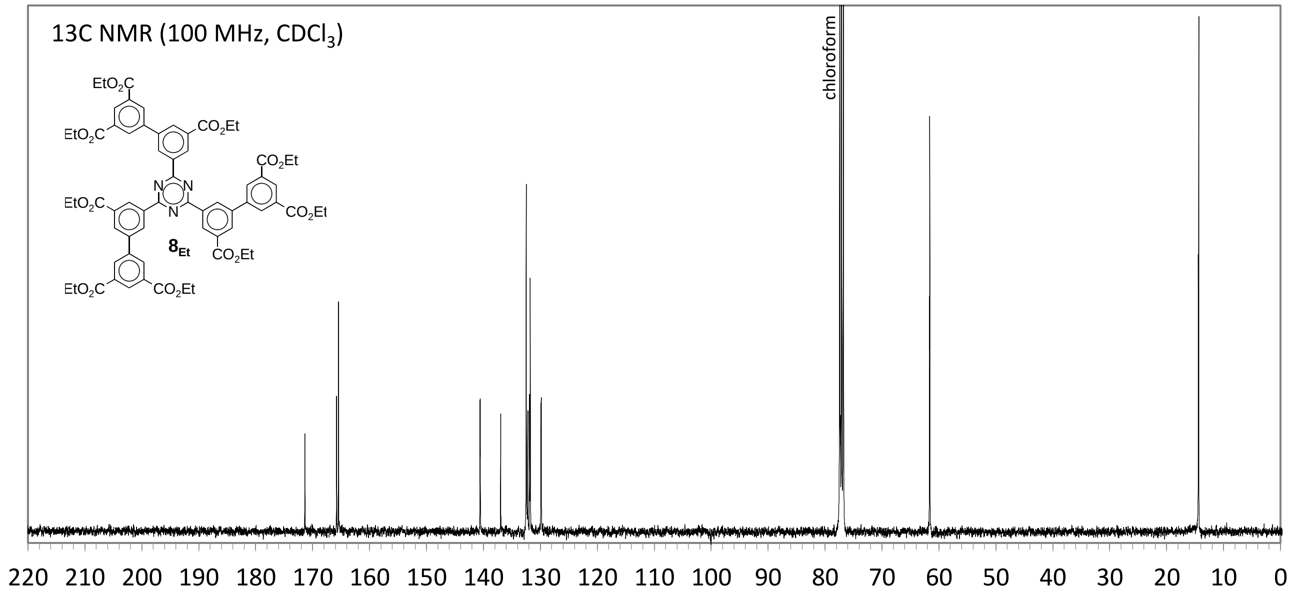
13C NMR (100 MHz, CDCl3)
chloroform
8Et
220 210 200 190 180 170 160 150 140 130 120 110 100 90 80 70 60 50 40 30 20 10 0

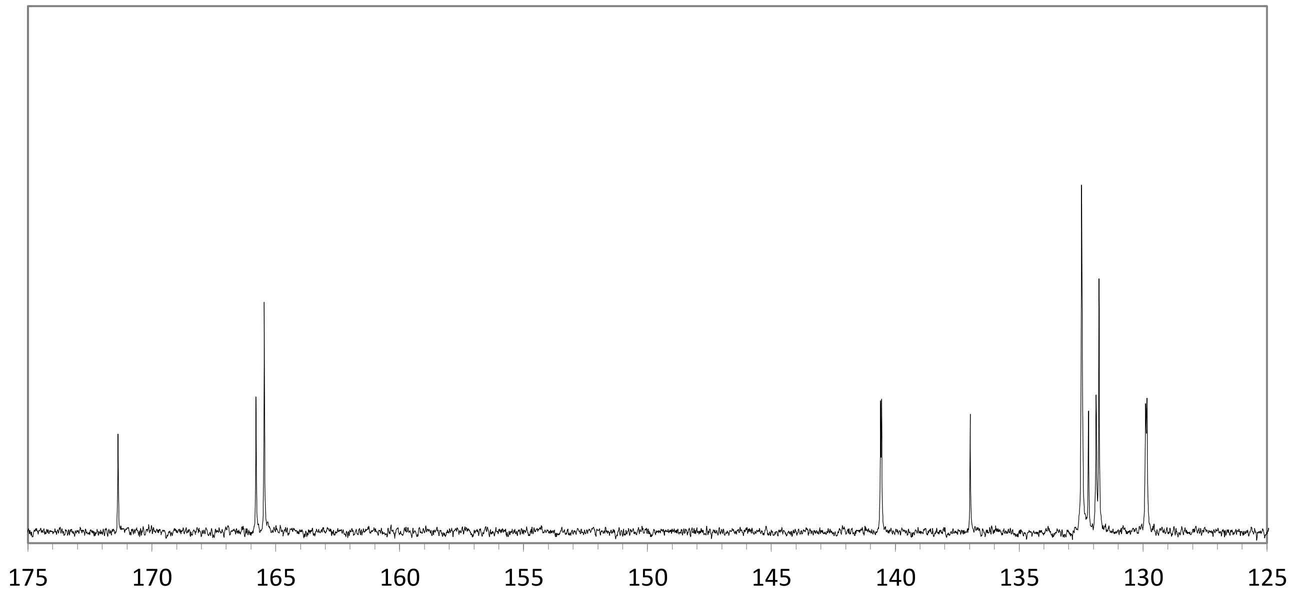
175 170 165 160 155 150 145 140 135 130 125

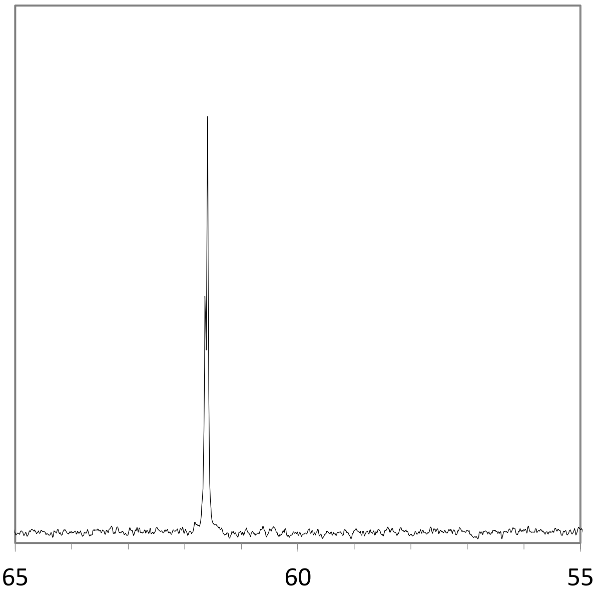

65
60
55

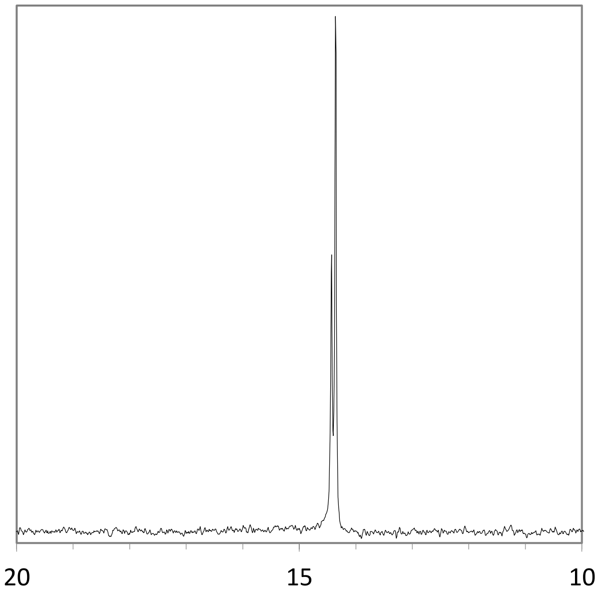

20
15
10

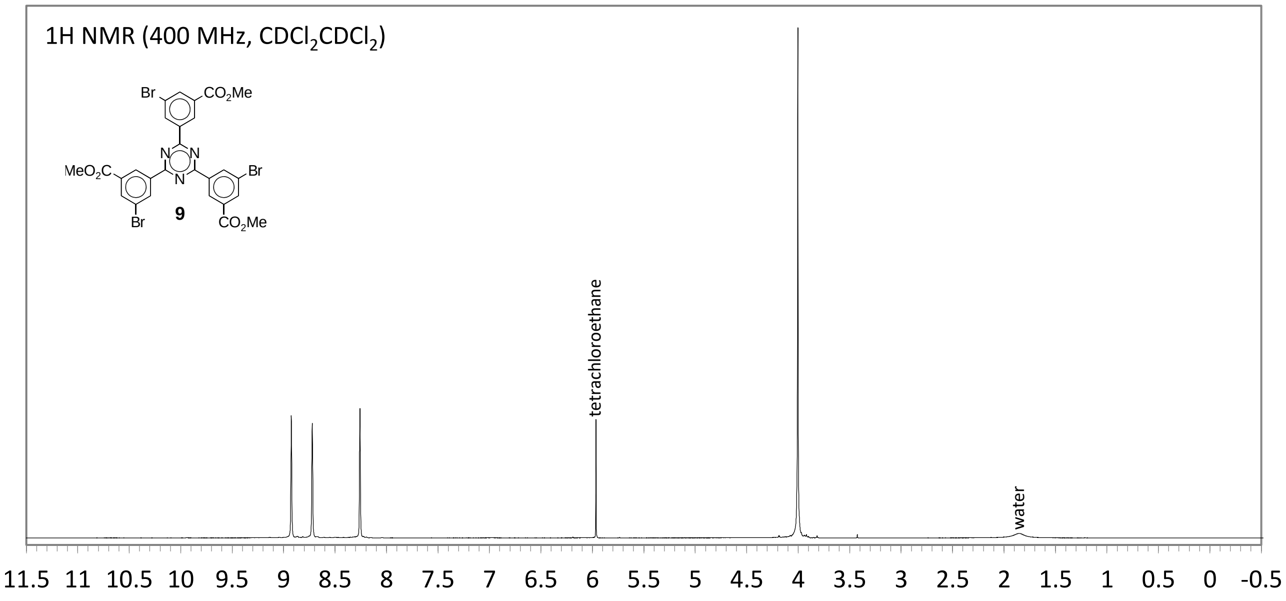

1H NMR (400 MHz, CDCl2CDCl2)
Br
CO2Me
MeO2C
N
Br
9
tetrachloroethane
water
11.5 11 10.5 10 9.5 9 8.5 8 7.5 7 6.5 6 5.5 5 4.5 4 3.5 3 2.5 2 1.5 1 0.5 0 -0.5

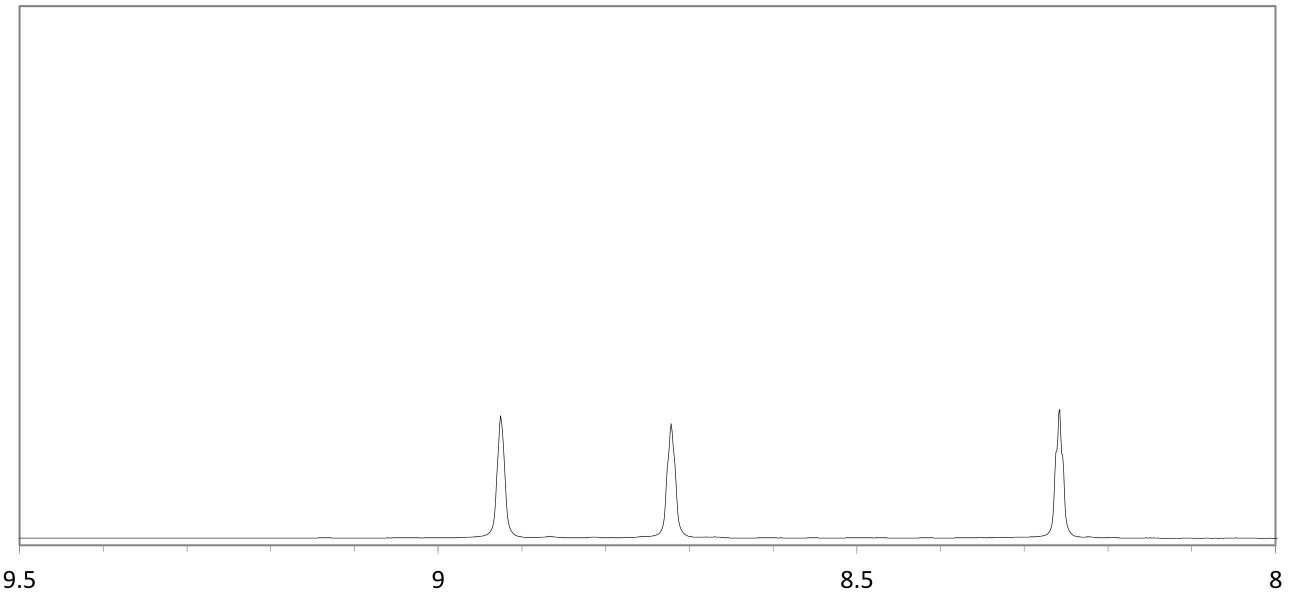

9.5
9
8.5
8

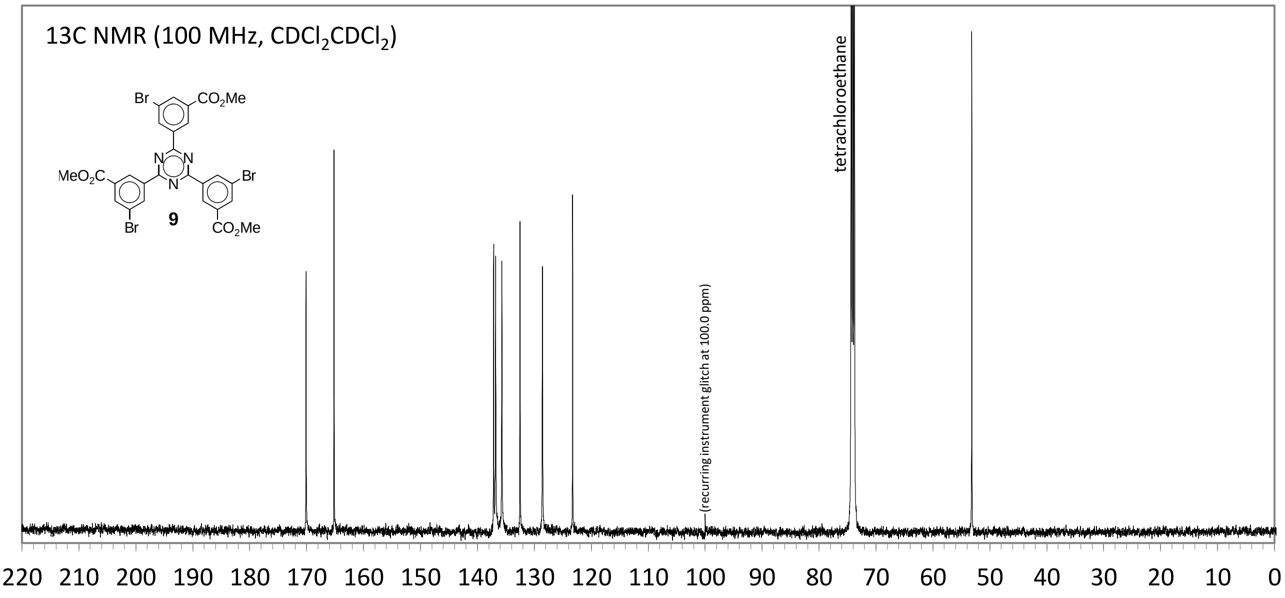
13C NMR (100 MHz, CDCl2CDCl2)
9
tetrachloroethane
(recurring instrument glitch at 100.0 ppm)
220 210 200 190 180 170 160 150 140 130 120 110 100 90 80 70 60 50 40 30 20 10 0

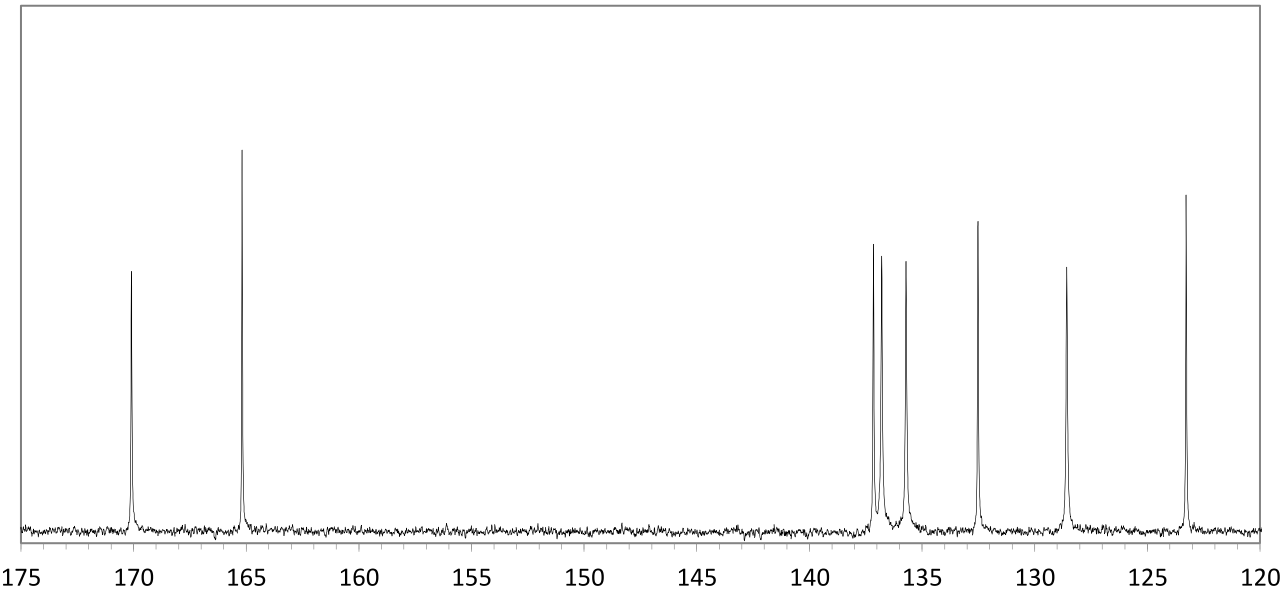
175 170 165 160 155 150 145 140 135 130 125 120

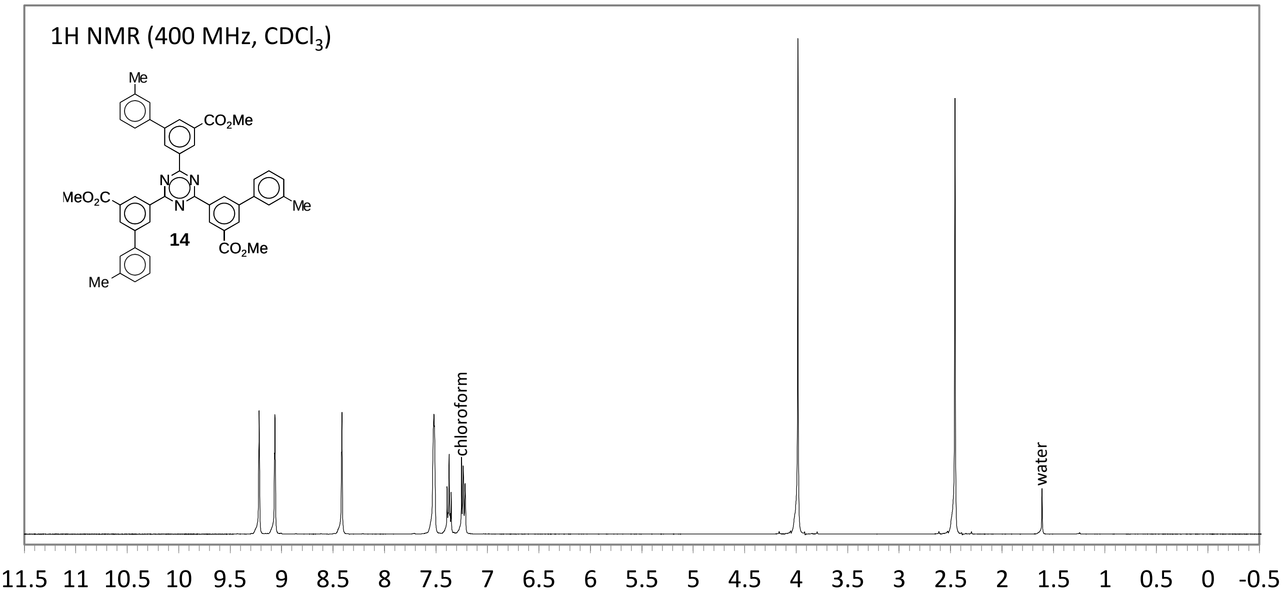
1H NMR (400 MHz, CDCl3)
14
chloroform
water
11.5 11 10.5 10 9.5 9 8.5 8 7.5 7 6.5 6 5.5 5 4.5 4 3.5 3 2.5 2 1.5 1 0.5 0 -0.5

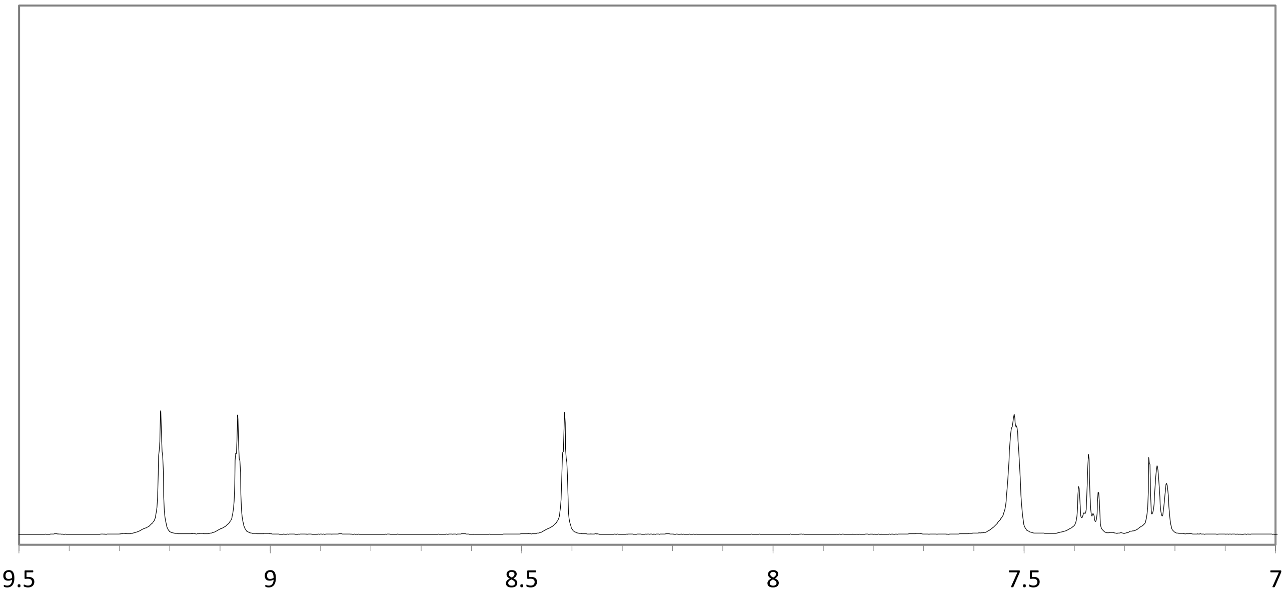
9.5
9
8.5
8
7.5
7

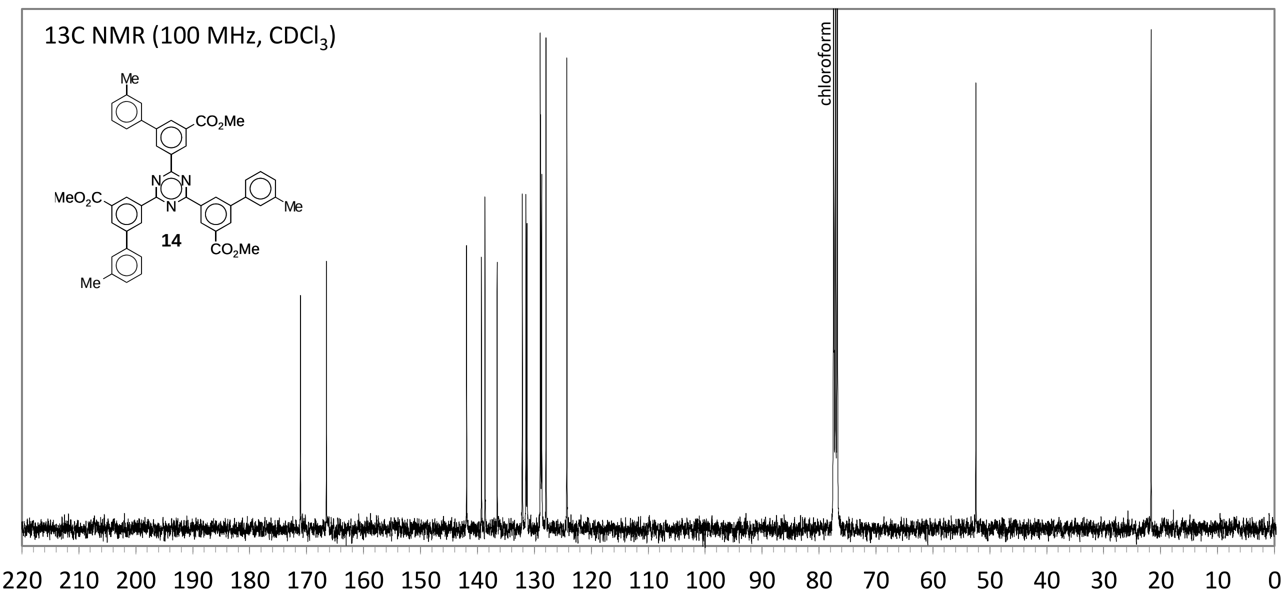
13C NMR (100 MHz, CDCl3)
Me
CO2Me
N
N
N
MeO2C
Me
14
CO2Me
Me
chloroform
220 210 200 190 180 170 160 150 140 130 120 110 100 90 80 70 60 50 40 30 20 10 0

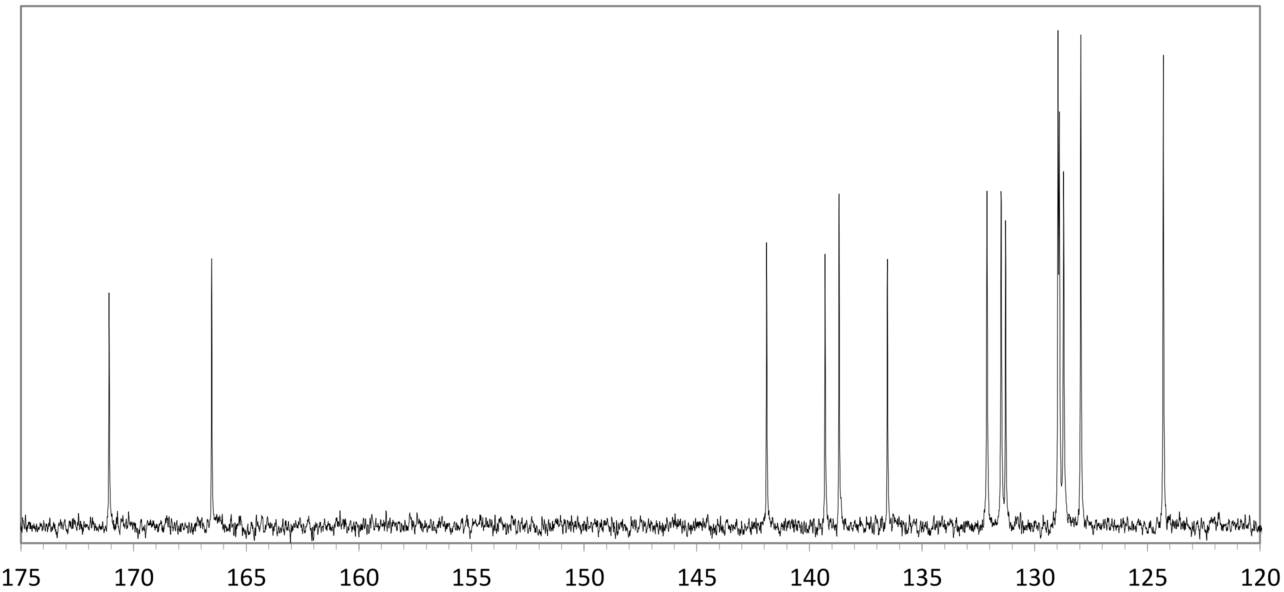
175
170
165
160
155
150
145
140
135
130
125
120

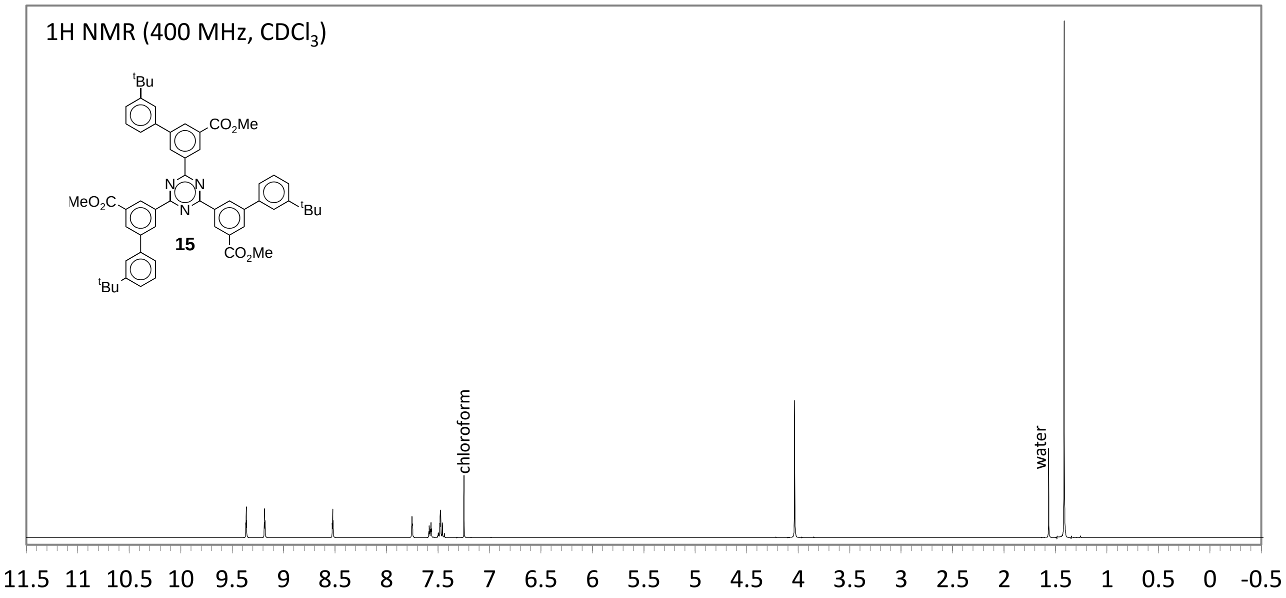
1H NMR (400 MHz, CDCl3)
15
chloroform
water
11.5 11 10.5 10 9.5 9 8.5 8 7.5 7 6.5 6 5.5 5 4.5 4 3.5 3 2.5 2 1.5 1 0.5 0 -0.5

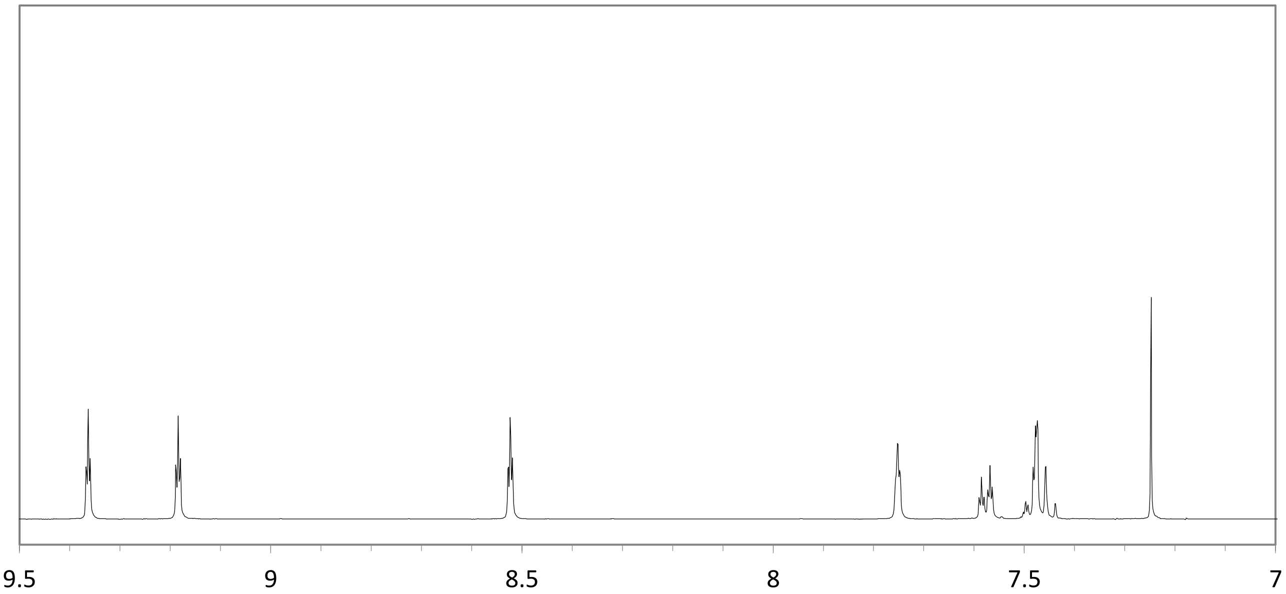
9.5 9 8.5 8 7.5 7

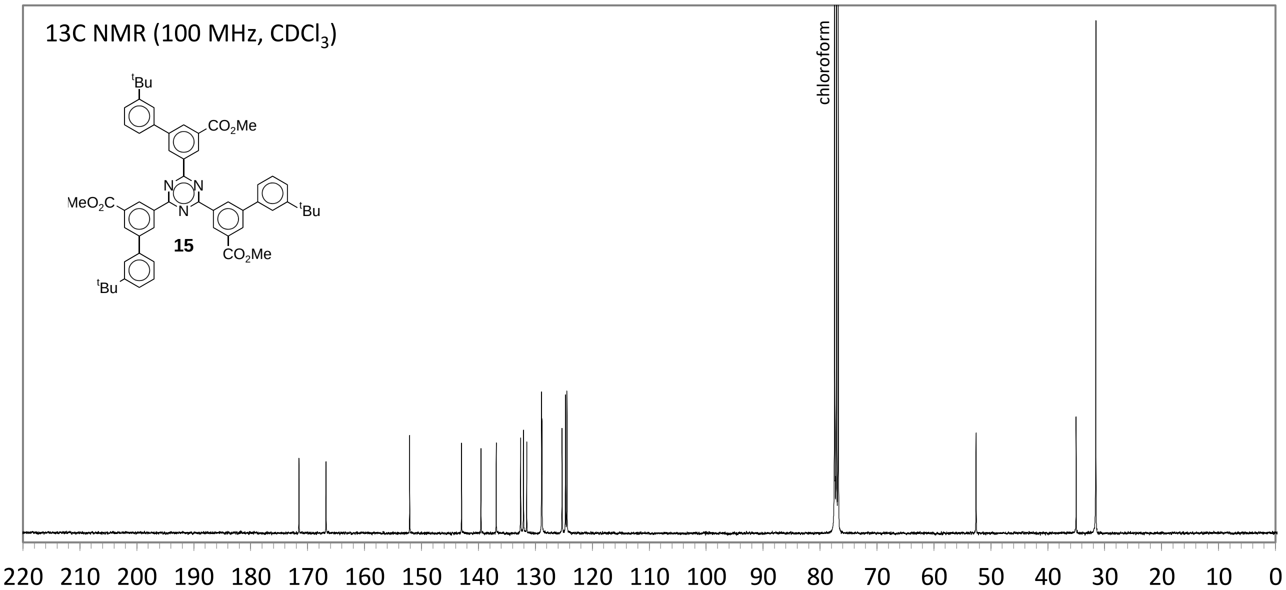
13C NMR (100 MHz, CDCl3)
15
chloroform
220 210 200 190 180 170 160 150 140 130 120 110 100 90 80 70 60 50 40 30 20 10 0

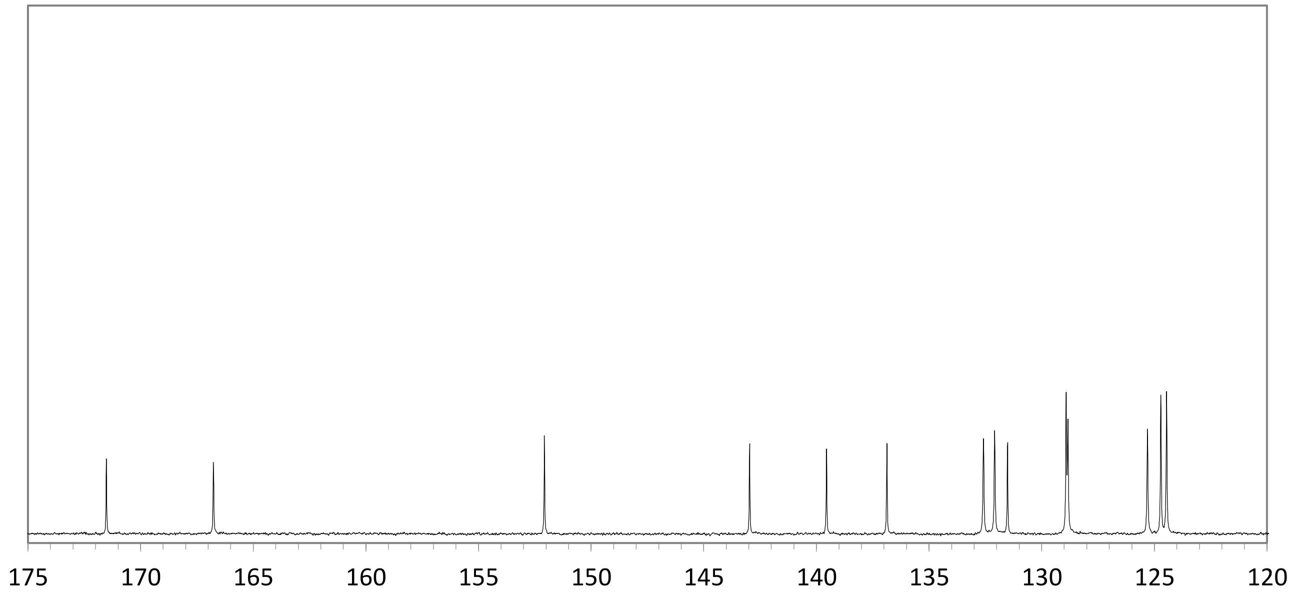
175
170
165
160
155
150
145
140
135
130
125
120

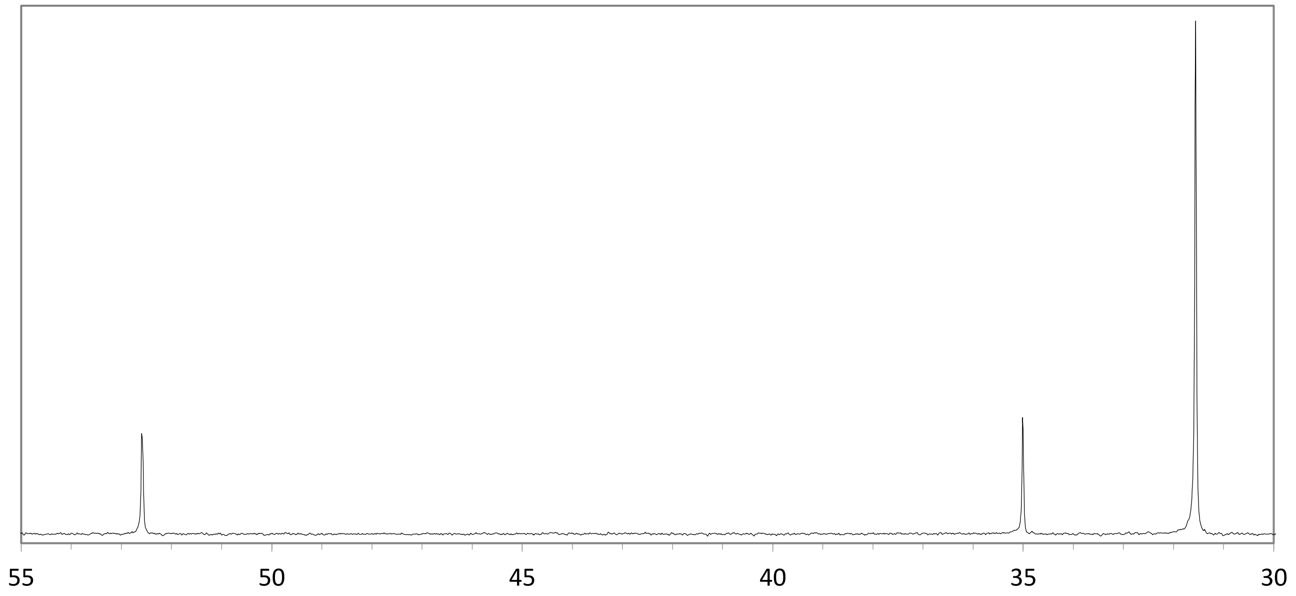
55
50
45
40
35
30

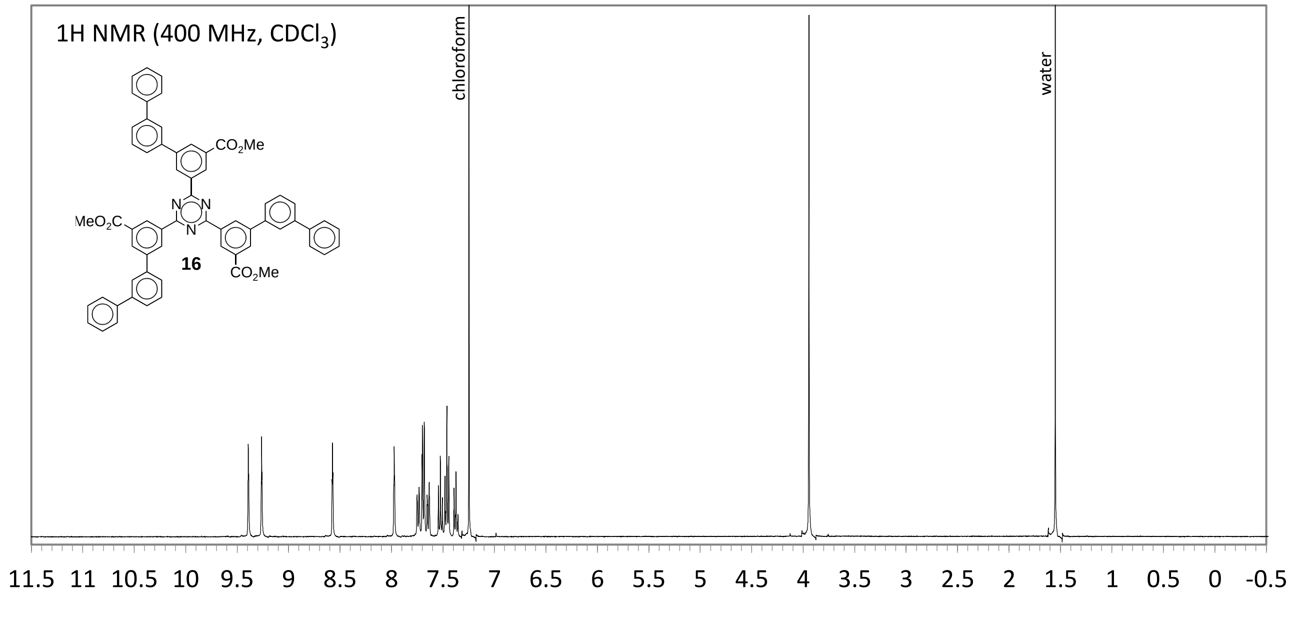
1H NMR (400 MHz, CDCl3)
chloroform
water
CO2Me
MeO2C
N
16
CO2Me
11.5 11 10.5 10 9.5 9 8.5 8 7.5 7 6.5 6 5.5 5 4.5 4 3.5 3 2.5 2 1.5 1 0.5 0 -0.5

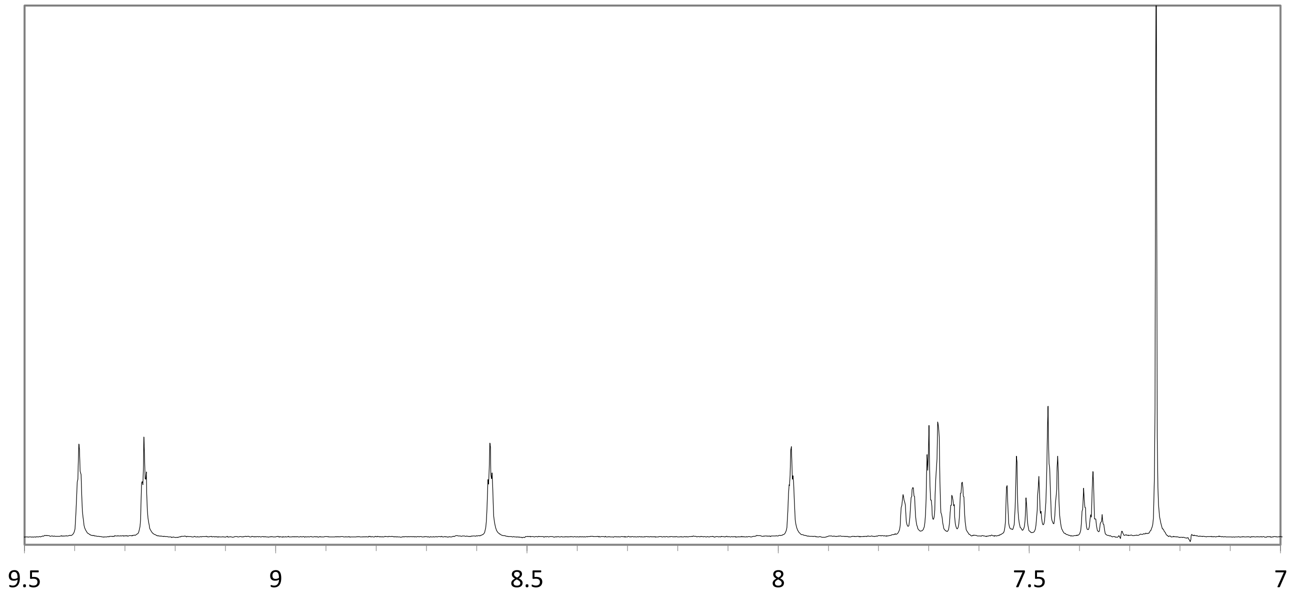
9.5
9
8.5
8
7.5
7

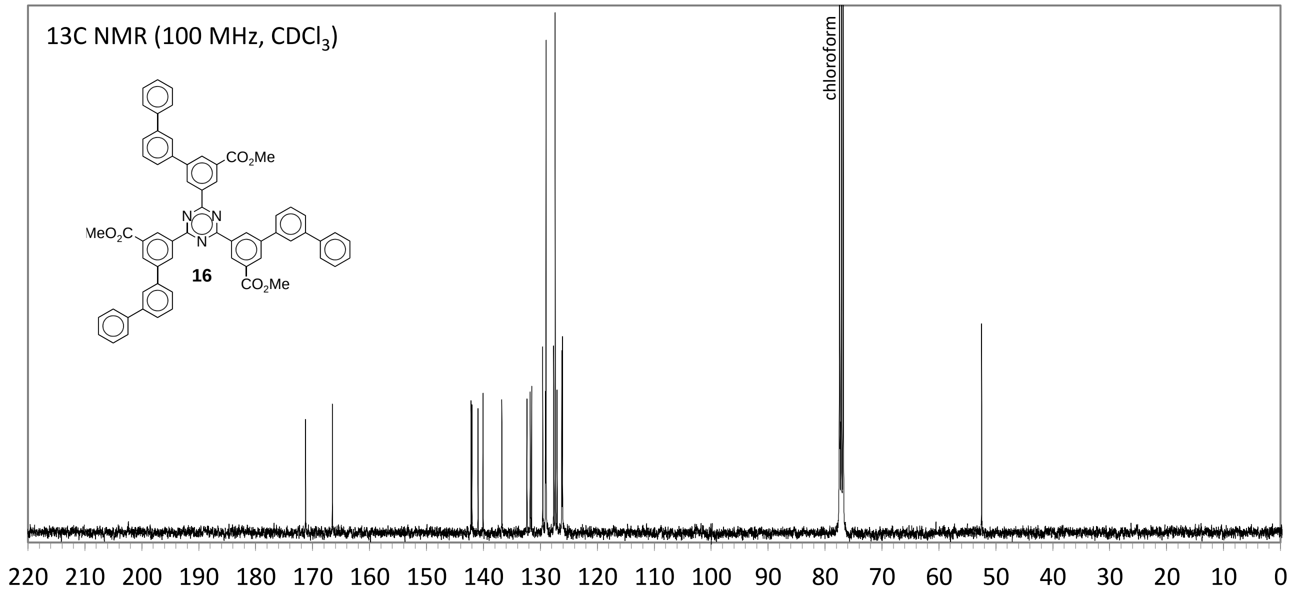
13C NMR (100 MHz, CDCl3)
chloroform
CO2Me
MeO2C
16
CO2Me
220 210 200 190 180 170 160 150 140 130 120 110 100 90 80 70 60 50 40 30 20 10 0

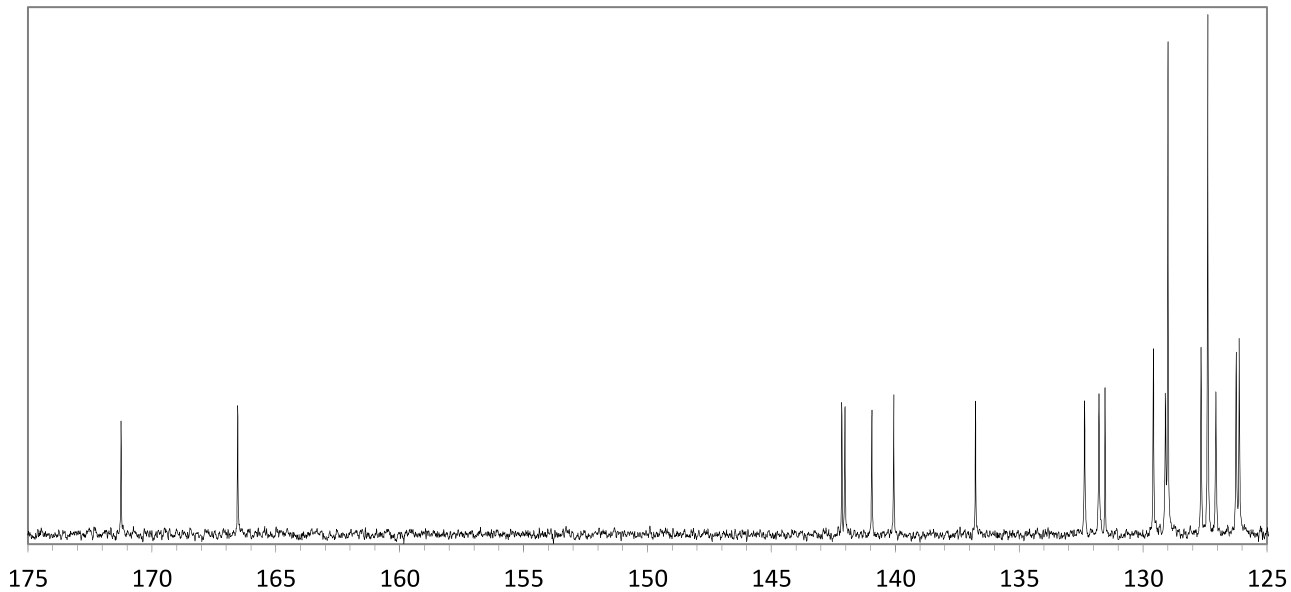
175
170
165
160
155
150
145
140
135
130
125
